# Ferrous and ferric complexes with cyclometalating N-heterocyclic carbene ligands: a case of dual emission revisited†

**DOI:** 10.1039/d3sc02806b

**Published:** 2023-08-29

**Authors:** Catherine Ellen Johnson, Jesper Schwarz, Mawuli Deegbey, Om Prakash, Kumkum Sharma, Ping Huang, Tore Ericsson, Lennart Häggström, Jesper Bendix, Arvind Kumar Gupta, Elena Jakubikova, Kenneth Wärnmark, Reiner Lomoth

**Affiliations:** a Department of Chemistry –Ångström Laboratory, Uppsala University Box 523 SE-75120 Uppsala Sweden reiner.lomoth@kemi.uu.se; b Centre for Analysis and Synthesis, Department of Chemistry, Lund University Box 124 SE-22100 Lund Sweden; c Department of Chemistry, North Carolina State University Raleigh North Carolina 27695 USA; d Department of Physics – Ångström Laboratory, Uppsala University Box 523 SE-751 20 Uppsala Sweden; e Department of Chemistry, University of Copenhagen Universitetsparken 5 DK-2100 Copenhagen Denmark

## Abstract

Iron N-heterocyclic carbene (FeNHC) complexes with long-lived charge transfer states are emerging as a promising class of photoactive materials. We have synthesized [Fe^II^(ImP)_2_] (ImP = bis(2,6-bis(3-methylimidazol-2-ylidene-1-yl)phenylene)) that combines carbene ligands with cyclometalation for additionally improved ligand field strength. The 9 ps lifetime of its ^3^MLCT (metal-to-ligand charge transfer) state however reveals no benefit from cyclometalation compared to Fe(ii) complexes with NHC/pyridine or pure NHC ligand sets. In acetonitrile solution, the Fe(ii) complex forms a photoproduct that features emission characteristics (450 nm, 5.1 ns) that were previously attributed to a higher (^2^MLCT) state of its Fe(iii) analogue [Fe^III^(ImP)_2_]^+^, which led to a claim of dual (MLCT and LMCT) emission. Revisiting the photophysics of [Fe^III^(ImP)_2_]^+^, we confirmed however that higher (^2^MLCT) states of [Fe^III^(ImP)_2_]^+^ are short-lived (<10 ps) and therefore, in contrast to the previous interpretation, cannot give rise to emission on the nanosecond timescale. Accordingly, pristine [Fe^III^(ImP)_2_]^+^ prepared by us only shows red emission from its lower ^2^LMCT state (740 nm, 240 ps). The long-lived, higher energy emission previously reported for [Fe^III^(ImP)_2_]^+^ is instead attributed to an impurity, most probably a photoproduct of the Fe(ii) precursor. The previously reported emission quenching on the nanosecond time scale hence does not support any excited state reactivity of [Fe^III^(ImP)_2_]^+^ itself.

## Abstract 2

Photoluminescence of the Fe^III^ complex occurs only from its ^2^LMCT state while its higher-energy but shorter-lived ^2^MLCT state is non-luminescent. Blue emission arises instead from a photoproduct of the non-emitting Fe^II^ complex.

## Graphical abstract

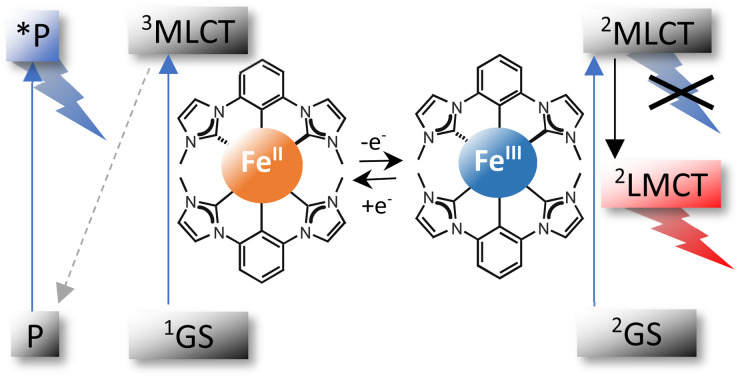

## Introduction

The past decade has seen remarkable progress towards photoactive first-row transition metal complexes^1–3^ by ligand design that has to an increasing extent overcome the problem of ultrafast deactivation of charge transfer states *via* low-lying metal-centered states.^4^ As a result, Earth-abundant metal complexes showing luminescence and excited-state reactivity have become available. They enable the substitution of precious metal photosensitizers and luminophores for the conversion of radiant-to-electric energy or *vice versa* in *e.g.* solar energy conversion and photoredox catalysis^5–8^ or OLEDs.^9,10^ Iron complexes as analogues of the archetypal ruthenium polypyridyl photosensitizers^11^ have since long attracted particular interest^12–20^ and the more recent progress with N-heterocyclic carbene (NHC) ligands^21–28^ has eventually led to ferrous and ferric complexes with demonstrated excited-state electron transfer reactivity of their triplet metal-to-ligand charge transfer (^3^MLCT)^29–35^ and doublet ligand-to-metal charge transfer (^2^LMCT)^36–42^ states, respectively. Furthermore, with lifetimes up to nanoseconds and larger oscillator strengths of the spin-allowed transition between the ^2^LMCT and the doublet ground state, Fe(iii)NHCs can also feature significant photoluminescence quantum yields.^29,43,44^ Emission from the ^3^MLCT states of the Fe(ii)NHCs remains, however, to be demonstrated as it is precluded by current sub-nanosecond lifetimes in combination with the spin-forbidden radiative decay. ^2^MLCT states of Fe(iii) complexes are on the other hand energetically above the ^2^LMCT states and are expected to undergo rapid non-radiative deactivation that should typically preclude any emission from these higher states. We were hence intrigued by the remarkable photophysics of an Fe(iii) complex with the tridentate ligand bis(2,6-bis(3-methylimidazol-2-ylidene-1-yl))phenylene-1-yl (ImP),^45–48^ containing two N-heterocyclic carbenes in conjugation with one cyclometalating arene moiety. The previously described complex [Fe^III^(ImP)_2_]^+^,^45^ was recently reported to feature emission from a higher energy ^2^MLCT state next to the more expected emission from the lowest energy ^2^LMCT state as a first example of dual emission from an FeNHC complex.^49^ Regarding the nanosecond lifetime of the blue emission attributed to the ^2^MLCT state, we were concerned about the absence of an excited state with a corresponding lifetime in the reported transient absorption data of [Fe^III^(ImP)_2_]^+^. Since we have previously found that FeNHC complexes can combine remarkable excited-state properties in both Fe(iii) and Fe(ii) oxidation states of the same complex,^41^ we were enticed to compare the notable photophysical properties reported for [Fe^III^(ImP)_2_]^+^ to those of its hitherto unexplored Fe(ii) analogue.

Here we report on the synthesis, characterization and photophysics of the [Fe^II^(ImP)_2_] complex and have revisited the photophysics of its Fe(iii) analogue. Based on our data, [Fe^III^(ImP)_2_]^+^ lacks the previously reported dual emission while its non-luminescent, photolabile Fe(ii) precursor was identified as a source of a blue-emitting photoproduct.

## Results and discussion

### Synthesis and characterization

**Fig. 1:**
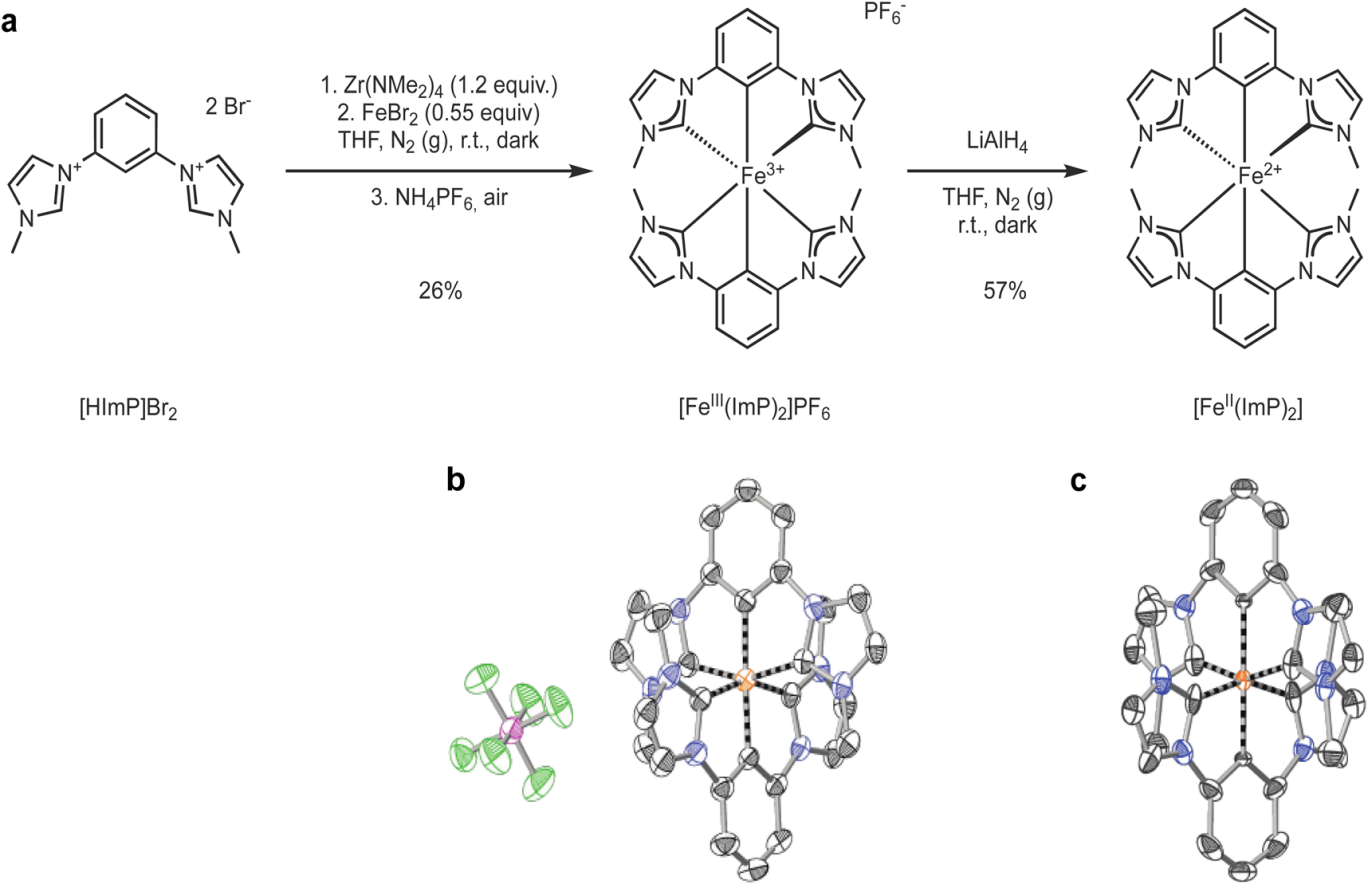
(a) Synthesis of [Fe^III^(ImP)_2_]PF_6_ and [Fe^II^(ImP)_2_] from the ligand precursor 1,1′-(1,3-phenylene)bis(3-methyl-1-imidazolium) dibromide, [HImp]Br_2_. (b) X-ray diffraction structure of [Fe^III^(ImP)_2_]PF_6_ with C in black, F in green, Fe in orange, N in blue, and P in purple. Hydrogen atoms are omitted for clarity. (c) X-ray diffraction structure of [Fe^II^(ImP)_2_].

Pristine [Fe^III^(ImP)_2_]^+^ (ImP = bis(2,6-bis(3-methylimidazol-2-ylidene-1-yl)phenylene)) was synthesized (see ESI for details†) from the ligand precursor 1,1′-(1,3-phenylene)bis(3-methyl-1-imidazolium) dibromide (HImPBr_2_) which was reacted with tetrakis(dimethylamido)zirconium in tetrahydrofuran (THF) under inert atmosphere and in the dark (Fig. 1a).^45^ Transmetalation with FeBr_2_ resulted in the formation of the bis-tridentate complex. Work-up under air was enough to fully oxidize the complex to the Fe(iii) state. The synthesis is similar to that originally described by Hollis and Webster,^45^ and later adapted by Bauer and coworkers,^49^ however differing in several aspects. Our synthesis used the bromide salt instead of the iodide salt of the ligand precursor, [HImP]^2+^, introducing another purification step of the ligand precursor. Similar to Bauer, we used Fe^II^Br_2_, instead of Fe^III^Cl_3_ as Hollis and Webster. We ensured to run the reaction in the dark, but some exposure to ambient light during setup and work-up was allowed. The complex was finally purified by size exclusion chromatography (BioBeads S-X1) to obtain pristine [Fe^III^(ImP)_2_]PF_6_ (see ESI for experimental details†).

[Fe^III^(ImP)_2_]PF_6_ was characterized by NMR, HRMS, elemental analysis and single crystal X-ray diffraction, all consistent with the structure and composition of the complex (Fig. 1b and ESI†). The NMR showed large paramagnetic shifts, with ^1^H-NMR shifts ranging from +25 to −36 ppm and ^13^C-NMR shifts from +520 to −200 ppm. Despite several attempts, it was not possible to detect the carbon resonances directly bound to the iron, presumably due to fast relaxation aided by the paramagnetic iron. The spin state of the complex was further characterized by magnetic susceptibility measurements in solution, solid-state magnetization, and Mößbauer measurements (see ESI†). The paramagnetic susceptibility of [Fe^III^(ImP)_2_]^+^ in acetonitrile was determined by Evans' NMR method to be 2.09 ± 0.04 Bohr magneton (*μ*_B_). In the solid state, the magnetization (*M*) of [Fe^III^(ImP)_2_]PF_6_ shows a variation with the reduced field (BT^−1^) consistent with the solution data, indicating a saturation only slightly above 1 *μ*_B_ (Fig. S3†). The isothermally measured magnetization curves are superimposable in the (BT^−1^) plot, demonstrating the absence of zero field splitting as required for the low spin (LS) t^5^_2g_ ground state. Mößbauer spectra of [Fe^III^(ImP)_2_]PF_6_ at 295 K and 85 K reveal a quadrupole split doublet structure with slight asymmetry, especially for the 85 K spectrum (Fig. S4†). These measurements are all consistent with a d^5^ LS electronic configuration (t^5^_2g_, *S* = 1/2) enforced by the strong field carbene ligands.

[Fe^II^(ImP)_2_] was obtained by reduction of its Fe(iii) precursor [Fe^III^(ImP)_2_]^+^ using LiAlH_4_ in THF solution. The change in oxidation state was accompanied by a drastic color change from dark blue to bright orange. The sensitive product could be isolated and characterized as a diamagnetic (LS d^6^) Fe^II^-complex by NMR, elemental analysis, and single crystal X-ray diffraction (Fig. 1c and ESI†). The Fe^II^ structure is very similar to the Fe^III^ structure, in accordance with what has been observed for other iron NHC complexes isolated in the two oxidation states.^43,50^ The Fe–C distances of 1.93 Å are very similar to those in the related [Fe(pbmi)_2_](PF_6_)_2_ (pbmi = 1,1′-(pyridine-2,6-diyl)bis(methylimidazol-2-ylidene)) complex containing a central pyridine moiety instead of a phenyl anion.^24^

### Photophysics of [Fe^III^(ImP)_2_]^+^

**Fig. 2:**
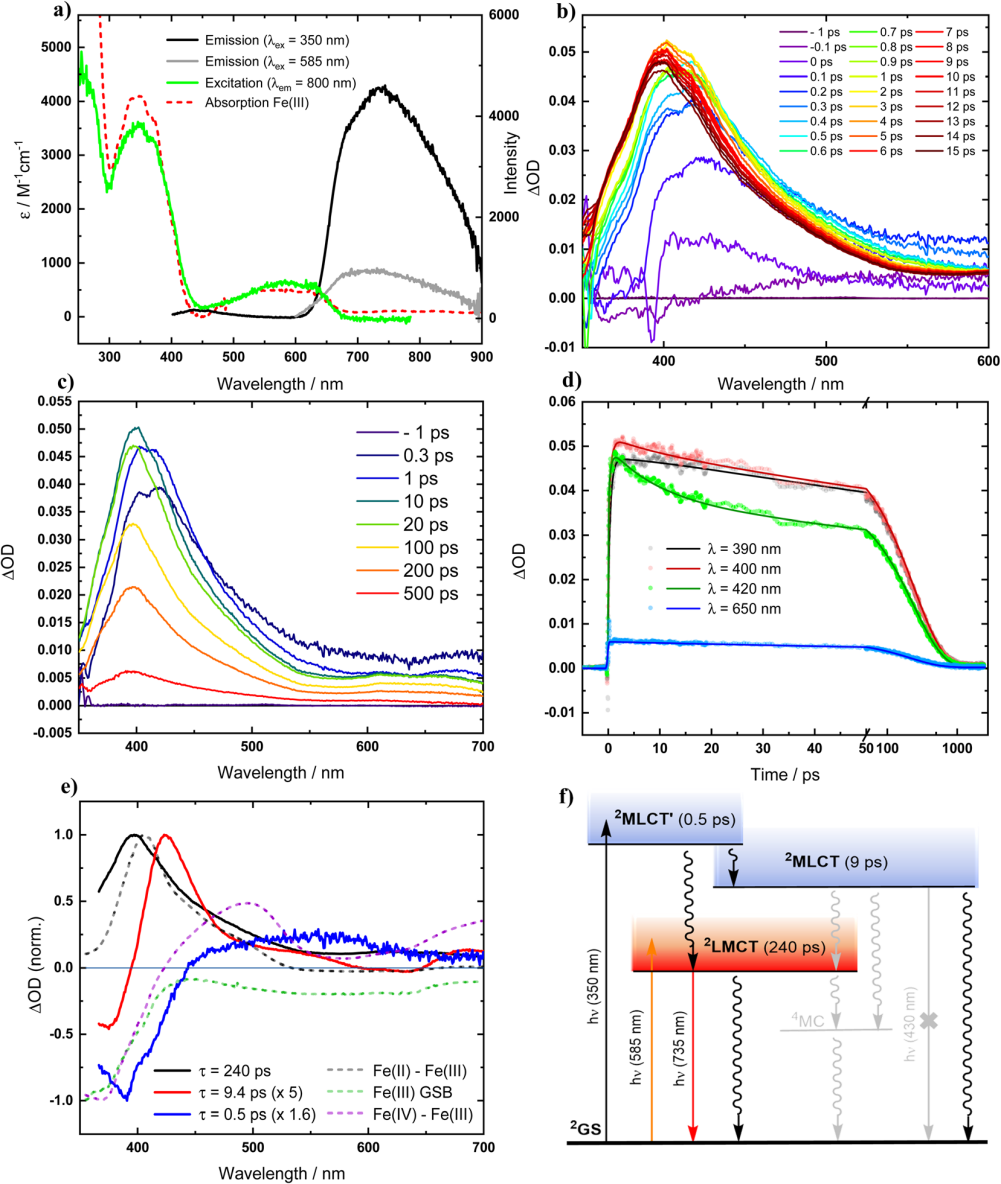
Photophysics of [Fe^III^(ImP)_2_]^+^ in deaerated acetonitrile solution (at room temperature). (a) Absorption, emission and excitation spectra. (b) and (c) Transient absorption spectra at indicated time delays after photoexcitation (*λ*_ex_ = 350 nm, 140 fs). (d) Transient absorption kinetics at indicated wavelengths, fitted by global analysis. (e) Normalized decay-associated spectra (DAS) from global analysis of transient absorption spectra along with the differential absorption spectra for the metal centered reduction and oxidation of the complex and the expected ground-state bleach (GSB). (f) Schematic state energy diagram.

Recognizing that the very oxidation-sensitive [Fe^II^(ImP)_2_] might potentially contain some [Fe^III^(ImP)_2_]^+^ (see below), it was necessary to reassess the photophysical properties of the Fe(iii) complex for reference. The electronic absorption spectrum of [Fe^III^(ImP)_2_]^+^ in acetonitrile (Fig. 2a and S9†) is in good agreement with published data and the previous assignment of the absorption bands at about 600 nm and 350 nm to transitions with strong LMCT and MLCT character, respectively,^49^ is supported by our computational data (Fig. S19†). Photoexcitation of [Fe^III^(ImP)_2_]^+^ into its lowest-energy absorption band (LMCT, *λ*_ex_ = 585 nm) reproduced the reported red emission peaking at around 750 nm with its excitation spectrum matching well with the ground state absorption spectrum (Fig. 2a). Notably, upon excitation of [Fe^III^(ImP)_2_]^+^ into its higher-energy absorption band (MLCT, *λ*_ex_ = 350 nm, Fig. 2a), we observed only a very minor blue emission. The intensity ratio between the blue and red emission bands is very different from the reported data with the dominating blue emission.^49^ Hence, it was evident that the two emission bands cannot be attributed to a dual emission of [Fe^III^(ImP)_2_]^+^, indicating that the blue emission must instead originate from a different species.

In view of the above discrepancy, we also reexamined the excited-state dynamics of [Fe^III^(ImP)_2_]^+^ by fs-transient absorption spectroscopy (fs-TA) in acetonitrile. TA spectra upon excitation of [Fe^III^(ImP)_2_]^+^ in its lowest energy absorption band (*λ*_ex_ = 585 nm) are in good agreement with the reported data^49^ confirming the formation of a ^2^LMCT state with a pronounced 400 nm absorption band characteristic of its Fe(ii) nature and a lifetime of 230 ps (Fig. S12†). The same transient absorption spectrum is observed upon excitation of [Fe^III^(ImP)_2_]^+^ into its higher energy (MLCT) absorption band (*λ*_ex_ = 350 nm). In this case, the ^2^LMCT state is not formed instantaneously but emerges within about a picosecond (Fig. 2b–d) before it decays to the GS with the established lifetime of 240 ps.^49^ For the initially populated excited state which eventually gave rise to the ^2^LMCT state, global analysis returns a lifetime of 0.5 ps. The corresponding decay associated spectrum (DAS) shown in Fig. 2e would be consistent with an excited state of MLCT character that we denote as ^2^MLCT′. The decay of this state not only gives rise to the ^2^LMCT state but leads in parallel to the formation of a third transient as indicated by the 420 nm peak that can be seen next to the 400 nm peak of the ^2^LMCT state during the first few picoseconds. Global analysis returned a lifetime of 9 ps and a DAS (Fig. 2e) that combines features expected for Fe(iii) to Fe(iv) oxidation and a 420 nm peak that we tentatively attribute to the superimposed absorption of a ligand radical anion in an electronically and vibrationally relaxed MLCT state here denoted as ^2^MLCT. Overall, our TA data on [Fe^III^(ImP)_2_]^+^ essentially reproduced the observations presented in the previous report, with a relatively minor difference (6 ps *vs.* 9 ps) for one of the time constants. However, our interpretation of the data, as summarized in the schematic state energy diagram (Fig. 2f), disagrees strongly with the interpretation put forward in the previous report. While it is undebatable that photoexcitation of [Fe^III^(ImP)_2_]^+^ in acetonitrile eventually leads to a relatively long-lived (240 ps) ^2^LMCT state responsible for the red emission, the TA data obviously does not support the existence of any excited state with a lifetime that matches the 4.6 ns decay time of the blue emission described in a previous report.^49^ Instead, the two higher excited states populated upon UV excitation, here formally denoted as ^2^MLCT′ and ^2^MLCT, decay with time constants of 0.5 ps and 9 ps, respectively. The reported assignment of these time constants (0.5 ps and 6 ps in the previous report^49^) to parallel decay paths of the initially excited state leading to the ^2^LMCT state and a long-lived (4.6 ns) ^2^MLCT state disagrees with the TA data and the underlying kinetic scheme. Instead, the decay of the common precursor as well as the parallel formation of the ^2^MLCT and ^2^LMCT product states are described with a single time constant *τ* = 1/∑*k*_i_ where *k*_i_ are the individual rate constants for all decay pathways of the precursor; *i.e*. with the observed 0.5 ps. With the 9 ps (6 ps) decay of the ^2^MLCT state, the assignment of the 4.6 ns emission to this state is evidently inconsistent with the TA data. The TA results hence corroborate our conclusion that the blue emission cannot be attributed to any excited state of [Fe^III^(ImP)_2_]^+^ itself but originates instead from some impurity. Minor quantities of an impurity would readily escape detection by transient absorption spectroscopy but could be easily detectable by its emission. The appealing notion of an emissive Fe(ii) impurity in the form of [Fe^II^(ImP)_2_] is at odds with the relatively high energy of the emission band compared to the lowest energy absorption band of [Fe^II^(ImP)_2_] (Fig. S9† and 3a). Instead, its photophysical characterization (see below) revealed the formation of an emissive photoproduct from the Fe(ii) complex that can contaminate [Fe^III^(ImP)_2_]^+^ prepared from its Fe(ii) precursor and thereby give rise to the blue emission. It is therefore important to note that the reported quenching of the blue nanosecond emission attributed to the ^2^MLCT state of [Fe^III^(ImP)_2_]^+^ has instead to be ascribed to the quenching of emission from an impurity. Reactions involving the non-emissive ^2^MLCT states of [Fe^III^(ImP)_2_]^+^ cannot be monitored by emission quenching and would be limited to conditions eliminating the need for diffusional approach of the reactants during the very short (<10 ps) lifetime at extremely high quencher concentrations or in molecular assemblies. It is only the ^2^LMCT state of [Fe^III^(ImP)_2_]^+^ that provides a lifetime (240 ps) that might be sufficient for reasonably efficient diffusional reactions, but still too short to account for the reported emission quenching on the ns timescale. Despite its lower excited state energy (1.89 eV), it is in theory still rather strongly oxidizing (0.73 V *vs.* Fc^0/+^) and reducing (−1.85 V *vs.* Fc^0/+^) but offers yet no advantages over *e.g.* the ^2^LMCT state of [Fe^III^(phtmeimb)_2_]^+^ (phtmeimb = [phenyltris(3-methyl-imidazolin-2-ylidene-1-yl)borate]^−^) (2 ns, 2.12 eV, 0.97 V, −1.88 V), the Fe(iii)NHC complex having the longest excited state lifetime to date.^29^ Furthermore, as far as reductive quenching cycles of [Fe^III^(ImP)_2_]^+^ are concerned, such applications would need to avoid coordinating solvents in combination with exposure to light with wavelengths <550 nm to safely circumvent degradation of the photolabile Fe(ii) state (see below).

### Photophysics of [Fe^II^(ImP)_2_]

**Fig. 3:**
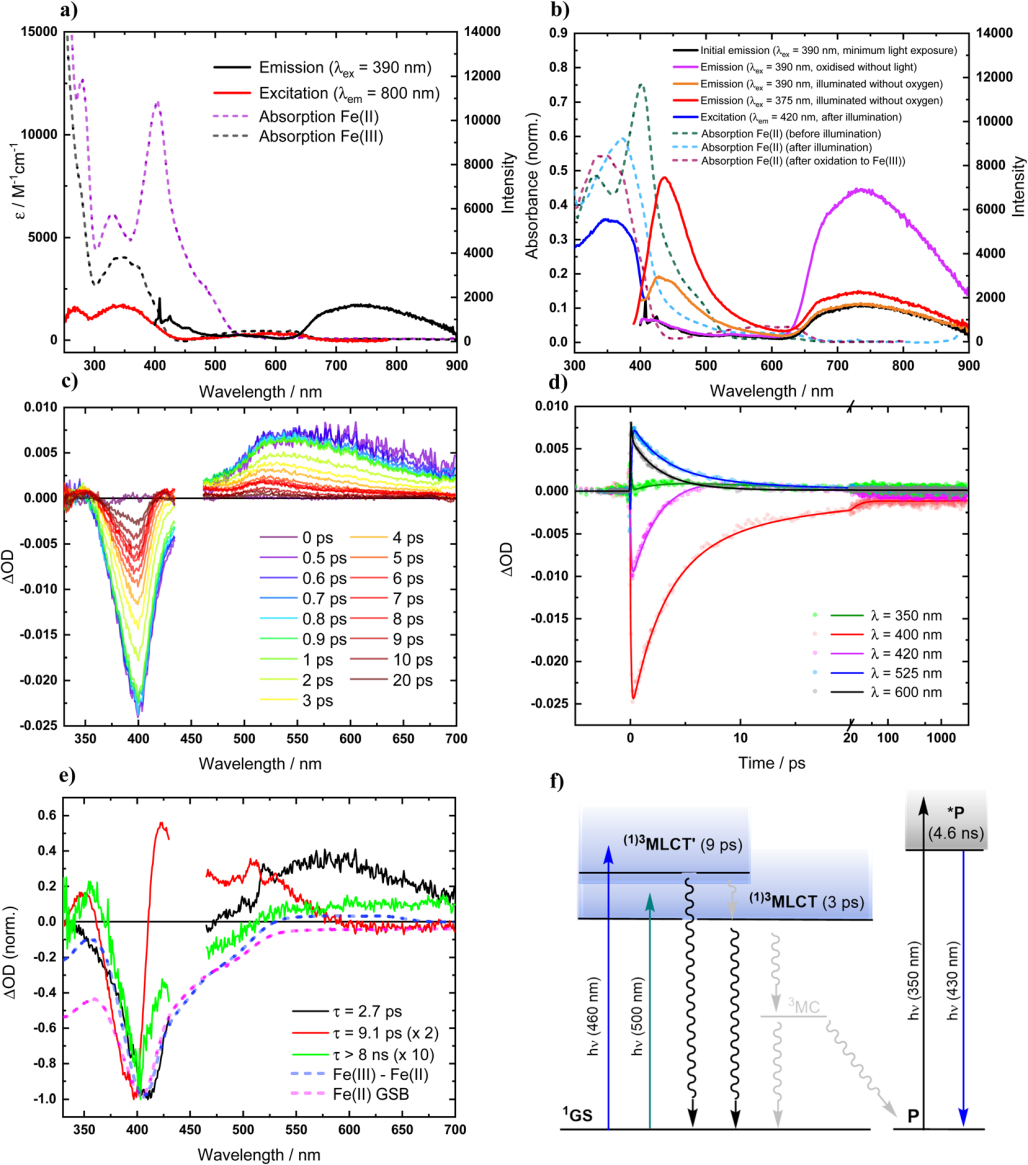
Photophysics of [Fe^II^(ImP)_2_] in deaerated acetonitrile solution (at room temperature). (a) Absorption, emission and excitation spectra. (b) Effects of oxidation (atmospheric O_2_) and light exposure (illumination with white light lamp). (c) Transient absorption spectra at indicated time delays after photoexcitation (*λ*_ex_ = 450 nm, 140 fs). (d) Transient absorption kinetics at indicated wavelengths, fitted by global analysis. (e) Normalized decay-associated spectra (DAS) from global analysis of transient absorption spectra, along with the differential absorption spectra for the metal centered reduction of the complex and the expected ground-state bleach (GSB). (f) Schematic state energy diagram.

**Fig. 4:**
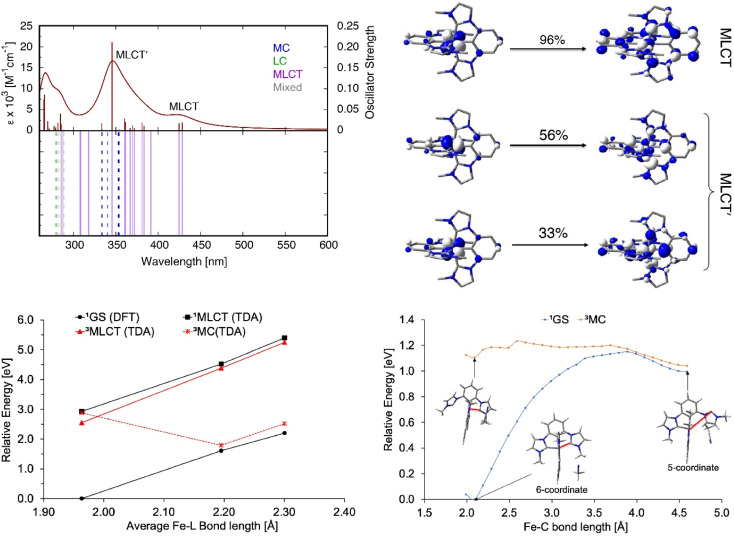
Calculated electronic absorption spectra of [Fe^II^(ImP)_2_]. Top left: UV-Vis spectra of Fe(ii); top right: NTOs for MLCT and MLCT′ transition; bottom left: PECs for Fe(ii); bottom right: PECs for the ligand detachment in ^1^GS and ^3^MC state. Percent contribution of the NTO pairs to the total excitation is given above the arrow. MLCT denotes metal-to-ligand charge transfer, MC metal-centered, LC ligand-centered. The red line denotes the scanned Fe–C bond length.

Absorption spectra of [Fe^II^(ImP)_2_] in acetonitrile obtained by spectroelectrochemistry and by chemical synthesis and isolation, respectively (Fig. 3a and S9†), are in good agreement with each other, but differ significantly from the published spectrum.^49^ Specifically, our spectra unambiguously disprove any absorption above 550 nm and reveal a much more pronounced 400 nm peak. It is hence the latter band with its low-energy shoulder(s) that represent the lowest-energy absorptions of [Fe^II^(ImP)_2_]. MLCT excitation with energies of about 3 eV would be in line with the electrochemical data and computational results corroborating the MLCT assignment. The calculated absorption spectrum shows two absorption bands attributed to MLCT transitions (Fig. 4). The lowest-energy absorption band consists of nearly degenerate MLCT transitions that populate the lowest unoccupied molecular orbital (LUMO) and LUMO+1 orbitals. In contrast, the higher-energy transitions predominantly populate LUMO+3 and LUMO+4 orbitals with distinctly different nodal structures (Fig. S23† for MO pictures), which is also reflected in particle states represented by the natural transition orbitals (NTO) (Fig. 4).

**Fig. 5:**
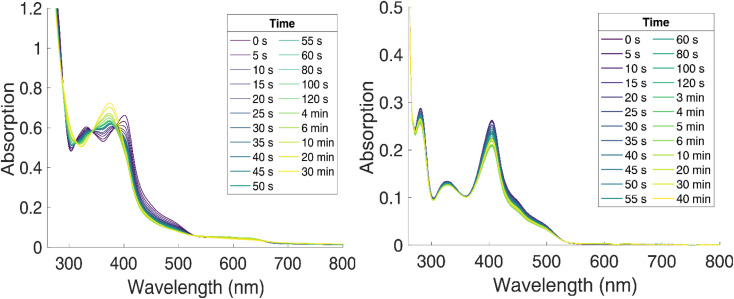
Photostability of [Fe^II^(ImP)_2_] in acetonitrile (left) and THF (right) under N_2_ (g), blue light irradiation (*λ* = 450 nm) at room temperature, monitored by UV-vis absorption spectroscopy.

Excitation of [Fe^II^(ImP)_2_] in acetonitrile in the UV region (350 nm) resulted in the emission spectrum shown in Fig. 3a. The broad red emission band suggests some contamination of the highly oxygen-sensitive Fe(ii) complex with its Fe(iii) congener [Fe^III^(ImP)_2_]^+^. This assignment was further confirmed with the excitation spectrum of the red emission band matching the absorbance spectrum of Fe(iii) and the increase of this emission band upon exposure to oxygen (Fig. 3b). The additional band peaking at about 430 nm matches the blue emission band previously attributed to emission from a higher excited state of [Fe^III^(ImP)_2_]^+^ and also the emission lifetime of 5.1 ns (Fig. S10†) agrees well with the reported value of the reported blue emission from [Fe^III^(ImP)_2_]^+^.^49^ The intensity of the blue emission, however, does not increase together with the red emission upon oxidation (Fig. 3b), supporting our conclusion that [Fe^III^(ImP)_2_]^+^ does not feature any emission in the blue. Instead, the blue emission was found to increase in intensity upon exposure of [Fe^II^(ImP)_2_] to light indicating that the emissive species is a product of a photoreaction (Fig. 3b). Corresponding absorption changes can also be observed, with a well-defined product absorption peak in the UV (370 nm) that agrees with the excitation spectrum for the blue emission (Fig. 3b and 5a). Control experiments in the dark only showed a slow oxidation to the Fe(iii) state (Fig. S5†). While samples of [Fe^II^(ImP)_2_] inevitably contain some [Fe^III^(ImP)_2_]^+^, any contribution of the latter to the formation of photoproduct(s) can be excluded based on the pronounced photostability of the Fe(iii) complex (Fig. S6†). The detailed structure of the emissive species remains unclear at the current stage. However, similar emission (410 nm, 1.7 ns, 4.6 ns) and absorption (365 nm) characteristics were found for the ImP ligand precursor [HImp]^2+^ (Fig. S11†) suggesting that the emissive species might be some ligand derivative. Furthermore, solutions of [Fe^II^(ImP)_2_] in THF showed weaker signs of photobleaching without any discernible product absorption (Fig. 5b) indicating some involvement of the solvent in the photodegradation.

The potential energy curves (PECs) for the relevant electronic states of the [Fe^II^(ImP)_2_] complex were also computed (Fig. S27†), revealing the ^3^MLCT to be the lowest-energy excited state, with the ^3^MC (metal-centred) and ^5^MC states 0.33 and 1.82 eV higher in energy, respectively, at the ground-state optimized geometry. At the relaxed geometry of the ^3^MC, it lies below the ^3^MLCT, suggesting a possibility of internal conversion. Structures of the ^3,5^MC states optimized with the density functional theory (DFT) show a partial ligand detachment, suggesting a possibility of the solvent (acetonitrile) coordination in the excited state (Fig. S28–S31 and ESI for further details†). To further examine the partial ImP ligand detachment, we looked at interactions between [Fe^II^(ImP)_2_] and acetonitrile, in which a single molecule of acetonitrile coordinates to the Fe center upon the partial ligand detachment (“head-on” coordination) or inserts itself into the pocket created by the detached ligand, weakly interacting with the 5-coordinate iron complex (“van der Waals” coordination, see Fig. S28†). We found that although both head-on and van der Waals coordination are thermodynamically uphill for ^1^GS, in the ^3^MC and ^5^MC states the 5-coordinate complex with a partially detached ligand is stabilized through van der Waals coordination of the acetonitrile solvent molecule (Table S10†). The one-dimensional PEC scans connecting the 6-coordinate and 5-coordinate [Fe^II^(ImP)_2_]-acetonitrile structures show that the partial ligand detachment and acetonitrile coordination are unfavorable in the ground state (the 5-coordinate structure is ∼1 eV or 23 kcal mol^−1^ higher in energy than the 6-coordinate structure, with a transition state of ∼1.1 eV or 25 kcal mol^−1^). In contrast to this, the optimized 6-coordinate structure of the ^3^MC state already features one lengthened Fe–C bond at ∼3.2 Å, and van der Waals coordination of acetonitrile is favored and easily accessible (Fig. 4). Moreover, the ^1^GS and ^3^MC states lie near each other in energy at the 5-coordinate geometry, suggesting a possibility of a nonradiative decay from ^3^MC to ^1^GS at this geometry, with a subsequent conversion back to a 6-coordinate [Fe^II^(ImP)_2_] complex (an estimated transition state for this conversion is ∼0.1 eV or 2 kcal mol^−1^, see Fig. 4), reversing the partial ligand detachment occurring upon irradiation with light. The formation of the 5-coordinate complex could also represent the first step in the formation of a different photoproduct (see above) and more investigation would be necessary to fully explore this issue.

The excited state dynamics of [Fe^II^(ImP)_2_] in acetonitrile were studied with excitation at 450 nm to avoid excitation of any [Fe^III^(ImP)_2_]^+^ potentially present in the sample. A moving sample stage was employed to limit any effects of irreversible photobleaching or accumulating photoproducts. Good agreement with the TA data obtained in THF solution (Fig. S13–S15†) where [Fe^II^(ImP)_2_] is more photostable confirmed that with this approach, the TA data collected in acetonitrile can also be attributed to intact [Fe^II^(ImP)_2_]. The initial transient absorption spectra show a ground state bleach signal at 400 nm and broad transient absorption peaking between 500 nm and 600 nm, which would be consistent with the bleach expected for the Fe(ii) to Fe(iii) oxidation together with the ligand anion radical absorption of an MLCT state (Fig. 3c and d). Global analysis reveals two major components with lifetimes of 2.7 and 9.1 ps respectively and their normalized DAS are shown in Fig. 3e. The former component is responsible for the faster decay of the absorption >550 nm, while the latter describes the somewhat more long-lived TA below 550 nm, including the more slowly recovering portion on the blue side of the ground state bleach. These features are attributed to two MLCT states with probably not only different degrees of vibrational and solvent relaxation but also different electronic structures in agreement with the computational data. As intersystem crossing can be expected to occur within the instrument response function (IRF) of the TA experiments (<140 fs) and hence would not be resolved,^51,52^ these states are denoted as ^3^MLCT′ and ^3^MLCT, respectively (Fig. 3f). Their short lifetimes established by TA spectroscopy confirm that intact [Fe^II^(ImP)_2_] is not responsible for the blue nanosecond emission. The kinetics at 420 nm indicate that the ^3^MLCT forms at least partly concomitant with the decay of ^3^MLCT′ while a portion of the two states may be formed in parallel during the IRF. Notably, the ligand radical contributions of both ^3^MLCT states resembles the corresponding spectral features of the two ^2^MLCT states of the Fe(iii) complex where they appear next to the Fe(iii) to Fe(iv) oxidation features (Fig. 2e and 3e). A minor part of the ground state bleach after excitation of [Fe^II^(ImP)_2_] does not recover within the 8 ns time window of the TA experiments. The remaining TA spectrum is consistent with a bleaching of the ground state absorption or alternatively oxidation to the Fe(iv) state and therefore attributed to some irreversible photochemistry. Correspondingly, these irreversible changes were even less prominent in THF where otherwise very similar TA data was obtained with essentially identical spectra and only minor variations in the time constants (4.9 *vs.* 2.7 ps and 8.4 *vs.* 9.1 ps) (Fig. S13–S15†). In THF solution, TA data was also collected with 500 nm excitation of [Fe^II^(ImP)_2_], *i.e.*, at the red edge of its lowest energy absorption band. Under these conditions the TA data greatly simplifies as only the characteristics of a ^3^MLCT state with a lifetime of 5.4 ps are observed next to the very minor irreversible ground state bleach. A ^3^MLCT excited state with a lifetime of a few ps is comparable to most Fe(ii) complexes with NHC ligands that have excited state energies close to or equal to 2 eV. Together with the low potential of its Fe(iii/ii) couple, [Fe^II^(ImP)_2_] is a particularly strong excited state reductant (*E*^III/II^_1/2_* < −3 V) that might even reduce the acetonitrile solvent but applications will be hampered by the rather short ^3^MLCT lifetime even if the photolability of the complex can be circumvented in suitable media such as THF. Substantially longer lifetimes of pure Fe ^3^MLCT states of Fe(ii) complexes have so far only been observed for a hexa-NHC complex [Fe(btz)_3_](PF_6_)_2_ (0.5 ns),^50^ a double cyclometalated phenanthroline complex (1 ns)^53^ and benzannulated diarylamido complex (3 ns).^54^ The latter complexes are however characterized by rather low energies of their ^3^MLCT states (*ca.* 1–1.2 eV and 1.2 eV) which might be a major factor behind their slower deactivation *via* MC states.

## Conclusions

We have synthesized and characterized the Fe(ii) and Fe(iii) complexes [Fe^II^(ImP)_2_] and [Fe^III^(ImP)_2_]^+^ with cyclometalating NHC ligands and have investigated the photophysical properties of the Fe(ii) complex and revisited those recently reported for its Fe(iii) analogue [Fe^III^(ImP)_2_]^+^ by experimental and computational techniques. For the Fe(ii) complex, the moderate lifetime of its ^3^MLCT state (9 ps in acetonitrile) indicates that the cyclometalating ligand in this case does not provide the expected advantage over previously used NHC/pyridine or pure NHC ligand sets when it comes to the destabilization of metal-centered states. Notably, our results for [Fe^II^(ImP)_2_] revealed the formation of a luminescent product from the photodegradation of the intrinsically non-luminescent Fe(ii) complex. The spectral characteristics and 5.1 ns lifetime of the emission from this product are near identical to the blue emission previously attributed to the emission from a higher (^2^MLCT) state of its Fe(iii) analogue [Fe^III^(ImP)_2_]^+^. For the Fe(iii) complex itself, our results confirm however that its higher (^2^MLCT) excited states are short-lived (<10 ps) and therefore cannot give rise to the reported blue emission with its nanosecond lifetime. Accordingly, pristine [Fe^III^(ImP)_2_]^+^, synthesized by our protocol that eliminates contamination with photoproducts of the Fe(ii) precursor, only shows red emission from its lower (^2^LMCT) state. Regarding applications of [Fe^III^(ImP)_2_]^+^ as a photosensitizer, the previously reported quenching of the nanosecond emission does not demonstrate any excited state reactivity of [Fe^III^(ImP)_2_]^+^ itself but has to be instead attributed to the quenching of the impurity's emission. Generally, these results illustrate the challenges with the assignment of the sometimes rather weak emission from coordination compounds, particularly in the blue part of the spectrum where already minor impurities in the form of *e.g.*, simple organics can give rise to significant emission upon UV excitation.

## Data availability

Crystallographic data for [Fe^II^(ImP)_2_] and [Fe^III^(ImP)_2_]PF_6_ have been deposited as CCDC 2254082 and 2254083. Additional experimental details and data are provided in the ESI,† including synthesis, single-crystal XRD, NMR, HRMS, Mößbauer and electronic absorption spectra, electrochemistry and spectroelectrochemistry, magnetic susceptibility and magnetization, TCSPC and fs-TA, stability measurements and DFT calculations.

## Author contributions

J. S., O. P and K. S. carried out the synthesis of the compounds. J. S. and O. P. did the synthesis characterization of the compounds. C. J. conducted the steady state and time-resolved photophysical measurements, carried out the electrochemical measurements, and analyzed the data. M. D. carried out the quantum chemical investigations and analyzed the data. J. S., P. H. and J. B. carried out the measurements related to the magnetic characterizations and analyzed the data. T. E. and L. H. recorded and analyzed the ^57^Mößbauer data. A. G. recorded and analyzed the single-crystal X-ray diffraction data for the title compounds. J. S. planned and carried out the (photo)degradation studies and analyzed the results. E. J. planned the quantum chemical investigations and analyzed the results. K. W. conceived, planned the research, and designed the title compounds. R. L. planned and guided the photophysical studies and drafted the manuscript together with C. J. C. J., J. S., M. D., E. J., K. W. and R. L. wrote the manuscript. All authors read and commented on the manuscript.

## Conflicts of interest

There are no conflicts to declare.

## Supplementary Material

SC-014-D3SC02806B-s001

SC-014-D3SC02806B-s002
