# Supplementary material for: Ferrous and ferric complexes with cyclometalating N-heterocyclic carbene ligands: a case of dual emission revisited
Source: Chem Sci. 2023 Aug 29;14(37):10129–39. doi: 10.1039/d3sc02806b (PMC10530338; doi:10.1039/d3sc02806b)
Supplement: SC-014-D3SC02806B-s001 [file SC-014-D3SC02806B-s001.pdf]

Electronic Supplementary Material (ESI) for Chemical Science.
This journal is © The Royal Society of Chemistry 2023

SUPPORTING INFORMATION

Ferrous and ferric complexes with cyclometalating *N*-heterocyclic carbene ligands: A case of dual emission revisited

Catherine Ellen Johnson, Jesper Schwarz, Mawuli Deegbey, Om Prakash, Kumkum Sharma, Ping Huang, Tore Ericsson, Lennart Häggström, Jesper Bendix, Arvind Kumar Gupta, Elena Jakubikova,* Kenneth Wärnmark,*,+ Reiner Lomoth*,+

+ = shared authorship

Contents

Methods ... **4**

Single crystal X-ray diffraction analysis ... 4

Determination of magnetic susceptibility using Evans' NMR method[5, 6] ... 4

Magnetic susceptibility and magnetization measurements ... 4

Mößbauer spectroscopy ... 4

Cyclic Voltammetry and Spectroelectrochemistry ... 5

Steady-State Spectroscopy measurements ... 5

Illumination measurements ... 6

Femtosecond transient absorption measurements ... 6

Global Analysis ... 6

Computational Details ... 6

Synthesis ... **8**

[1,1'-(1,3-phenylene)bis(3-methyl-1H-imidazol-3-ium)] dibromide, [HImP]Br_2 ... 8

[Bis(2,6-bis(3-methylimidazol-2-ylidene-1-yl))phenylene)iron(III)] hexafluorophosphate, [Fe^{III}($ImP)_2$]PF_6 ... 8

[Bis(2,6-bis(3-methylimidazol-2-ylidene-1-yl)phenylene)iron(II)], [Fe^{II}($ImP)_2$] ... 9

NMR spectra ... **10**

[1,1'-(1,3-phenylene)bis(3-methyl-1H-imidazol-3-ium)] dibromide ([HImP]Br_2) ... 10

Bis(2,6-bis(3-methylimidazol-1-ylidene)phenyl)iron(III) hexafluorophosphate ([Fe^{III}($ImP)_2$]PF_6) ... 15

Bis(2,6-bis(3-methylimidazol-1-ylidene)phenyl)iron(II) ([Fe^{II}($ImP)_2$]) ... 18

HRMS ... **22**

Single crystal X-ray diffraction analysis ... **23**

Magnetic susceptibility and magnetization measurements ... **30**

Mößbauer spectroscopy ... **31**

Stability experiments under illumination and in the dark ... **32**

Electrochemistry and Spectroelectrochemistry ... **34**

Absorption spectra ... **35**

TCSPC measurements of [Fe^{II}($ImP)_2$] ... **36**

Ligand precursor [HImP]($PF_6)_2$ photophysics ... **37**

Fs-TAS of [Fe^{III}($ImP)_2$]$^+$ ... **38**

Fs-TAS of [Fe^{II}($(ImP)_2$)] in tetrahydrofuran **39**

DFT calculations **42**

Optimized structures of Fe(II) and Fe(III) *42*

Ground state electronic structure of [$Fe^{III}$$(ImP)_2$]$^+$ *43*

Electronic absorption spectrum of [$Fe^{III}$$(ImP)_2$]$^+$ *44*

Potential energy curves of [$Fe^{III}$$(ImP)_2$]$^+$ *45*

Ground-state electronic structure of [$Fe^{II}$$(ImP)_2$] *46*

Electronic absorption spectrum of [$Fe^{II}$$(ImP)_2$] *48*

Potential energy curves of [$Fe^{II}$$(ImP)_2$] *49*

Stability studies of [$Fe^{II}$$(ImP)_2$] *50*

Potential energy curves of [$Fe^{II}$$(ImP)_2$($CH_3CN$)] *53*

References **62**

Methods

Single crystal X-ray diffraction analysis

All SC-XRD measurements were performed using graphite-monochromatized Mo Kα radiation (λ = 0.71073 Å) using the Agilent Xcalibur Sapphire3 diffractometer high-brilliance IμS radiation source. Data collections was performed at 150 K for $[Fe^{III}(ImP)_2]PF_6$ and $[Fe^{II}(ImP)_2]$. Absorption was corrected using multi-scan empirical absorption correction with spherical harmonics as implemented in the SCALE3 ABSPACK scaling algorithm.[1] The structure was solved by direct methods and refined by full-matrix least-squares techniques against F2 using all data (SHELXT, SHELXS).[2, 3] All non-hydrogen atoms were refined with anisotropic displacement parameters if not stated otherwise. Hydrogen atoms were constrained in geometric positions to their parent atoms using OLEX2 software.[4] The crystallographic data for $[Fe^{III}(ImP)_2]PF_6$ and $[Fe^{II}(ImP)_2]$ have also been deposited at the Cambridge Crystallographic Data Centre, under deposition numbers 2254083 and 2254082 respectively. Copies of these data can be obtained free of charge from www.ccdc.cam.ac.uk/structures.

Determination of magnetic susceptibility using Evans' NMR method[5, 6]

Solution state magnetic moments were determined by Evans' method using an NMR tube containing a solution of the paramagnetic complex and a capillary containing pure CD_3CN.

$$\chi_M = 3000\,\Delta\delta\,/\,(4\pi\,c) \qquad \mu_{eff} = (7.9933\,\chi_M\,T)^{1/2}$$

χM is the molar susceptibility, $\Delta\delta$ is the change in chemical shift between acetonitrile in the capillary and the NMR sample and c is the concentration in mol/L. μ_{eff} is the effective magnetic moment, and T is the temperature in K.

Magnetic susceptibility and magnetization measurements

The magnetic data were acquired on a Quantum-Design MPMS-XL SQUID magnetometer. Susceptibility data were acquired in a static field of 1.0 KOe. Magnetization data were obtained with selected fields from 1 to 50 KOe at T = 2 - 10 K in 1 K intervals. The polycrystalline samples were measured on a compacted powder sample in a polycarbonate capsule. Data were corrected empirically for TIP and the diamagnetic contribution to the sample moment from the sample holder and sample was corrected through background measurements and Pascal constants, respectively.

Mößbauer spectroscopy

Mößbauer measurements of $[Fe^{III}(ImP)_2]PF_6$ were carried out in an Oxford Instrument flow cryostat at 85 K and at 295 K using a ^{57}CoRh source held at room temperature. The studied powder material was mixed with inert BN, pressed, and formed as pastille absorber with a concentration of about 30 mg/cm^2

of studied substances. Calibration spectra were recorded from a natural iron metal foil held at 295 K. The resulting spectra were analyzed using a least square Mößbauer fitting program. Mößbauer measurements of [Fe^{II}($ImP)_2$] were unsuccessful because of the rapid oxidation to [Fe^{III}($ImP)_2$]$^+$ in air.

Cyclic Voltammetry and Spectroelectrochemistry

All electrochemical experiments were performed in acetonitrile (spectroscopic grade Uvasol®, ≥99.9%, Merck; dried over 3Å molecular sieves activated at 300 °C for 15 hours) with 0.1 M tetrabutylammonium hexafluorophosphate (electrochemical grade, Sigma Aldrich; dried at 80 °C under vacuum) and purged with solvent-saturated argon. The samples were prepared with a concentration of 1 mM.

Cyclic voltammetry (CV) measurements were carried out in a three-electrode electrochemical cell, using an AUTOLAB potentiostat (PGSTAT302) controlled with GPES software (Version 4.9). The working electrode was a freshly-polished (with Buehler alumina paste) glassy carbon electrode (CH Instruments, 1 mm diameter); the reference electrode was a non-aqueous Ag/$AgNO_3$ (CH Instruments; 10 mM of $AgNO_3$ dissolved in dried acetonitrile; 0.08 V vs. ferrocene) and a Pt wire in a separate compartment was used as counter electrode.

UV-Vis spectroelectrochemistry measurements were performed in a diode array spectrophotometer (Agilent 8453) with an optically transparent thin-layer cell (1 mm optical path length) equipped with a platinum mesh working electrode and the same reference and counter electrodes used for voltammetry. Time-resolved spectra were recorded during controlled potential electrolysis using an AUTOLAB potentiostat (PGSTAT302).

Steady-State Spectroscopy measurements

Samples were prepared in either acetonitrile or tetrahydrofuran (both spectroscopic grade Uvasol®, ≥99.9%, Merck). Samples of [Fe^{III}($ImP)_2$]$^+$ were prepared under standard conditions, while those for [Fe^{II}($ImP)_2$] were prepared with deaerated solvents in an argon-filled glove box due to its high oxygen sensitivity. UV-Vis absorption spectra were recorded on Varian Cary 50 or Cary 5000 spectrophotometers.

Steady-state emission and excitation measurements were performed on an FS5 Spectrofluorometer (Edinburgh Instruments) with 5 nm spectral resolution. Samples were prepared in 10 mm cuvettes and measurements were performed with right-angle geometry. Emission and excitation spectra were all background subtracted and corrected for detector response.

Time-Correlated Single Photon Counting measurements were completed with the same instrument with picosecond pulsed diode lasers (EPL Series) with excitation wavelengths of 340 and 375 nm. The fluorescence lifetime measurements were fitted with the instrument response function (IRF) using the in-built reconvolution fitting in the Fluoracle® software.

Illumination measurements

The photoreaction of [$Fe^{II}(ImP)_2$] was studied by absorption and emission spectroscopy. Initial UV-Vis absorption and emission spectra of an oxygen free solution of [$Fe^{II}(ImP)_2$] in a gas tight cuvette were taken, with as little exposure to light in between as possible. The cuvette was then exposed to (i) ambient light and (ii) a white light LED lamp (ZENARO, SL-PAR38/B/P17/20/E50/TD/27/HAC, 50-60 Hz, ~ 50 $mWcm^{-2}$). UV-Vis absorption and emission spectra were collected immediately after illumination (Shown in Fig. 3b).

To track the photodecomposition over time, solutions of [$Fe^{II}(ImP)_2$] were prepared in a glovebox under nitrogen and transferred to an air-tight quartz cuvette (10 mm, Hellma Analytics QS). The cuvette was irradiated using blue LEDs (λ = 450 nm), and the absorption spectrum was measured at regular time intervals using an Agilent Cary 60 spectrometer (Shown in Fig. 5).

Femtosecond transient absorption measurements

Femtosecond transient absorption spectroscopy (fs-TAS) measurements were performed probing in the UV-Vis region on a Newport TAS system with a Coherent Libra Ti:sapphire amplifier (800 nm, 1.5 mJ, 3 kHz repetition rate, FWHM 45 fs). Different excitation wavelengths were generated by optical parametric amplifiers (TOPAS-Prime and NIRUVVIS, Light Conversion) and then focused and centered on the 1 mm cuvette with corresponding pump powers. The white light supercontinuum probe light was generated using a CaF_2 crystal (Crystran) and was detected by a silicon diode array (Newport custom made). A mechanical chopper blocked every other pump pulse, and the transient absorption at each time point was calculated for an average of 1000 ms chopped/un-chopped pulse pairs. To record the transient absorption spectra at different time points, an optical delay line was used to scan the delay of the probe beam relative to the pump beam (maximum –5 ps to 8 ns). A total of 8 scans were collected and averaged for each sample. Prior to analysis, the data was corrected for the spectral chirp using Surface Xplorer v4, where single wavelength fits were also performed.

Global Analysis

All fs-TAS data were further analyzed with Global Analysis using the software Glotaran (Version 1. 5. 1), which is a Java-based graphical user interface to the R package TIMP.[7] All data were fitted with necessary number of components and the decay-associated spectra (DAS) were exported from the software and analyzed in Origin.

Computational Details

Density functional theory (DFT) calculations utilizing the B3LYP functional[8] with Grimme's D2 dispersion correction[9] were employed to optimize structure of the singlet ground state (^{1}GS), as well

as the triplet and quintet metal-centered states (^{3,5}MC) and triplet metal-to-ligand charge transfer (^{3}MLCT) states of $[Fe^{II}(ImP)_2]^0$ complex, and the doublet (^{2}GS), quartet (^{4}MC) and sextet (^{6}MC) states of $[Fe^{III}(ImP)_2]^+$ complex. The 6-311G* basis set was used for all atoms (H, C, N)[10, 11] except for Fe, where the SDD basis sets and its accompanying pseudopotential[12] were employed in all calculations. Solvent effects (acetonitrile) were included in all the calculations via the polarizable continuum model (PCM).[13] Vibrational frequency analysis was performed to ensure that the optimized structures correspond to minima on their respective potential energy surfaces. Fragment molecular orbital analysis (FMOA) based on the Mulliken population analysis was carried out with the AOMix software[14, 15] in order to obtain the percent contributions of each fragment. A two-fragment scheme was employed: Fragment 1 (Fe), Fragment 2 (ligands). All calculations were carried out using the Gaussian 16, Revision A.03 software package.[16]

Time-dependent DFT (TD-DFT)[17] calculations at the same level of theory as the structure optimizations were employed to simulate the UV-Visible absorption spectra in acetonitrile. The stick spectra were broadened using Lorentzian functions with a half-width-at-half-maximum (HWHM) of 0.12 eV. The hole and electron density pairs of each transition responsible for the absorption spectra were characterized by means of natural transition orbitals (NTO).[18]

Potential energy curves (PECs) versus the average Fe-C bonds were constructed for the various spin states of Fe(II) and Fe(III) complexes to understand the excited states dynamics. DFT single point energy calculations were carried out on the initial fully optimized minima, to obtain their corresponding ^{2}GS, ^{4,6}MC states for Fe(III), and ^{1}GS, ^{3,5}MC states for Fe(II). Tamm–Dancoff approximation (TDA)[19] was then employed to calculate the vertical excitations on the produced reference wavefunctions: ^{2}GS for Fe(III) and ^{1}GS for Fe(II), in order to obtain the first ^{2}LMCT and ^{2}MLCT states in Fe(III), and the first ^{1}MLCT, ^{3}MC, and ^{3}MLCT states in Fe(II). TDA rather than TD-DFT was used for these calculations due to the triplet instabilities in TD-DFT calculations that occurred at ^{3,5}MC geometries.[20]

PECs from a relaxed potential energy surface scan along the partially detached Fe-C bond (shown in red in Fig. S29) were also carried out to compare the reaction pathway of the ligand detachment for the ^{1}GS and ^{3}MC states of the van der Waals solvent coordinated complex $[Fe(ImP)_2(CH_3CN)]$. The Fe-C bonds were constrained to values between 4.6 Å and 2.0 Å with 0.1 Å step, starting from the conformation in which the Fe-C bonds are set to 4.6 Å.

Synthesis

All reactions were carried out using oven-dried glassware under an atmosphere of nitrogen. All solvents for synthesis were used as received and were of synthesis grade, unless otherwise stated. Tetrahydrofuran (THF) was dried over Na/benzophenone and subsequently distilled under nitrogen before use. Acetonitrile was dried over molecular sieves and degassed by freeze-pump-thaw cycles. Anhydrous dichloromethane was obtained from a Braun SPS-800 system. Reagents were obtained from commercially available sources and used as received unless stated otherwise. Commercially available starting materials were purchased from Acros, Merck, or Fischer Scientific.

[1,1'-(1,3-phenylene)bis(3-methyl-1*H*-imidazol-3-ium)] dibromide, [HImP]Br_2

[1,1'-(1,3-phenylene)bis(3-methyl-1*H*-imidazol-3-ium)] hexafluorophosphate[21] (2.380 g, 4.489 mmol) was dissolved in a minimum amount of dry acetone (10 mL) and precipitated by adding tetrabutylammonium bromide (1.620 g, 5.027 mmol). The resulting white precipitate was filtered and washed with dry acetone (3 × 10 mL) followed by drying under vacuum affording [HImP]Br_2 as a white solid (1.696 g, 94%).

R_f = 0.49 (acetonitrile:water 9:1, UV-active)

^{1}H NMR (400 MHz, DMSO-d_6) δ (ppm) = 10.06 (t, *J* = 1.7 Hz, 2H), 8.48 (t, *J* = 1.9 Hz, 2H), 8.41 (t, *J* = 2.1 Hz, 1H), 8.09 – 7.92 (m, 5H), 4.00 (s, 6H).

^{13}C NMR (101 MHz, DMSO-d_6) δ (ppm) = 136.4, 135.8, 132.1, 124.7, 122.4, 120.9, 115.5, 36.4.

HRMS (ESI-TOF) calc'd for $[C_{14}H_{15}N_4]^+$ 239.1297; found 239.1298.

Elemental analysis (% calc'd, % found for $C_{14}H_{16}N_4Br_2 \bullet 1.4\ H_2O$): C (39.53, 39.52), H (4.46, 4.40), N (13.17, 13.18).

[Bis(2,6-bis(3-methylimidazol-2-ylidene-1-yl))phenylene)iron(III)] hexafluorophosphate, $[Fe^{III}(ImP)_2]PF_6$

[1,1'-(1,3-Phenylene)bis(3-methyl-1-imidazolium)] dibromide (803 mg, 2.01 mmol) and tetrakis(dimethylamido)zirconium (642 mg, 2.40 mmol) were charged into a Schlenk flask in a glovebox under nitrogen. Dry THF (10 mL) was added to the Schlenk flask, and the yellow suspension was stirred for 2 h under nitrogen at room temperature. Iron(II)bromide (240 mg, 1.10 mmol) was sonicated in dry THF (24 mL) under nitrogen to give a brown solution that was added in one portion to the reaction. The reaction turned into a brown suspension which was stirred in the dark under nitrogen for another 16 h. The brown suspension was then subjected to air, upon which it immediately turned blue. To the suspension was added methanol (1 mL) and the reaction mixture was stirred for another 3 h, after

which it was filtered through a glass frit filter (porosity 3). The filter was washed with acetonitrile until the filtrate was colourless, and the combined filtrates were concentrated in vacuo to give a dark blue solid. The solid was redissolved in methanol (30 mL), filtered and reprecipitated by pouring it into an aqueous solution of ammonium hexafluorophosphate (855 mg in 50 mL water). The dark blue precipitate was filtered off, redissolved in dichloromethane and reprecipitated by the addition of diethyl ether. The resulting dark blue product was purified by size-exclusion chromatography on BioBeads S-X1 (3 × 120 cm) using acetonitrile/toluene (50/50) as eluent. The resulting product was recrystallized from acetonitrile/diethyl ether to give $[Fe^{III}(ImP)_2]PF_6$ as dark blue crystals (171 mg, 26%).

R_f = 0.31 (toluene:acetonitrile 3:1)

^{1}H NMR (400 MHz, CD_3CN) δ (ppm) = 24.96 (s, 4H), 9.75 (s, 12H), 2.82 (s, 4H), -2.52 (s, 4H), -36.27 (s, 2H).

^{13}C NMR (151 MHz, CD_3CN) δ (ppm) = 520.5, 474.4, 87.4, 77.3, 2.6, -202.2. The resonances corresponding to the two different carbon atoms attached to iron could not be found despite several attempts varying the relaxation time.

Magnetic moment (Evans' method, acetonitrile, n = 3): 2.09 ±0.04 μ_B

HRMS (ESI-TOF) calc'd for $[C_{28}H_{26}N_8Fe]^+$ 530.1630; found 530.1631.

Elemental analysis (% calc'd, % found for $C_{28}H_{26}F_6FeN_8P$): C (49.80, 49.72), H (3.88, 3.90), N (16.59, 16.54).

[Bis(2,6-bis(3-methylimidazol-2-ylidene-1-yl)phenylene)iron(II)], $[Fe^{II}(ImP)_2]$

To a suspension of [bis(2,6-bis(3-methylimidazol-1-ylidene)phenyl)iron(III)] hexafluorophosphate (82.9 mg, 0.123 mmol) in dry THF (5 mL) was added lithium aluminium hydride (1 M in THF, 0.6 mL, 5 equiv.) under nitrogen at room temperature. The dark blue suspension turned into a clear orange solution. The solvent was evaporated with a stream of nitrogen gas, and the resulting orange solid was redissolved in dichloromethane (8 mL), filtered through a Schlenk filter (porosity 3) and the dichloromethane was evaporated with a stream of nitrogen gas to give $[Fe^{II}(ImP)_2]$ as an orange solid (35 mg, 54%).

^{1}H NMR (400 MHz, THF-d_8) δ (ppm) = 7.50 (d, J = 2.0 Hz, 2H), 7.09 (d, J = 7.4 Hz, 2H), 6.91 (t, J = 7.4 Hz, 1H), 6.45 (d, J = 2.0 Hz, 2H), 2.20 (s, 6H).

^{13}C NMR (101 MHz, THF-d_8) δ (ppm) = 214.2, 202.6, 149.2, 121.9, 116.5, 112.7, 103.2, 35.6.

Elemental analysis (% calc'd, % found for $C_{28}H_{26}FeN_8$): C (63.40, 63.17), H (4.94, 4.93), N (21.13, 21.07).

NMR spectra

NMR spectra were recorded at ambient temperature on a BrukerAvance II 400 MHz NMR spectrometer (400/101 MHz $^1H/^{13}C$) or a Bruker Avance Neo 600 MHz spectrometer (600/151 MHz $^1H/^{13}C$), equipped with a QCI CryoProbe. The initial ^{13}C-spectra of $[Fe^{III}(ImP)_2]PF_6$ showed splitting of the peaks at 474 and –202 ppm. However, the splitting disappeared when the carrier frequency for the ^1H-decoupling was shifted to get proper decoupling. The 2D ^{13}C-HSQC experiment of $[Fe^{III}(ImP)_2]PF_6$ was recorded in subsections, using different carrier frequencies for ^1H and ^{13}C in order to get proper excitation profile and ^{13}C-decoupling. Chemical shifts (d) for ^1H and ^{13}C NMR spectra are reported in parts per million (ppm), relative to the residual solvent peak of the respective NMR solvent: CD_3CN (δ_H = 1.94 and δ_C = 118.26 ppm), DMSO-d_6 (δ_H = 2.50 and δ_C = 39.52 ppm), and THF-d_8 (δ_H = 3.58 and δ_C = 67.21 ppm).[22] Coupling constants (J) are given in Hertz (Hz), with the multiplicities being denoted as follows: singlet (s), doublet (d), triplet (t), quartet(q), quintet(qi), multiplet (m), broad (br). NMR spectra for ^{13}C were recorded with decoupling of ^1H. For the assignment of spectra, see below.

[1,1'-(1,3-phenylene)bis(3-methyl-1*H*-imidazol-3-ium)] dibromide ([HImP]Br_2)

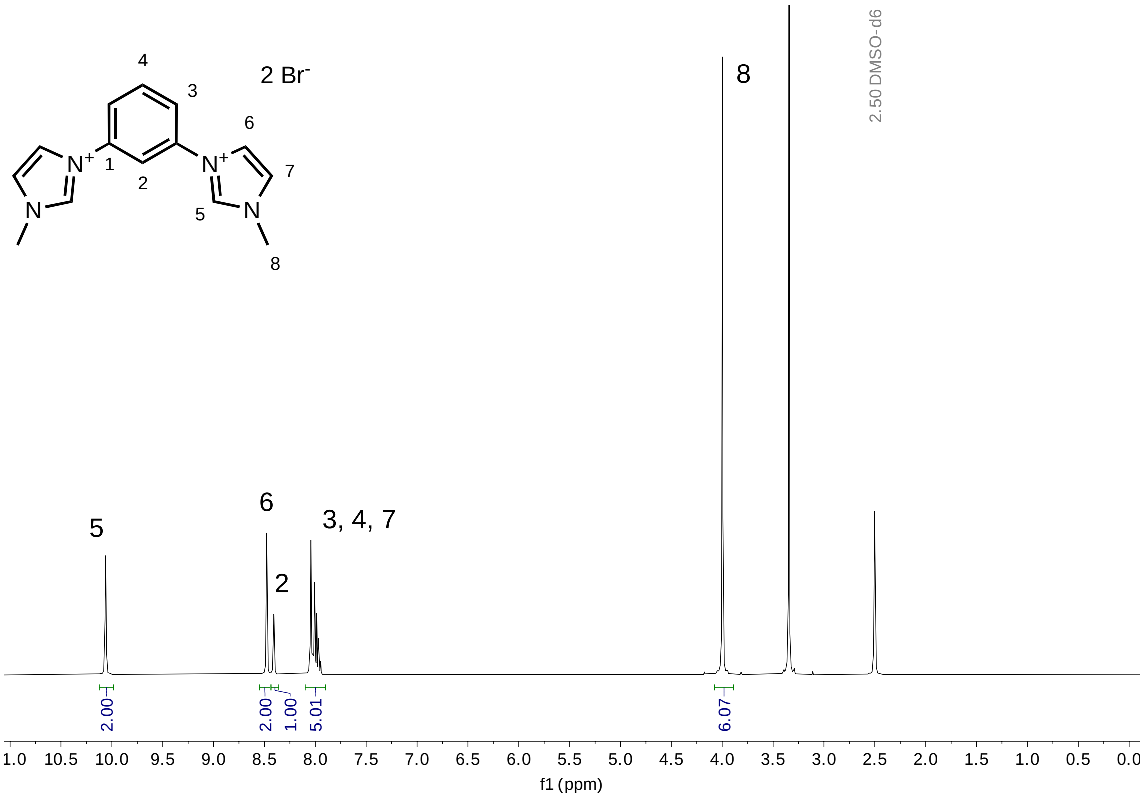

^1H-NMR (400 MHz, DMSO-d_6) of [HImP]Br_2

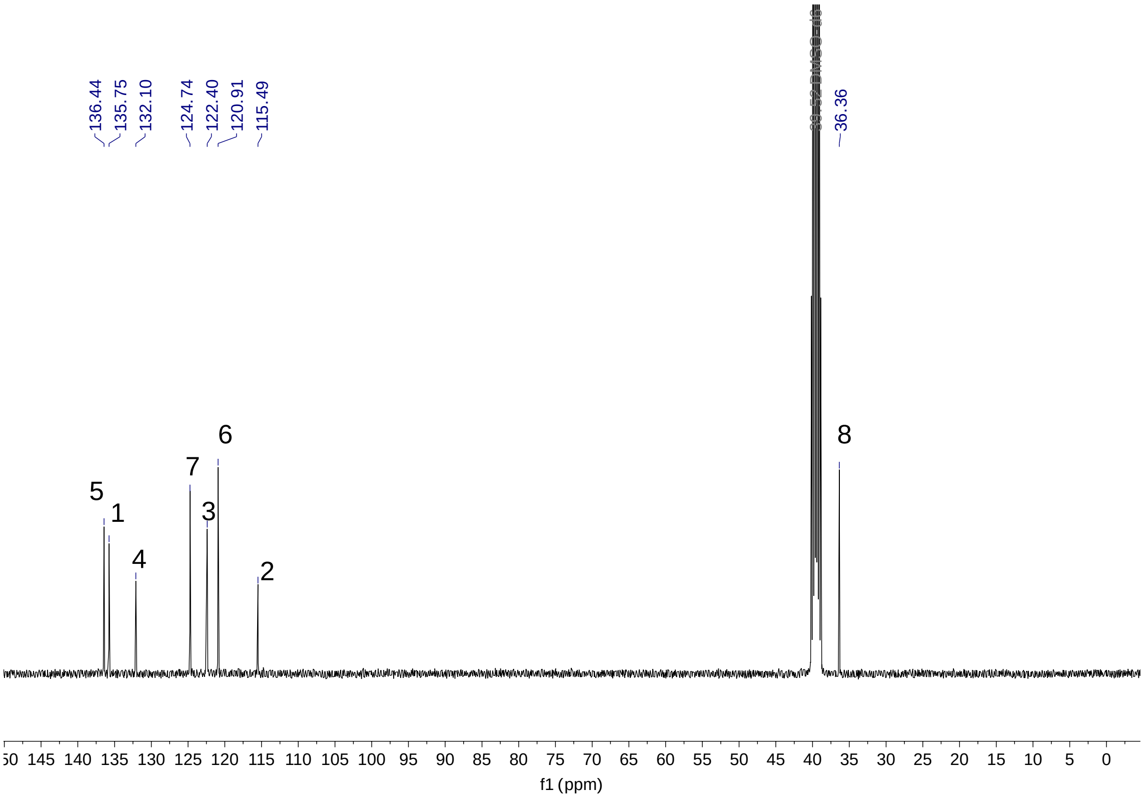

^{13}C-NMR (101 MHz, DMSO-d_6) of [HImP]Br_2

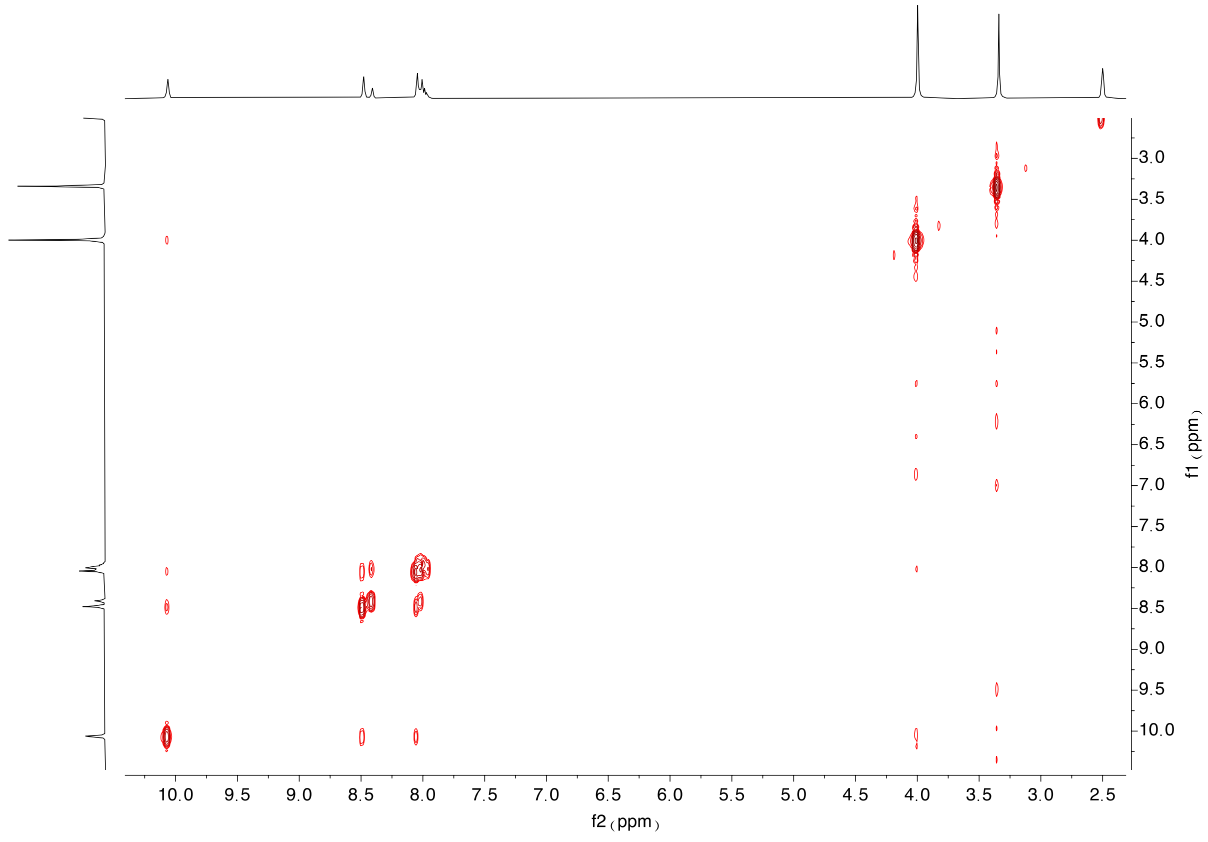

COSY (400 MHz, DMSO-d_6) of [HImP]Br_2

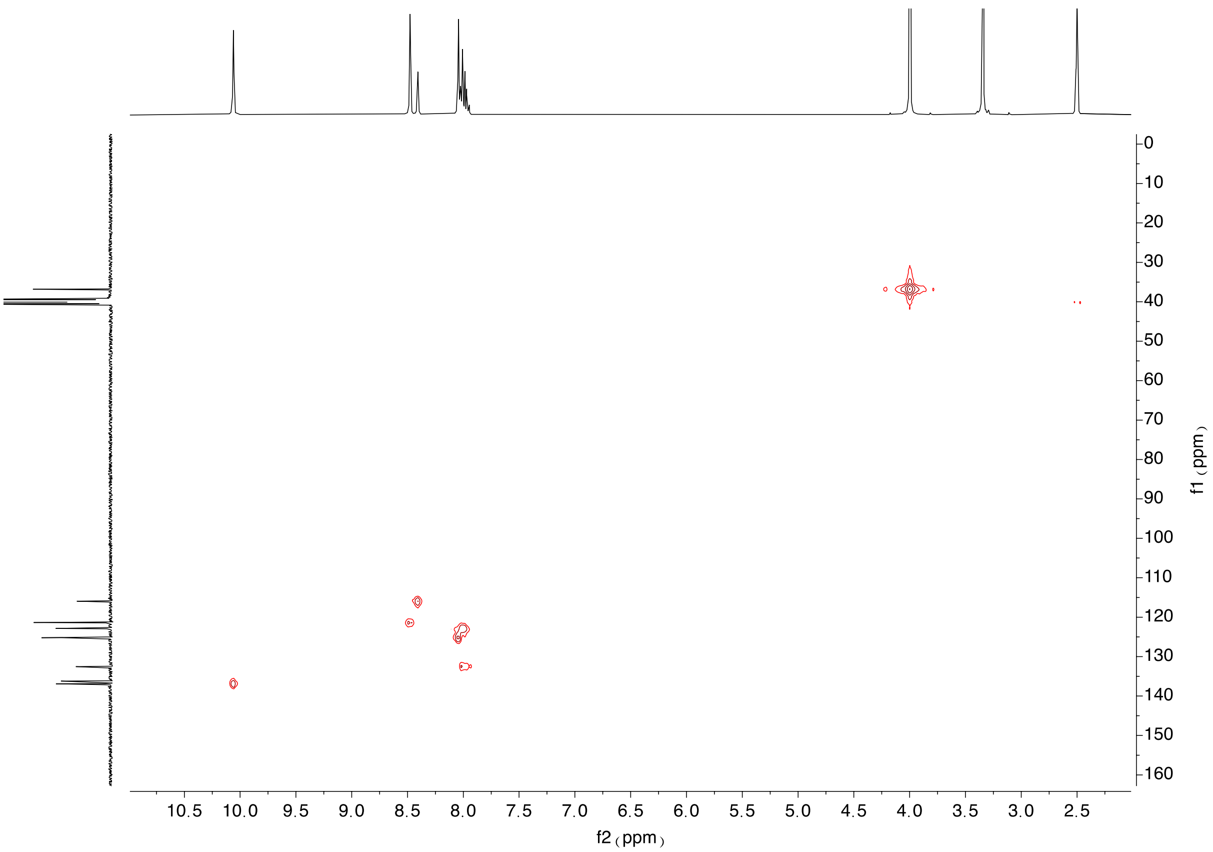

HMQC (400 MHz, DMSO-d_6) of [HImP]Br_2

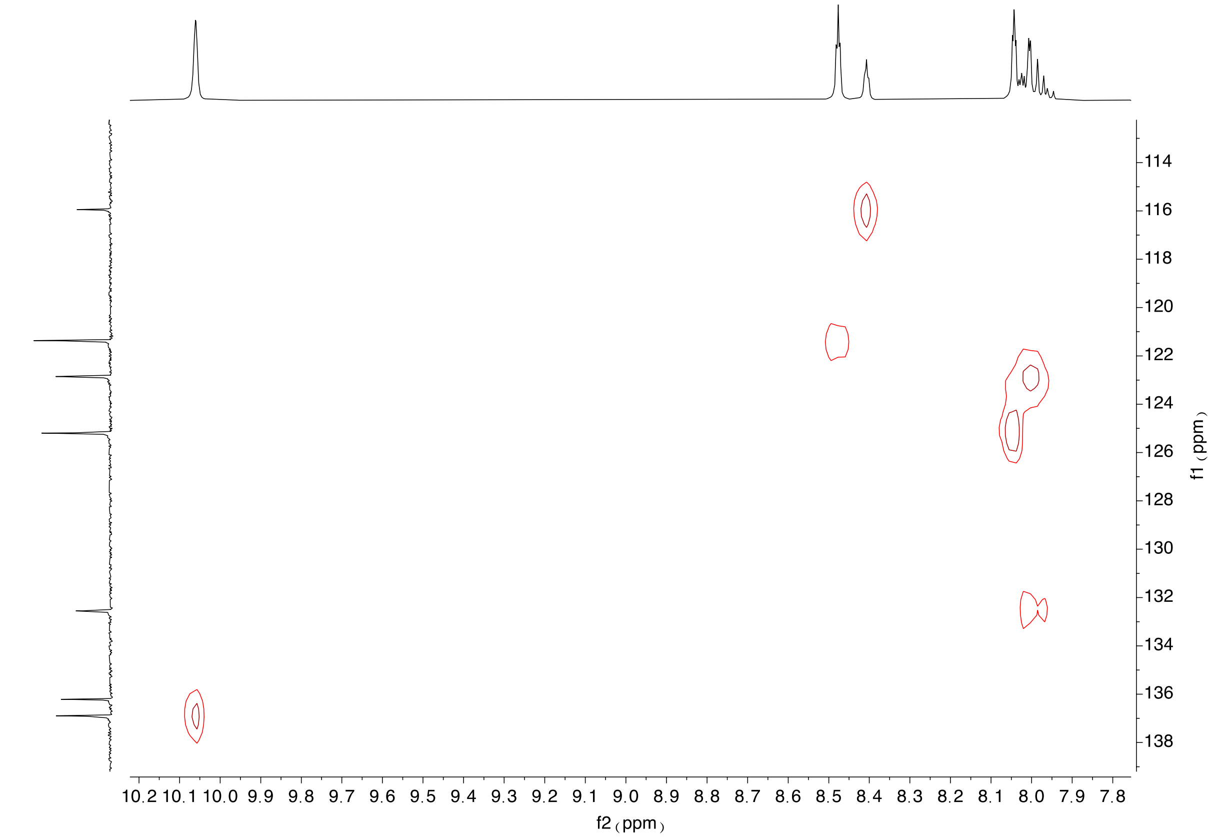

Expansion HMQC (400 MHz, DMSO-d_6) of [HImP]Br_2

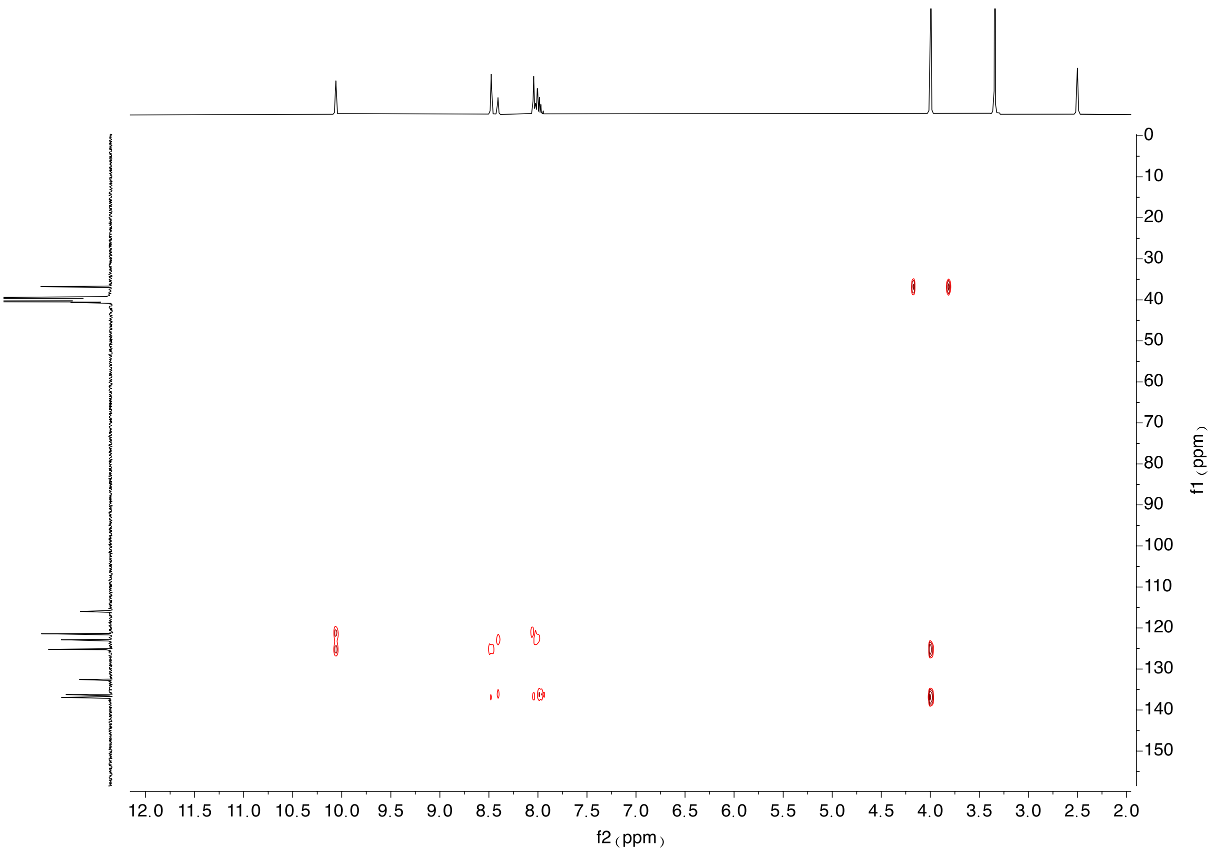

HMBC (400 MHz, DMSO-d$_6$) of [HImP]Br$_2$

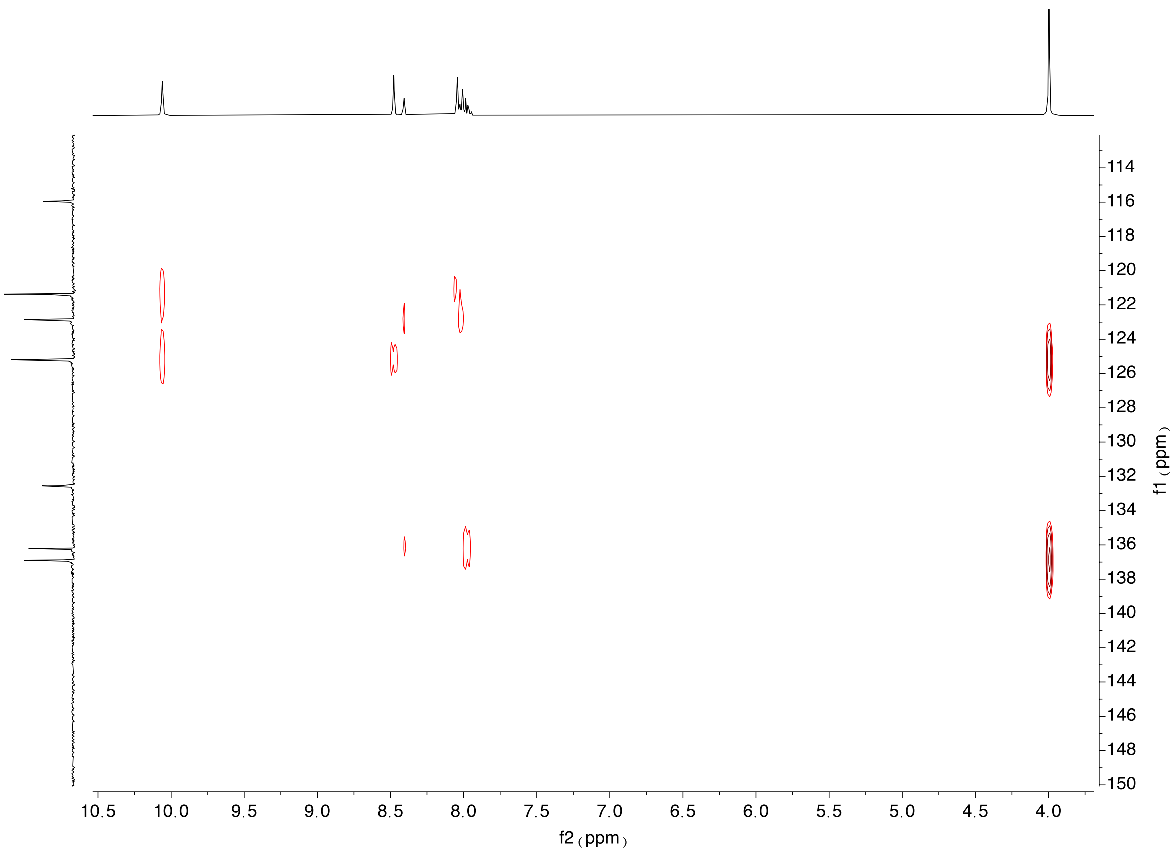

Expansion HMBC (400 MHz, DMSO-d$_6$) of [HImP]Br$_2$

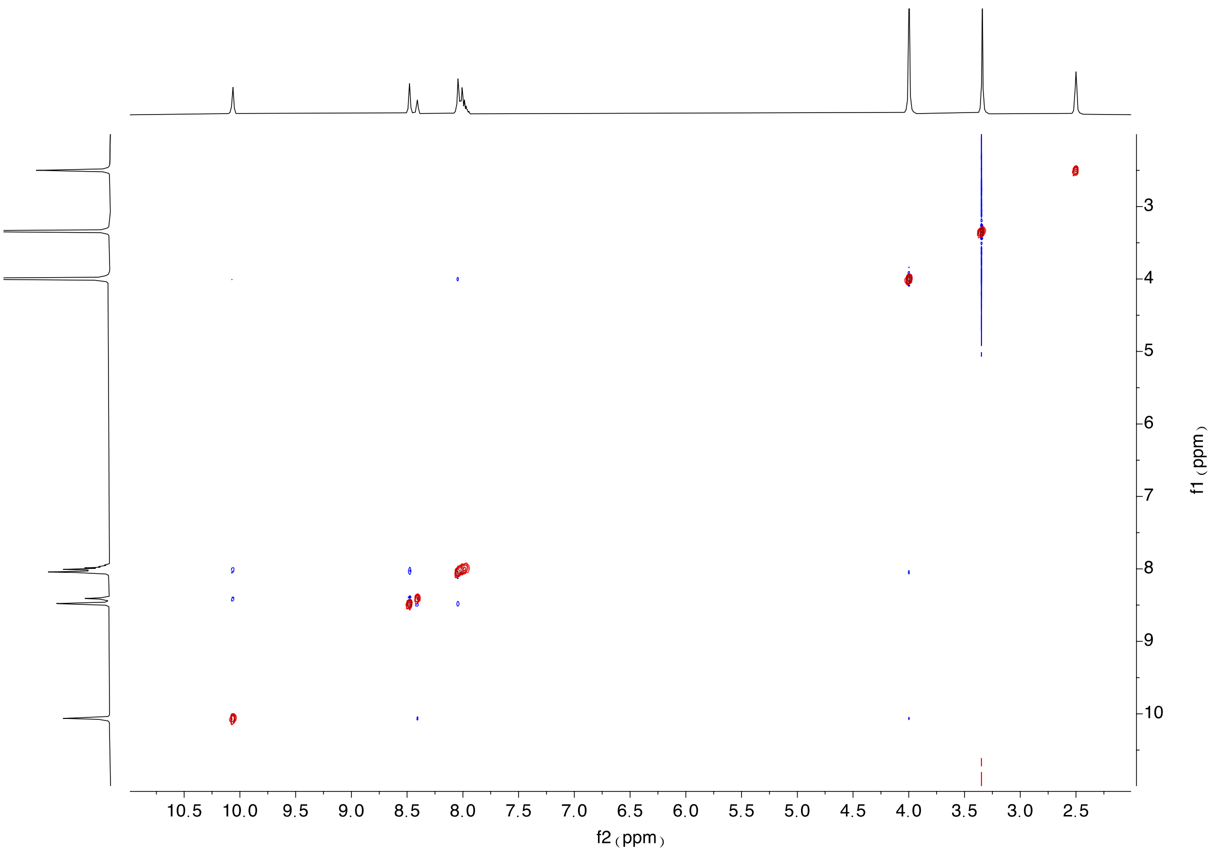

NOESY (400 MHz, DMSO-d_6) of [HImP]Br_2

Bis(2,6-bis(3-methylimidazol-1-ylidene)phenyl)iron(III) hexafluorophosphate ([$Fe^{III}(ImP)_2$]PF_6)

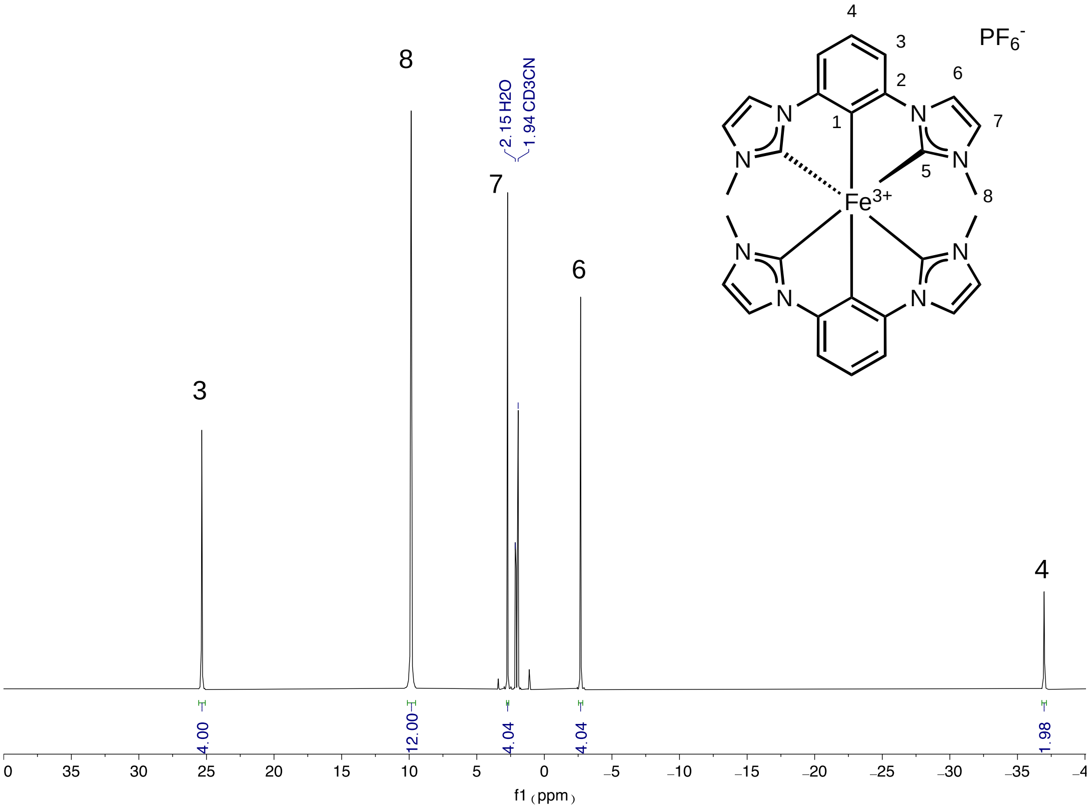

^{1}H-NMR (400 MHz, CD_3CN) of [$Fe^{III}(ImP)_2$]PF_6

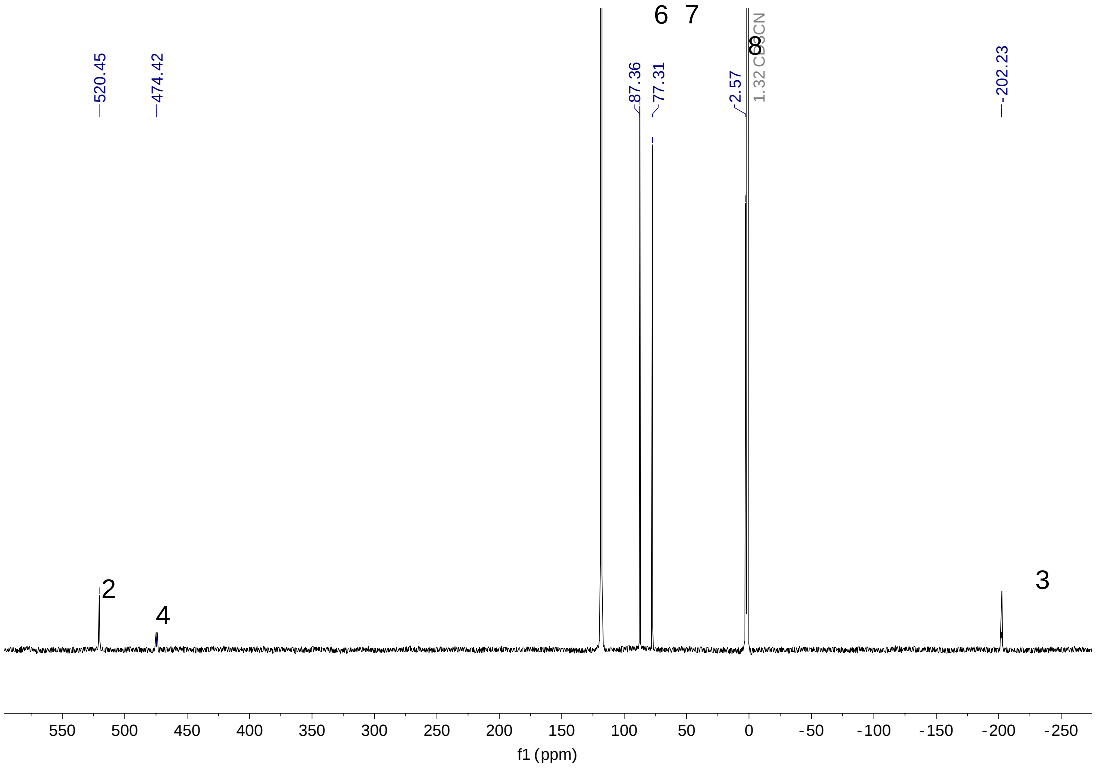

^{13}C NMR (151 MHz, CD_3CN) of [$Fe^{III}(ImP)_2$]PF_6

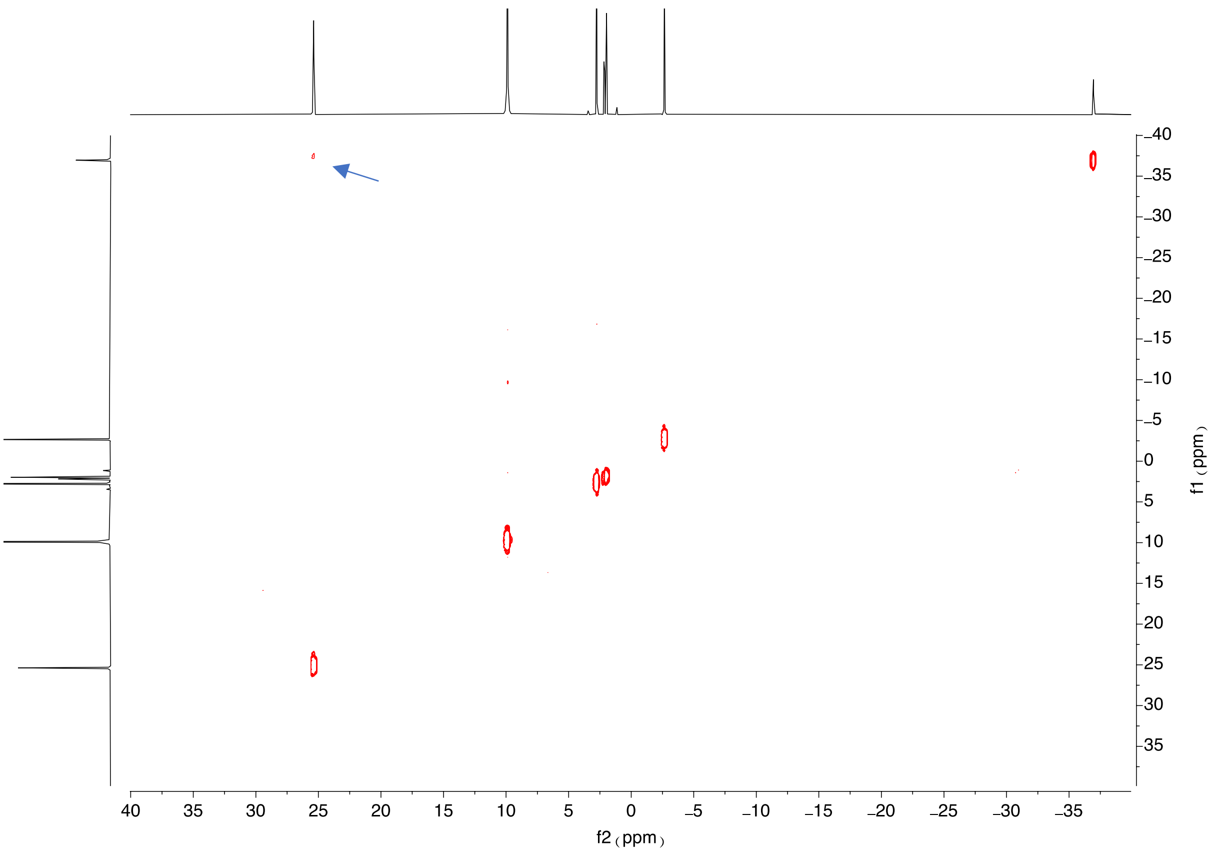

COSY (400 MHz, CD_3CN) of $[Fe^{III}(ImP)_2]PF_6$

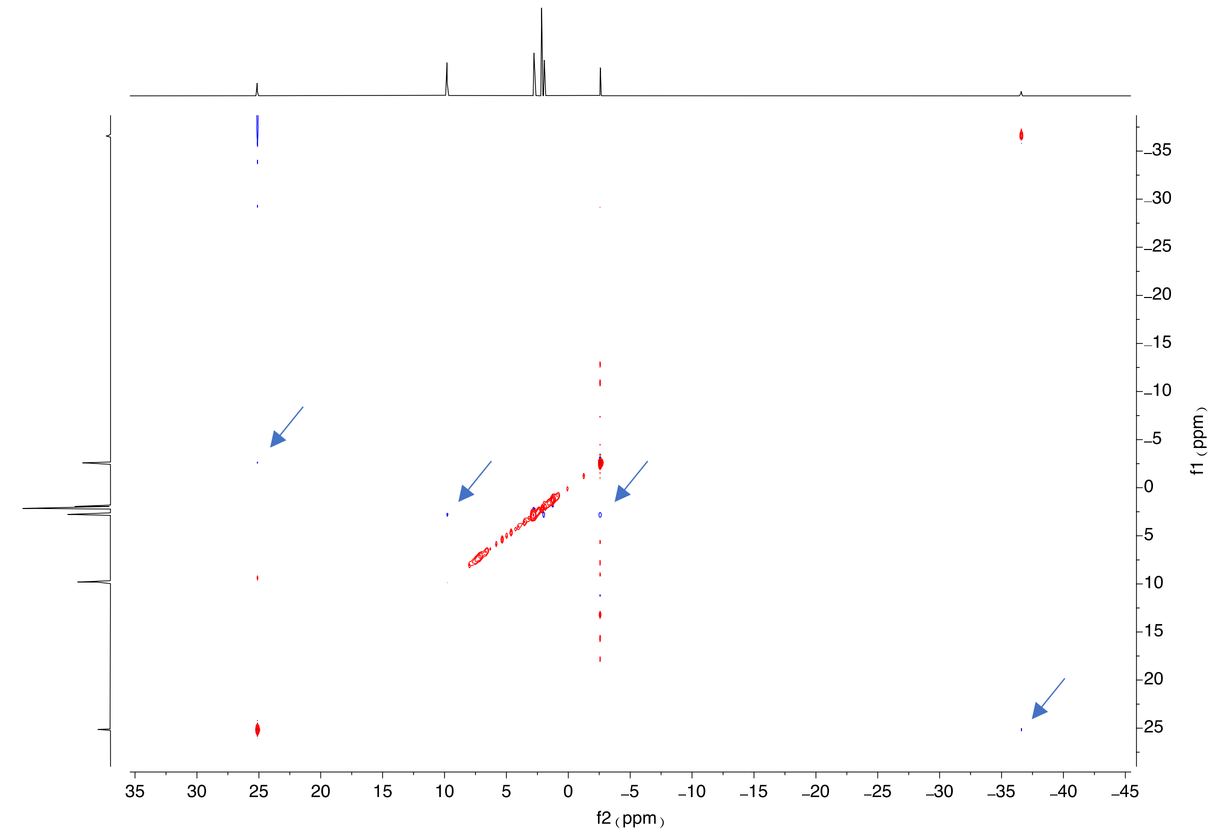

NOESY (600 MHz, CD_3CN) of $[Fe^{III}(ImP)_2]PF_6$

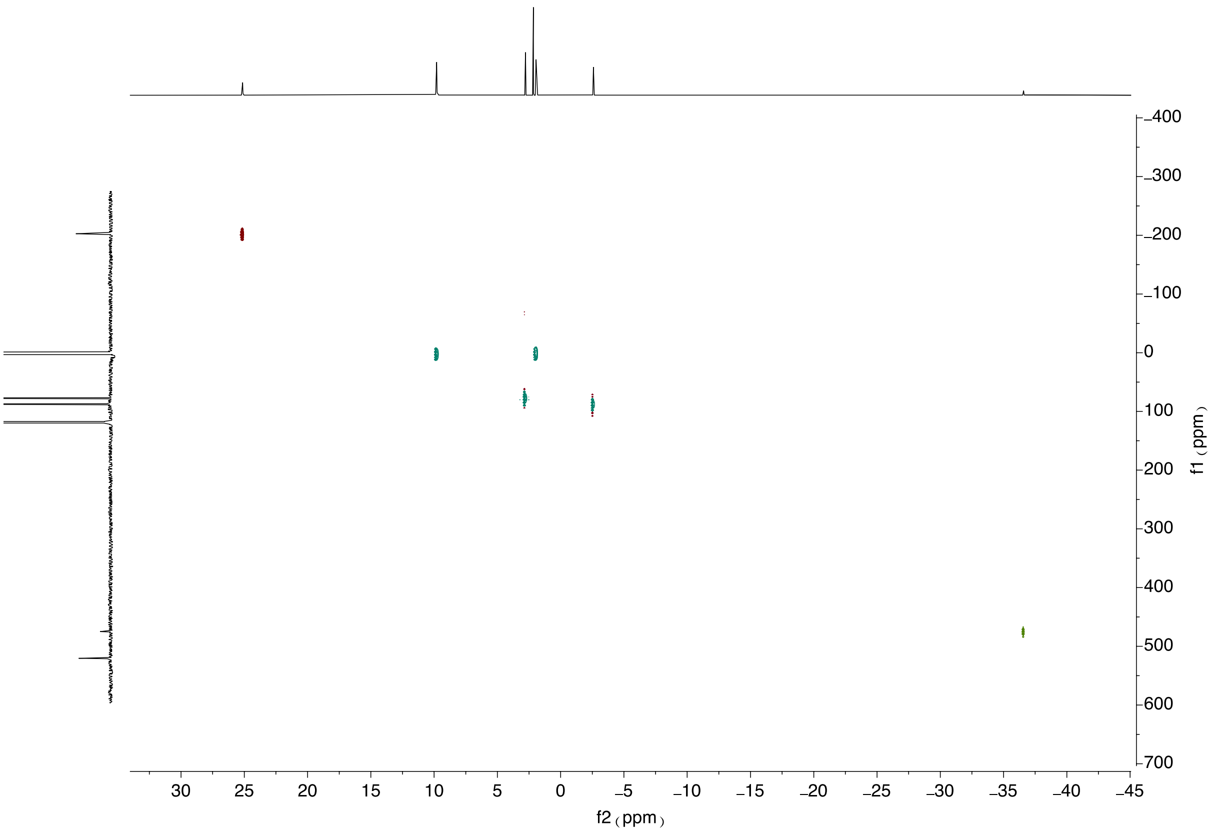

HSQC (600 MHz, CD_3CN) of $[Fe^{III}(ImP)_2]PF_6$

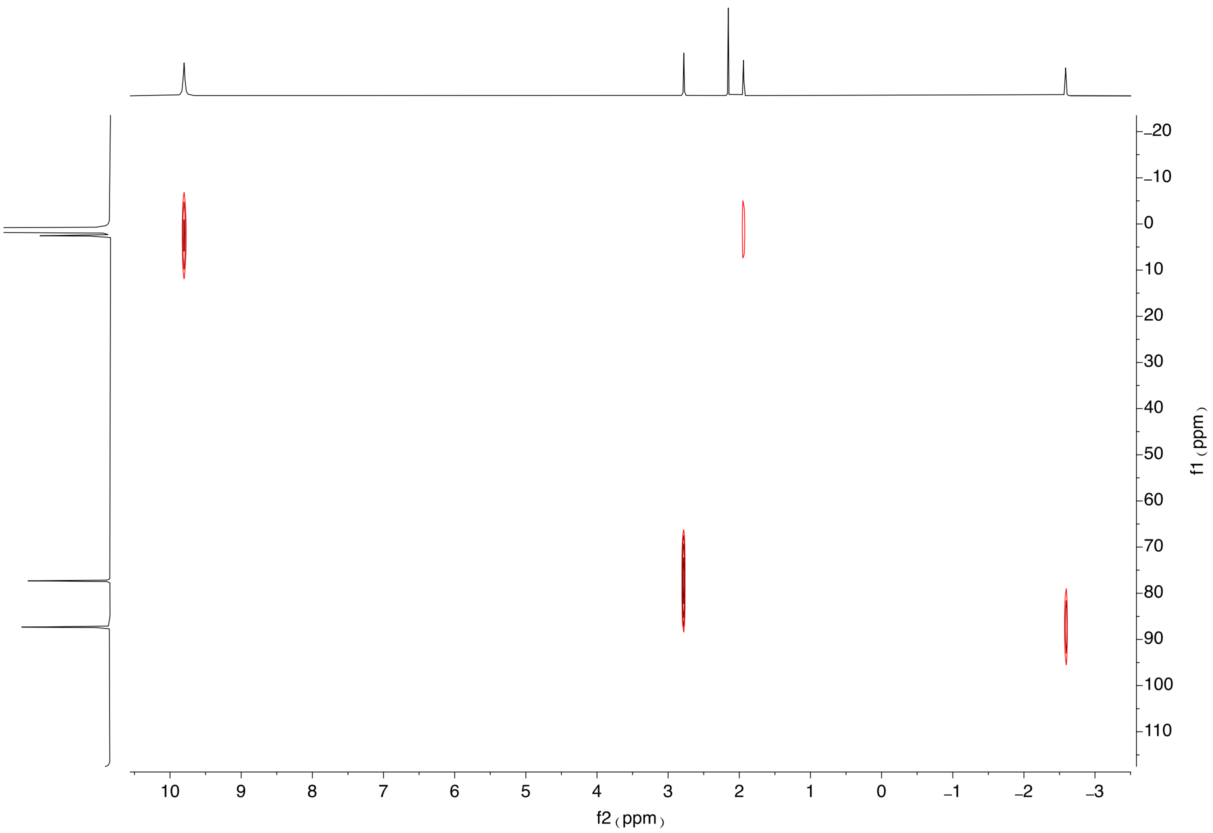

Expansion of HSQC (600 MHz, CD_3CN) of $[Fe^{III}(ImP)_2]PF_6$

Bis(2,6-bis(3-methylimidazol-1-ylidene)phenyl)iron(II) ([$Fe^{II}(ImP)_2$])

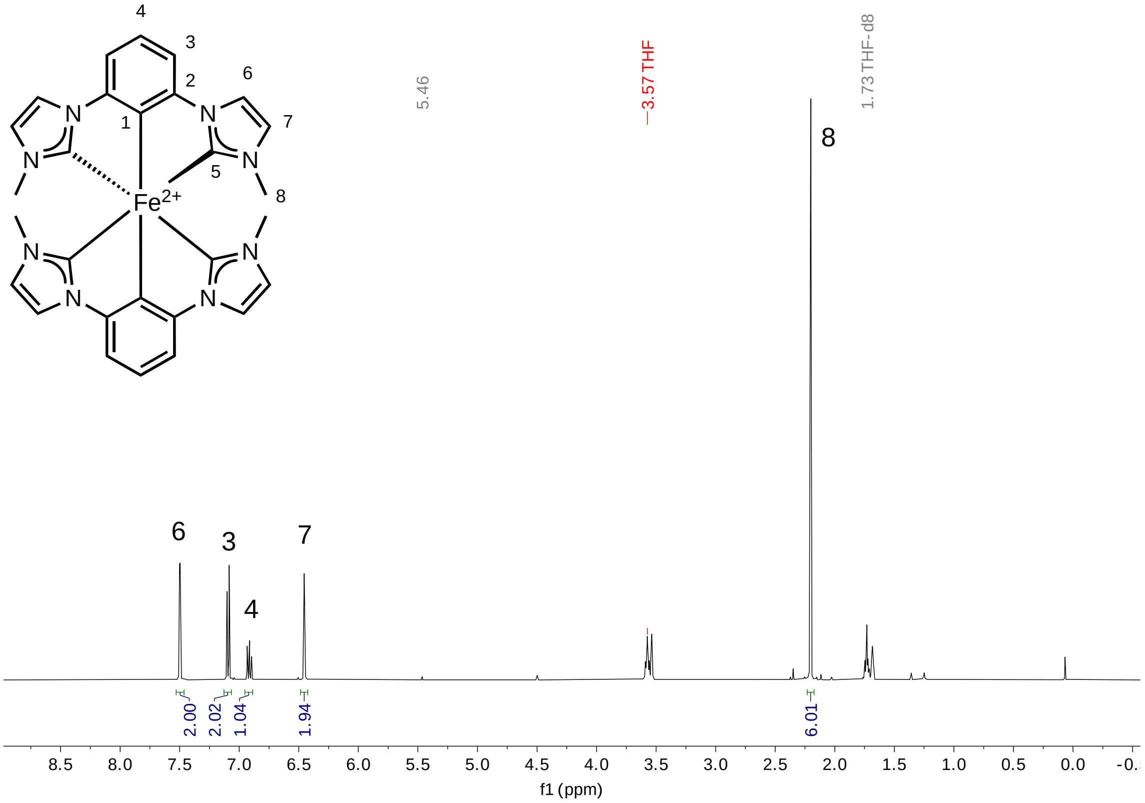

^{1}H NMR (400 MHz, THF-d_8) of [$Fe^{II}(ImP)_2$]

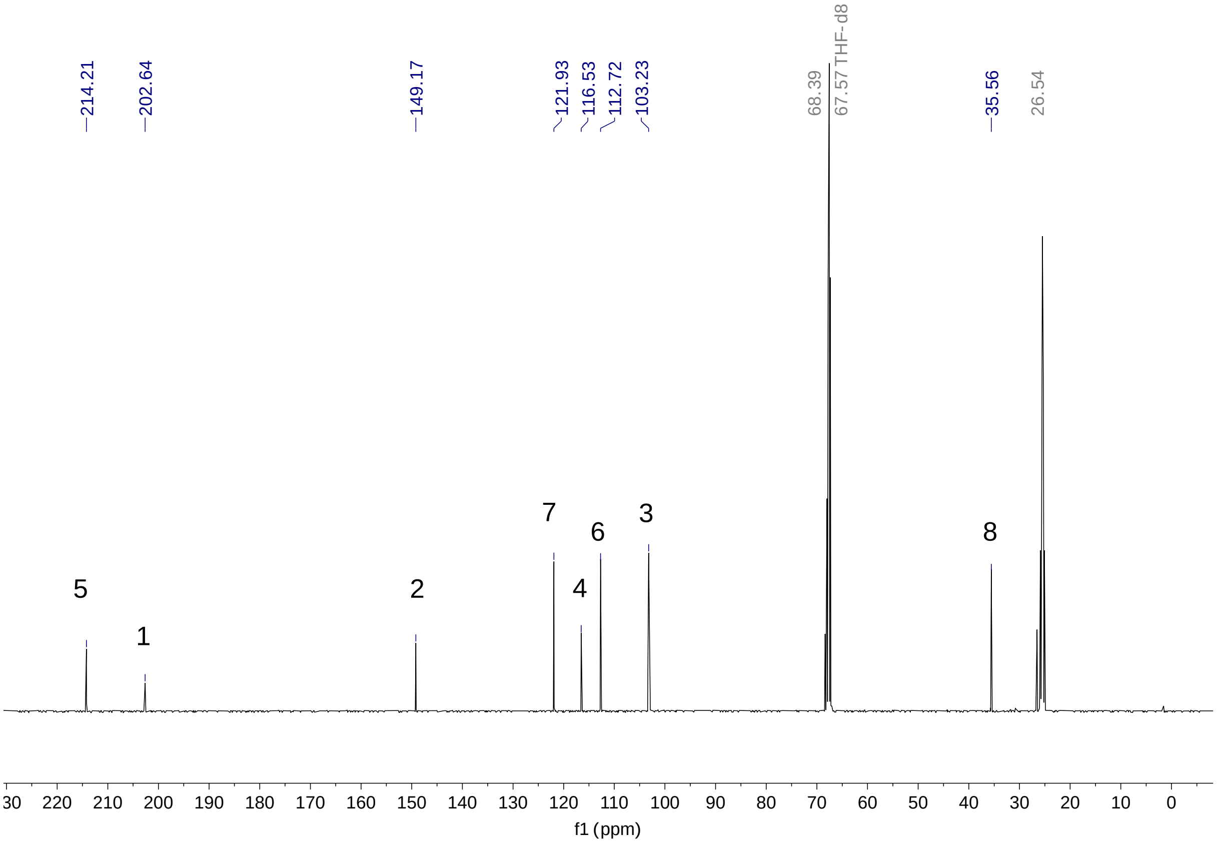

^{13}C NMR (101 MHz, THF-d_8) of [$Fe^{II}(ImP)_2$]

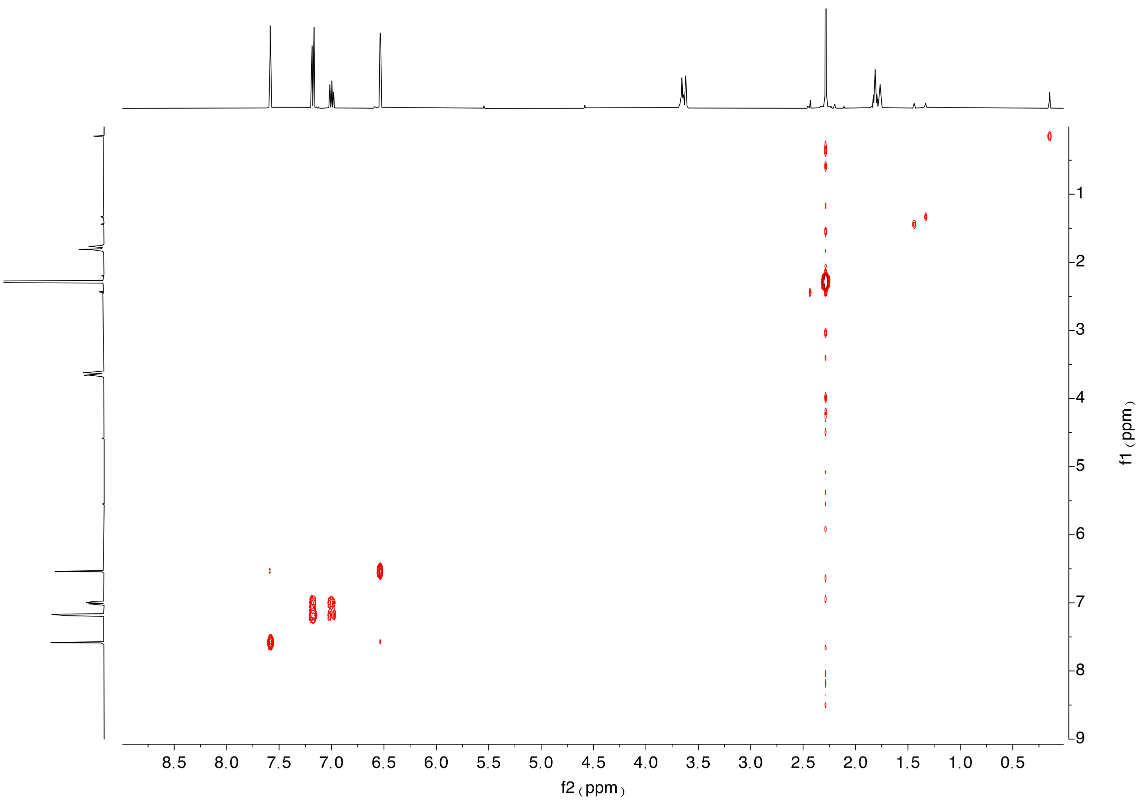

COSY NMR (400 MHz, THF-d_8) of [Fe^{II}(ImP)$_2$]

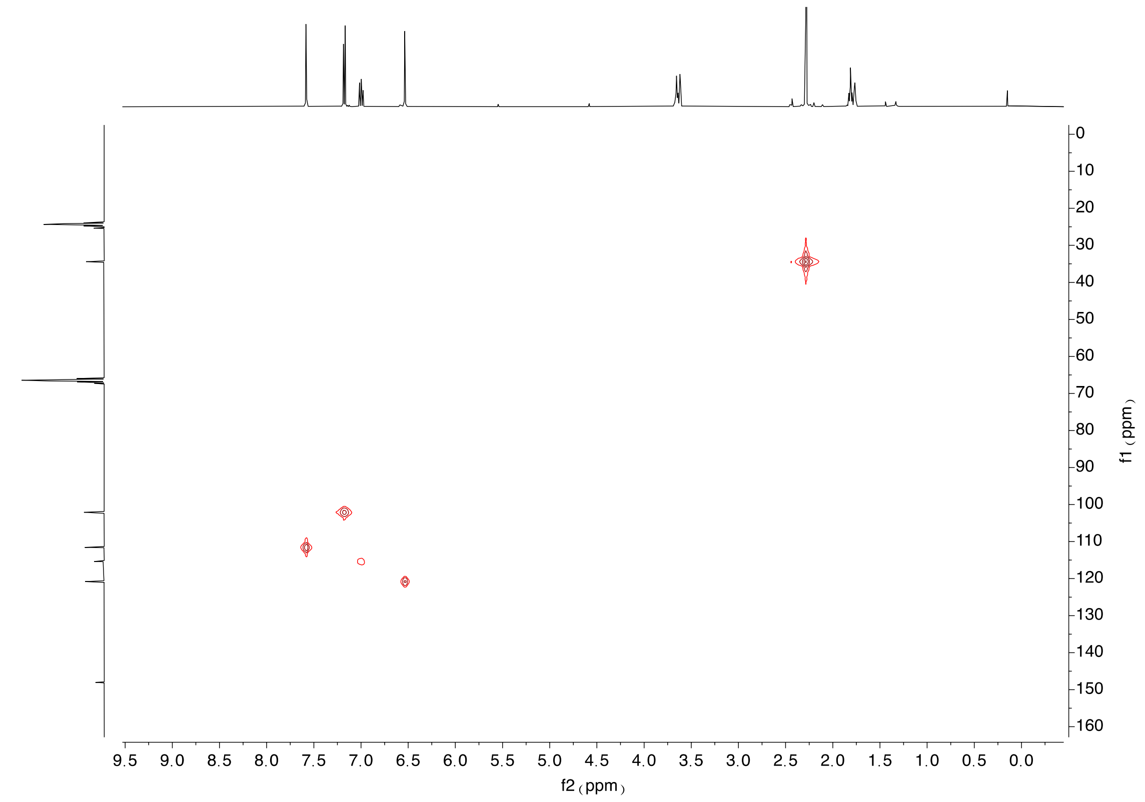

HMQC NMR (400 MHz, THF-d_8) of [Fe^{II}(ImP)$_2$]

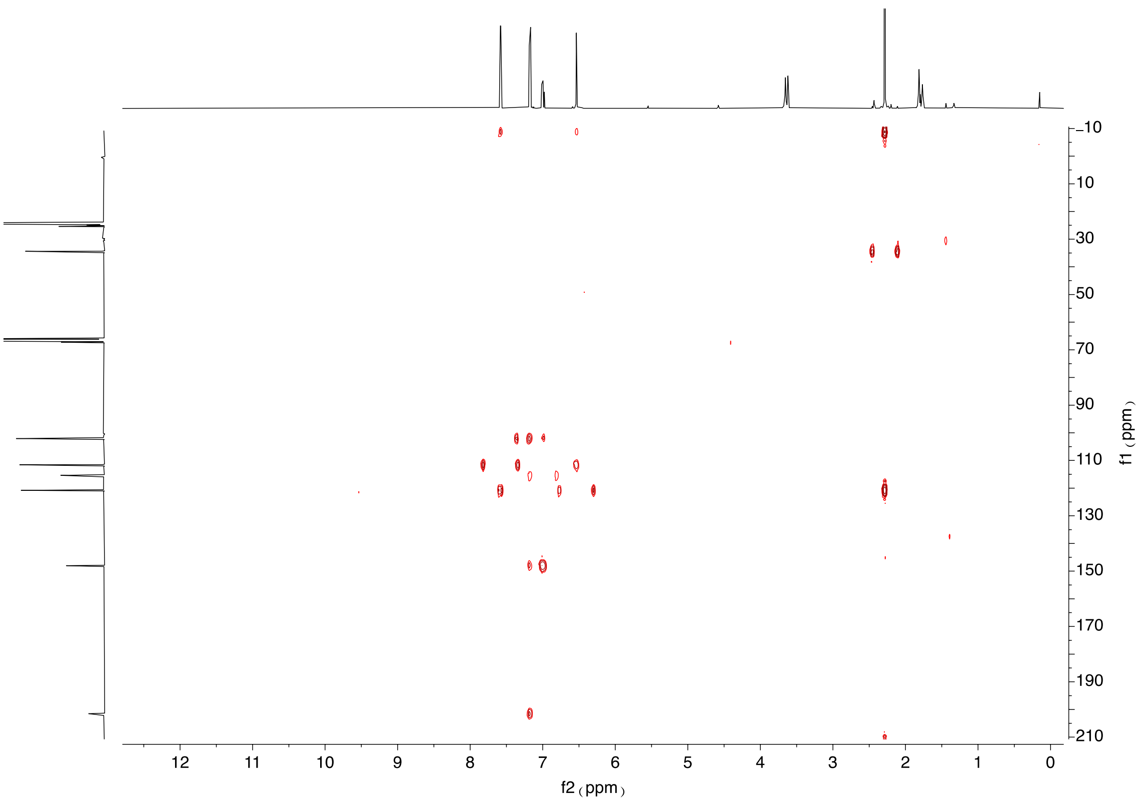

HMBC NMR (400 MHz, THF-d$_8$) of [Fe^{II}(ImP)$_2$]

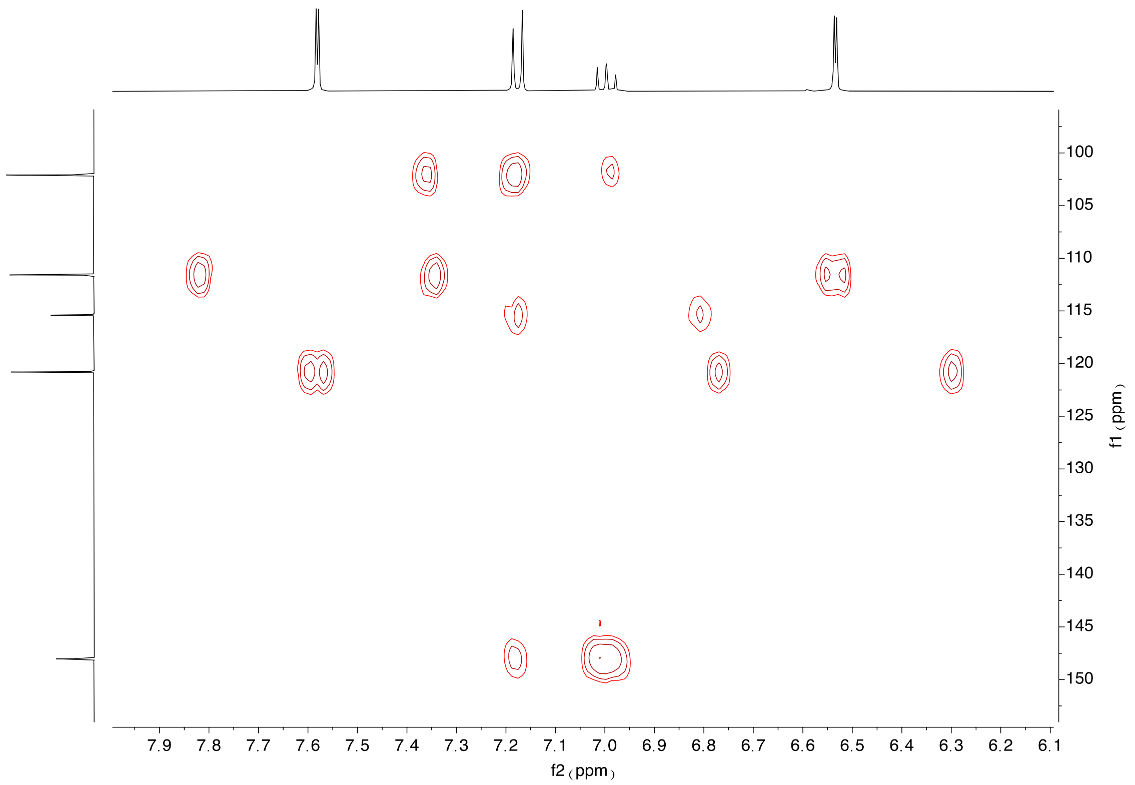

Expansion of HMBC NMR (400 MHz, THF-d$_8$) of [Fe^{II}(ImP)$_2$]

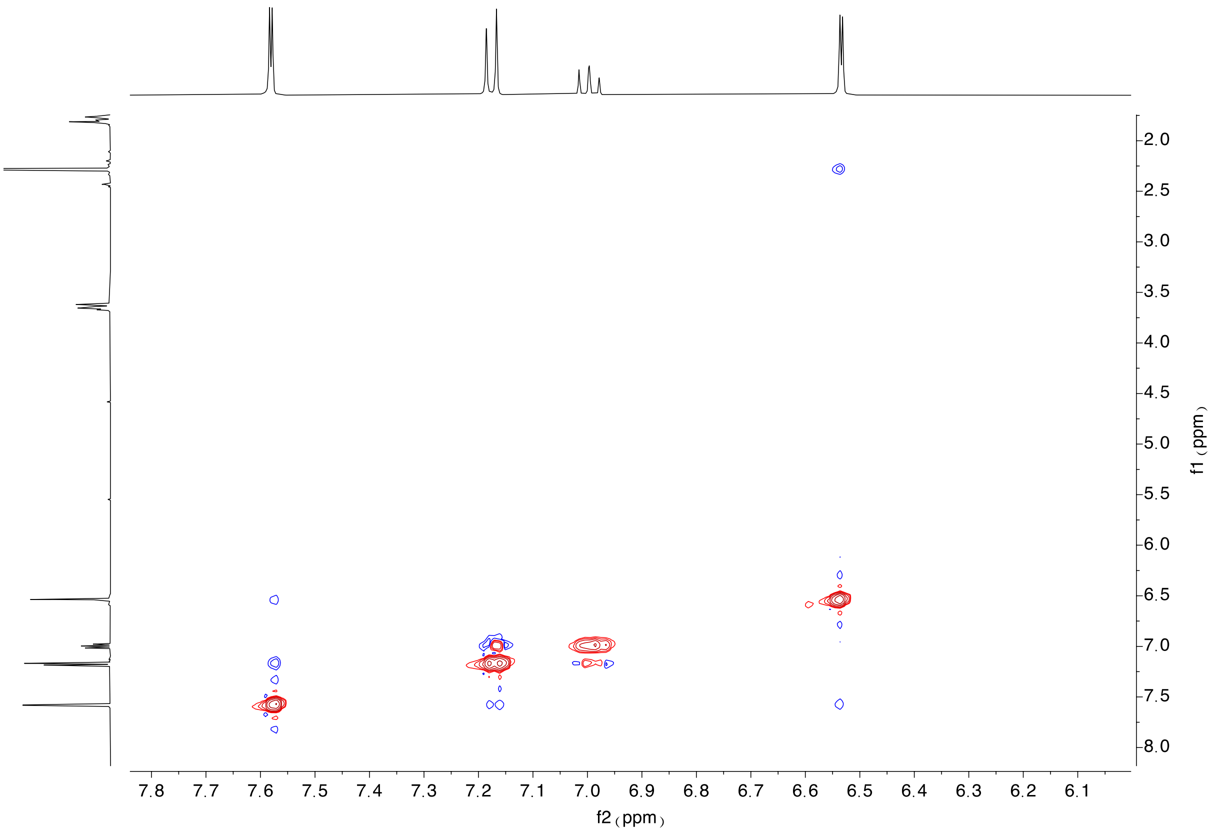

NOESY NMR (400 MHz, THF-d_8) of [$Fe^{II}(ImP)_2$]

HRMS

Electrospray ionization–high resolution mass spectrometry (ESI–HRMS) was recorded on a Waters Micromass Q-Tof micro mass spectrometer. HRMS using MALDI-TOF and ESI-TOF was attempted for $[Fe^{II}(ImP)_2]$, but we were only able to obtain the mass of the oxidized species $[Fe^{III}(ImP)_2]^+$. Elemental analyses were performed by Mikroanalytisches Laboratorium KOLBE (Mülheim an der Ruhr, Germany).

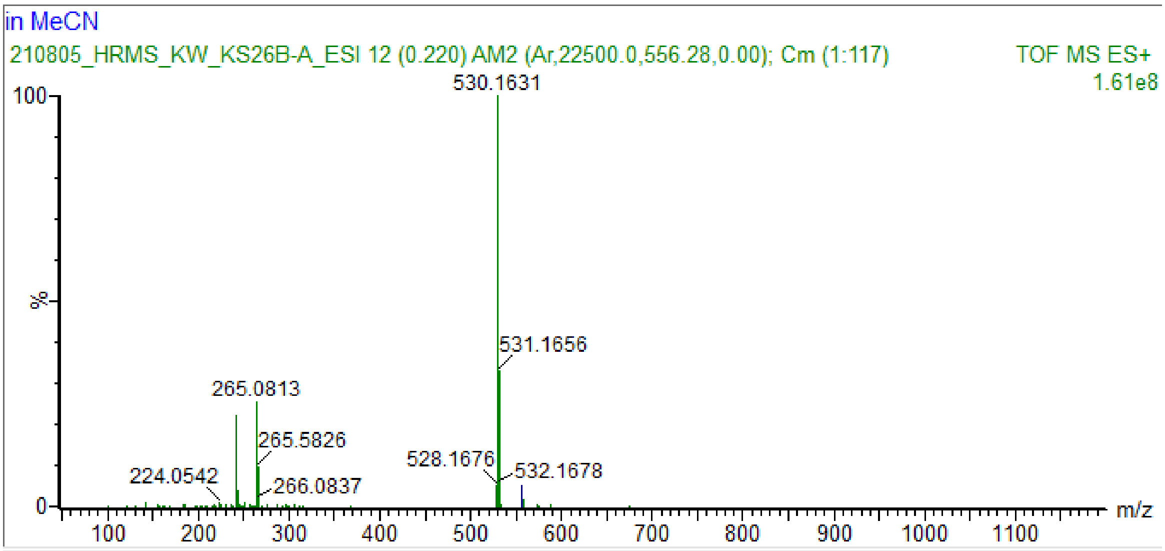

Single Mass Analysis
Tolerance = 2.0 mDa / DBE: min = -1.5, max = 100.0
Element prediction: Off
Number of isotope peaks used for i-FIT = 3
Monoisotopic Mass, Odd and Even Electron Ions
180 formula(e) evaluated with 2 results within limits (all results (up to 1000) for each mass)
Elements Used:
C: 0-70 H: 0-120 N: 3-10 O: 0-3 56Fe: 1-1

Mass	Calc. Mass	mDa	PPM	DBE	Formula	i-...	Fit Conf %	C	H	N	O	56Fe
530.1631	530.1630	0.1	0.2	20.0	C28 H26 N8 56Fe	60...	99.96	28	26	8		1
	530.1643	-1.2	-2.3	19.5	C30 H28 N5 O 56Fe	77...	0.04	30	28	5	1	1

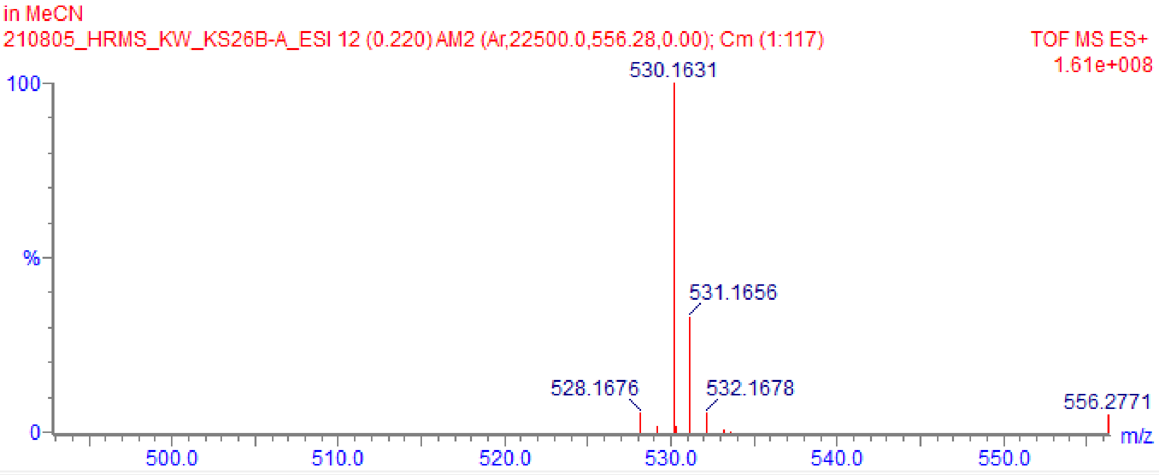

ESI-TOF of $[Fe^{III}(ImP)_2]PF_6$

Single crystal X-ray diffraction analysis

Table S1. Crystal data and structure refinement for $[Fe^{III}(ImP)_2]PF_6$.

Identification code	FeIIICAB-2CM_150	
Empirical formula	$C_{28}H_{26}F_6FeN_8P$	
Formula weight	675.39	
Temperature	150 K	
Wavelength	0.71073 Å	
Crystal system	Monoclinic	
Space group	*P 1 21/c 1*	
Unit cell dimensions	*a* = 23.1820(6) Å	*α* = 90°.
	b = 19.7134(3) Å	*β* = 102.186(2)°.
	c = 20.7973(4) Å	*γ* = 90°.
Volume	9290.1(3) $Å^3$	
Z	12	
Density (calculated)	1.449 Mg/m^3	
Absorption coefficient	0.608 mm^{-1}	
F(000)	4140	
Crystal size	0.3 x 0.2 x 0.18 mm^3	
Theta range for data collection	3.325 to 29.562°.	
Index ranges	-29<=h<=30, -26<=k<=26, -28<=l<=27	
Reflections collected	148479	
Independent reflections	22767 [*R*(int) = 0.1003]	
Completeness to theta = 25.242°	99.7 %	
Absorption correction	Semi-empirical from equivalents	
Max. and min. transmission	1.00000 and 0.90245	
Refinement method	Full-matrix least-squares on F^2	
Data / restraints / parameters	22767 / 0 / 1201	
Goodness-of-fit on F^2	0.992	

Final R indices [*I*>2sigma(I)]	*R1* = 0.0590, *wR2* = 0.1392
R indices (all data)	*R1* = 0.1172, *wR2* = 0.1645
Largest diff. peak and hole	1.010 and -0.551 e.Å^{-3}

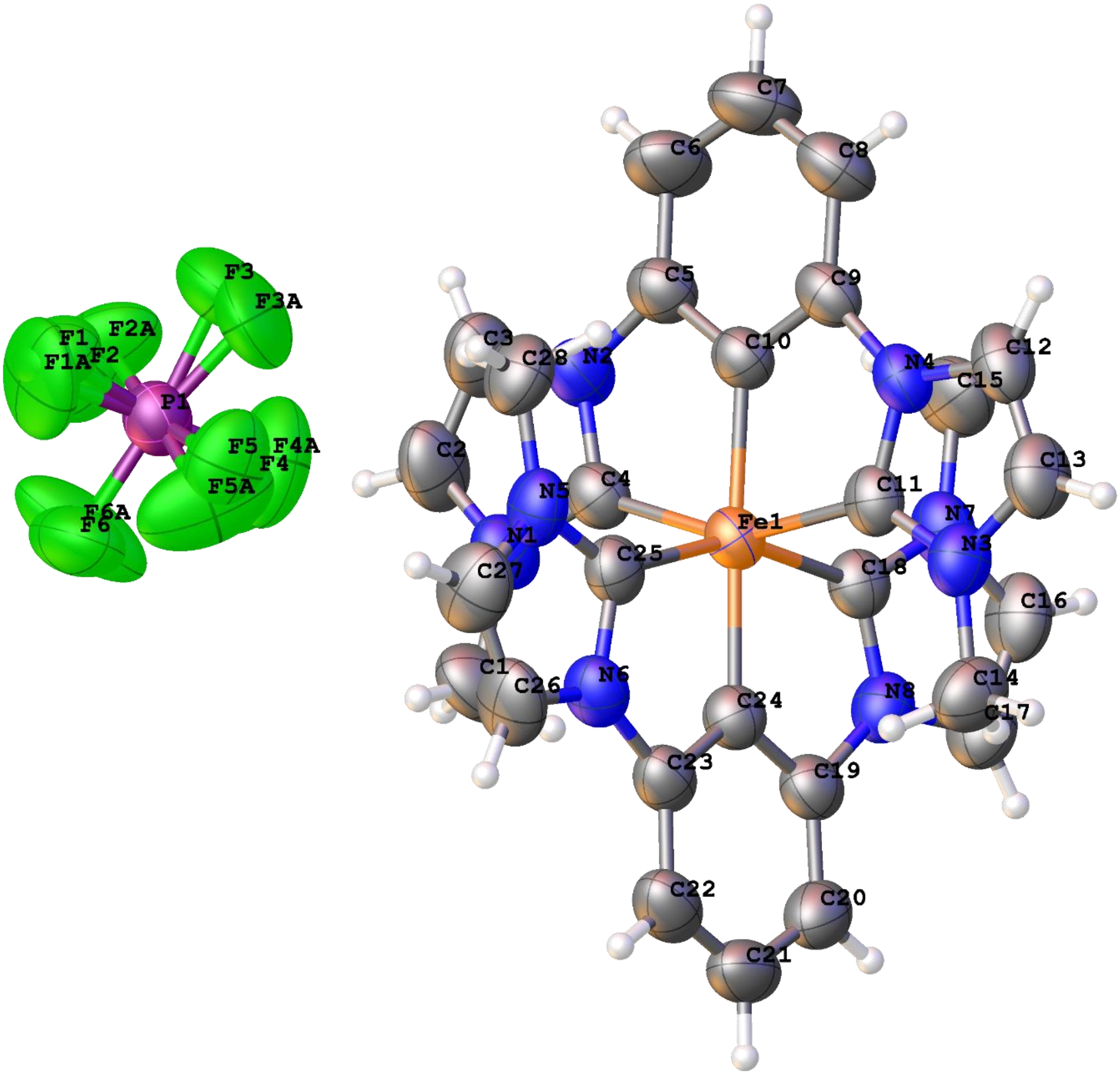

Fig. S1. Molecular view of $[Fe^{III}(ImP)_2]PF_6$. Hydrogen atoms and solvent molecules are omitted for clarity. The displayed atoms are C in black, F in green, Fe in orange, N in blue, and P in purple.

Table S2. Selected bond length and bond angles of $[Fe^{III}(ImP)_2]PF_6$.

**Compound**	**Bond lengths (Å)**	**Bond angles (°)**
$[Fe^{III}(ImP)_2]PF_6$	Fe(1)-C(1): 1.945(3)	C(1)-Fe(1)-C(7): 77.96(14)
	Fe(1)-C(7): 1.976(3)	C(1)-Fe(1)-C(11): 78.01(14)
	Fe(1)-C(11): 2.008(3)	C(1)-Fe(1)-C(15): 100.24(13)
	Fe(1)-C(15): 1.991(3)	C(1)-Fe(1)-C(25): 104.19(13)
	Fe(1)-C(19): 1.944(3)	C(7)-Fe(1)-C(11): 155.84(14)
	Fe(1)-C(25): 1.974(3)	C(7)-Fe(1)-C(15): 97.12(12)
	Fe(2)-C(29): 1.953(3)	C(15)-Fe(1)-C(11): 89.49(13)
	Fe(2)-C(37): 2.003(4)	C(19)-Fe(1)-C(1): 175.17(14)
	Fe(2)-C(41): 1.973(3)	C(19)-Fe(1)-C(7): 106.66(14)
	Fe(2)-C(43): 1.949(3)	C(19)-Fe(1)-C(11): 97.43(14)
	Fe(2)-C(49): 1.962(3)	C(19)-Fe(1)-C(15): 78.00(13)
	Fe(2)-C(53): 1.996(3)	C(19)-Fe(1)-C(25): 77.70(13)
	Fe(3)-C(57): 1.948(3)	C(25)-Fe(1)-C(7): 87.40(12)
	Fe(3)-C(66): 2.001(3)	C(25)-Fe(1)-C(11): 96.14(13)
	Fe(3)-C(69): 1.974(3)	C(25)-Fe(1)-C(15): 155.56(14)
	Fe(3)-C(71): 1.995(4)	C(29)-Fe(2)-C(37): 77.80(14)
	Fe(3)-C(79): 1.947(3)	C(29)-Fe(2)-C(41): 77.61(14)
	Fe(3)-C(83): 1.992(4)	C(29)-Fe(2)-C(49): 107.68(14)
		C(29)-Fe(2)-C(53): 97.43(14)
		C(41)-Fe(2)-C(37): 155.38(13)
		C(41)-Fe(2)-C(53): 92.52(13)
		C(43)-Fe(2)-C(29): 174.97(14)
		C(43)-Fe(2)-C(37): 102.94(13)
		C(43)-Fe(2)-C(41): 101.66(13)

		C(43)-Fe(2)-C(49): 77.22(13)<br>C(43)-Fe(2)-C(53): 77.61(13)<br>C(49)-Fe(2)-C(37): 97.34(15)<br>C(49)-Fe(2)-C(41): 88.94(14)<br>C(49)-Fe(2)-C(53): 154.56(14)<br>C(53)-Fe(2)-C(37): 91.84(13)<br>C(57)-Fe(3)-C(66): 77.86(14)<br>C(57)-Fe(3)-C(69): 77.83(14)<br>C(57)-Fe(3)-C(71): 103.91(14)<br>C(57)-Fe(3)-C(83): 101.00(15)<br>C(69)-Fe(3)-C(66): 155.69(14)<br>C(69)-Fe(3)-C(71): 91.74(14)<br>C(69)-Fe(3)-C(83): 89.56(13)<br>C(71)-Fe(3)-C(66): 93.99(14)<br>C(79)-Fe(3)-C(57): 178.61(16)<br>C(79)-Fe(3)-C(66): 101.92(14)<br>C(79)-Fe(3)-C(69): 102.39(14)<br>C(79)-Fe(3)-C(71): 77.47(16)<br>C(79)-Fe(3)-C(83): 77.64(16)<br>C(83)-Fe(3)-C(66): 95.13(13)<br>C(83)-Fe(3)-C(71): 154.77(15)

Table S3. Crystal data and structure refinement for [$Fe^{II}(ImP)_2$].

Identification code	FeIICAB-2CM	
Empirical formula	$C_{28}H_{22}FeN_8$	
Formula weight	526.38	
Temperature	150 K	
Wavelength	0.71073 Å	
Crystal system	Tetragonal	
Space group	*I-4*	
Unit cell dimensions	*a* = 8.36140(10) Å	*α* = 90°.
	b = 8.36140(10) Å	*β* = 90°.
	c = 18.0048(6) Å	*γ* = 90°.
Volume	1258.77(5) $Å^3$	
Z	2	
Density (calculated)	1.389 Mg/m^3	
Absorption coefficient	0.633 mm^{-1}	
F(000)	544	
Crystal size	0.28 x 0.2 x 0.1 mm^3	
Theta range for data collection	3.446 to 29.404°.	
Index ranges	-11<=h<=10, -11<=k<=10, -24<=l<=24	
Reflections collected	13245	
Independent reflections	1592 [*R*(int) = 0.0587]	
Completeness to theta = 25.242°	99.7 %	
Absorption correction	Semi-empirical from equivalents	
Max. and min. transmission	1.00000 and 0.93567	
Refinement method	Full-matrix least-squares on F^2	
Data / restraints / parameters	1592 / 86 / 86	
Goodness-of-fit on F^2	1.072	
Final *R* indices [*I*>2sigma(I)]	*R1* = 0.0893, *wR2* = 0.2242	
R indices (all data)	*R1* = 0.1076, *wR2* = 0.2463	

Absolute structure parameter	0.469(14)
Largest diff. peak and hole	1.375 and -0.701 e.Å^{-3}

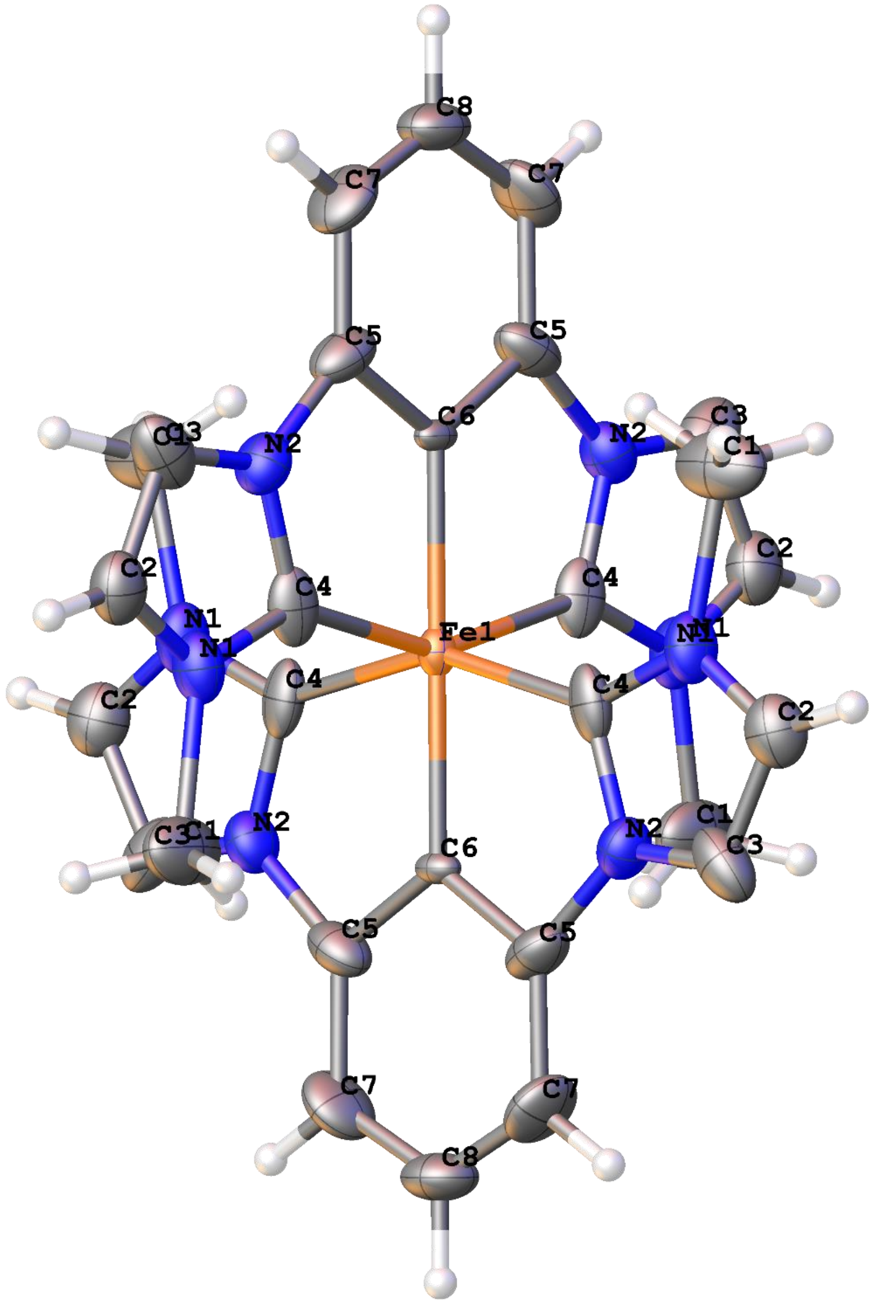

Fig S2. Molecular view of [Fe^{II}(ImP)$_2$]. Hydrogen atoms and solvent molecules are omitted for clarity. The displayed atoms are C in black, Fe in orange and N in blue.

Table S4. Selected bond length and bond angles of $[Fe^{II}(ImP)_2]$.

Compound	Bond lengths (Å)	Bond angles (°)
$[Fe^{II}(ImP)_2]$	Fe(1)-C(6)#1: 1.938(8)	C(6)#1-Fe(1)-C(6): 180.0
	Fe(1)-C(6): 1.938(8)	C(4)#3-Fe(1)-C(6): 76.7(2)
	Fe(1)-C(4)#1: 1.934(10)	C(4)#3-Fe(1)-C(6)#1: 103.3(2)
	Fe(1)-C(4)#2: 1.934(10)	C(4)#2-Fe(1)-C(6)#1: 76.7(2)
	Fe(1)-C(4): 1.934(10)	C(4)#1-Fe(1)-C(6): 103.3(2)
	Fe(1)-C(4)#3: 1.934(10)	C(4)-Fe(1)-C(6) : 76.7(2)
		C(4)#1-Fe(1)-C(6)#1: 76.7(2)
		C(4)-Fe(1)-C(6)#1: 103.3(2)
		C(4)#2-Fe(1)-C(6): 103.3(2)
		C(4)#3-Fe(1)-C(4): 153.4(4)
		C(4)#2-Fe(1)-C(4)#1: 153.4(4)
		C(4)#3-Fe(1)-C(4)#1: 93.04(9)
		C(4)#2-Fe(1)-C(4): 93.04(9)
		C(4)#1-Fe(1)-C(4): 93.04(9)
		C(4)#3-Fe(1)-C(4)#2: 93.04(9)
		C(5)-C(6)-Fe(1): 121.5(5)
		C(5)#3-C(6)-Fe(1): 121.5(5)

Magnetic susceptibility and magnetization measurements

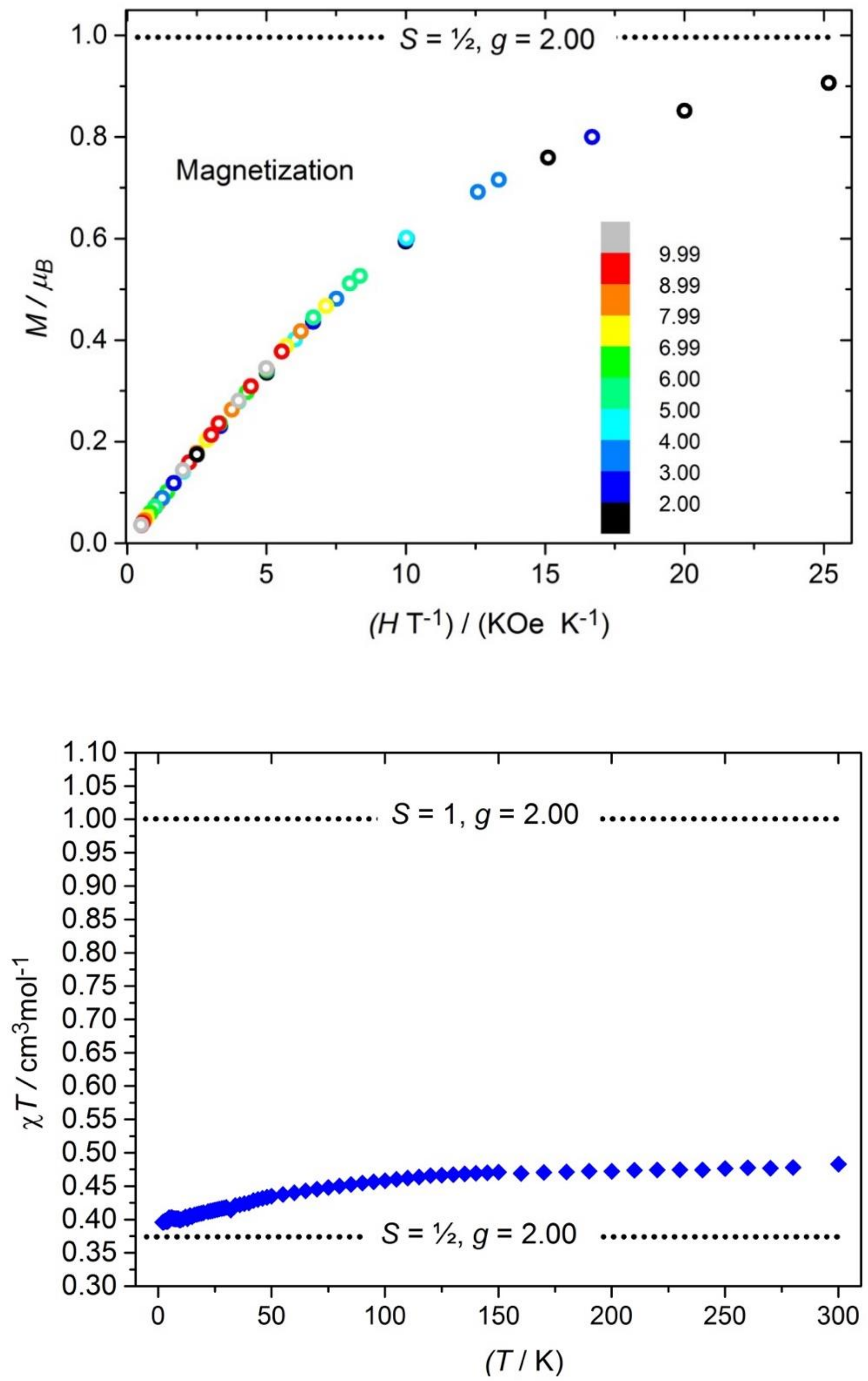

Fig. S3. Top panel: magnetization data of $[Fe^{III}(ImP)_2]PF_6$ recorded at fields 0.1 T – 5 T and temperatures 2 – 10 K. The insert color coding identifies the temperature. The superimposable curves for all fields are expected for an *S* = ½ spin-system. Bottom panel: Magnetic susceptibility versus temperature indicative of a system with a nearly quenched orbital moment.

Mößbauer spectroscopy

Table S5. Results of the fitting procedure of the 295 K and 85 K Mößbauer spectra. CS is the center shift relative natural Fe held at 295 K, |QS| is the magnitude of the electric quadrupole splitting , Γ_+ is the Lorentzian line width for the high velocity peak and Γ_- / Γ_+ is the ratio between the low and high velocity peak, respectively.

Complex	Temperature	CS mm/s	|QS| mm/s	Γ_+ mm/s	Γ_- / Γ_+
$[Fe^{III}(ImP)_2]PF_6$	295K	-0.193(5)	1.317(5)	0.304(5)	1.04(5)
	85 K	-0.115(5)	1.589(5)	0.389(5)	1.07(2)

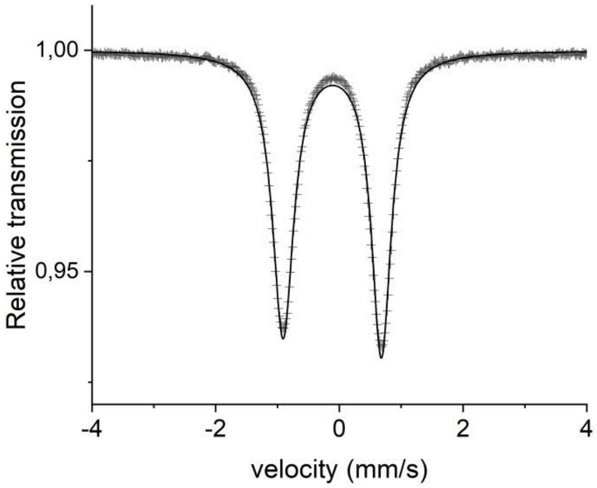

Fig. S4 Mößbauer spectrum of $[Fe^{III}(ImP)_2]PF_6$ at 85 K.

Stability experiments under illumination and in the dark

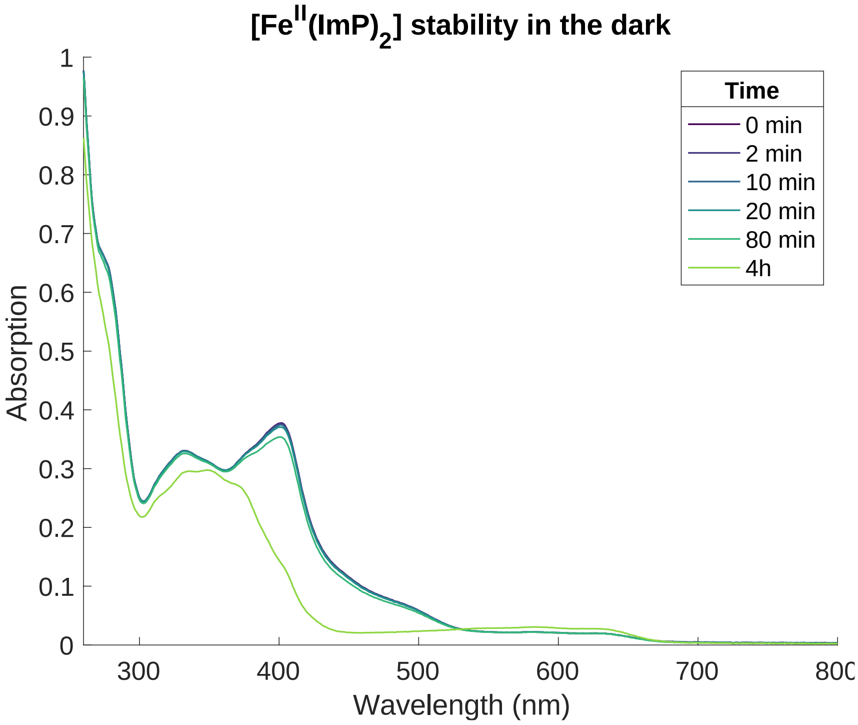

Fig. S5. UV-vis absorption spectrum of $[Fe^{II}(ImP)_2]$ (containing some $[Fe^{III}(ImP)_2]^+$) in a acetonitrile solution prepared under N_2 shows slow oxidation (by leakage of air) back to the Fe(III) state when stored in the dark.

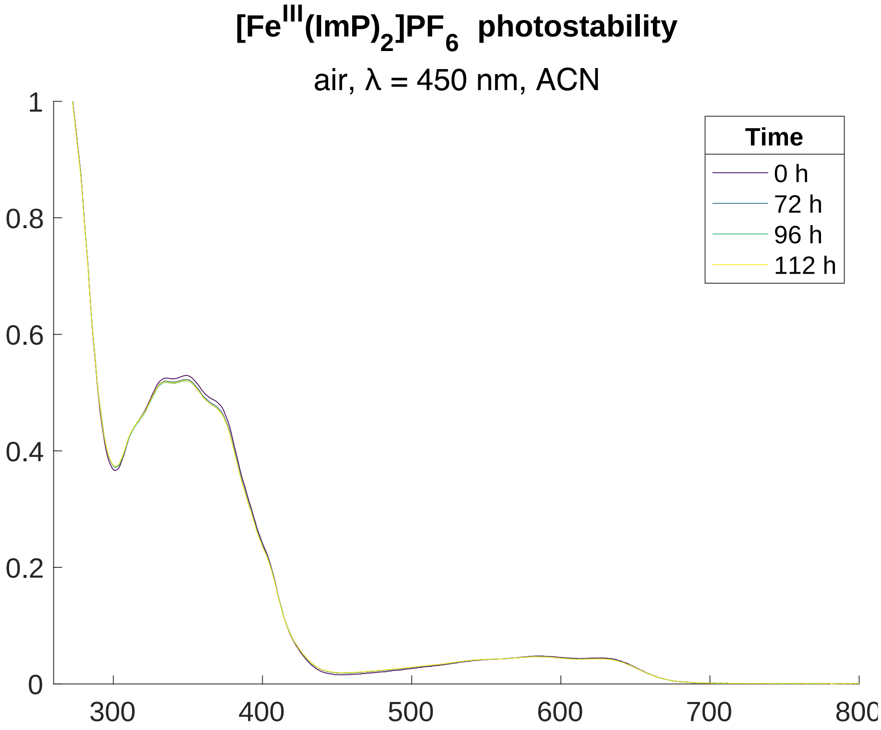

Fig. S6 UV-vis absorption spectrum of $[Fe^{III}(ImP)_2]PF_6$ in acetonitrile under air irradiated at $\lambda = 450$ nm.

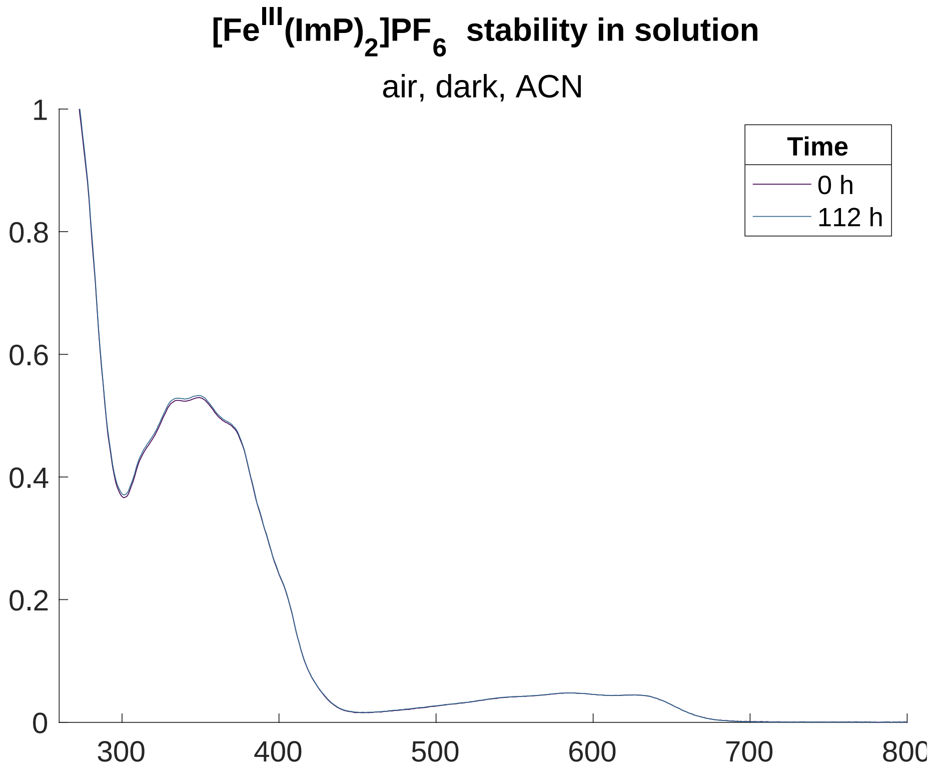

Fig. S7 UV-vis absorption spectrum of $[Fe^{III}(ImP)_2]PF_6$ in acetonitrile under air kept in the dark.

Electrochemistry and Spectroelectrochemistry

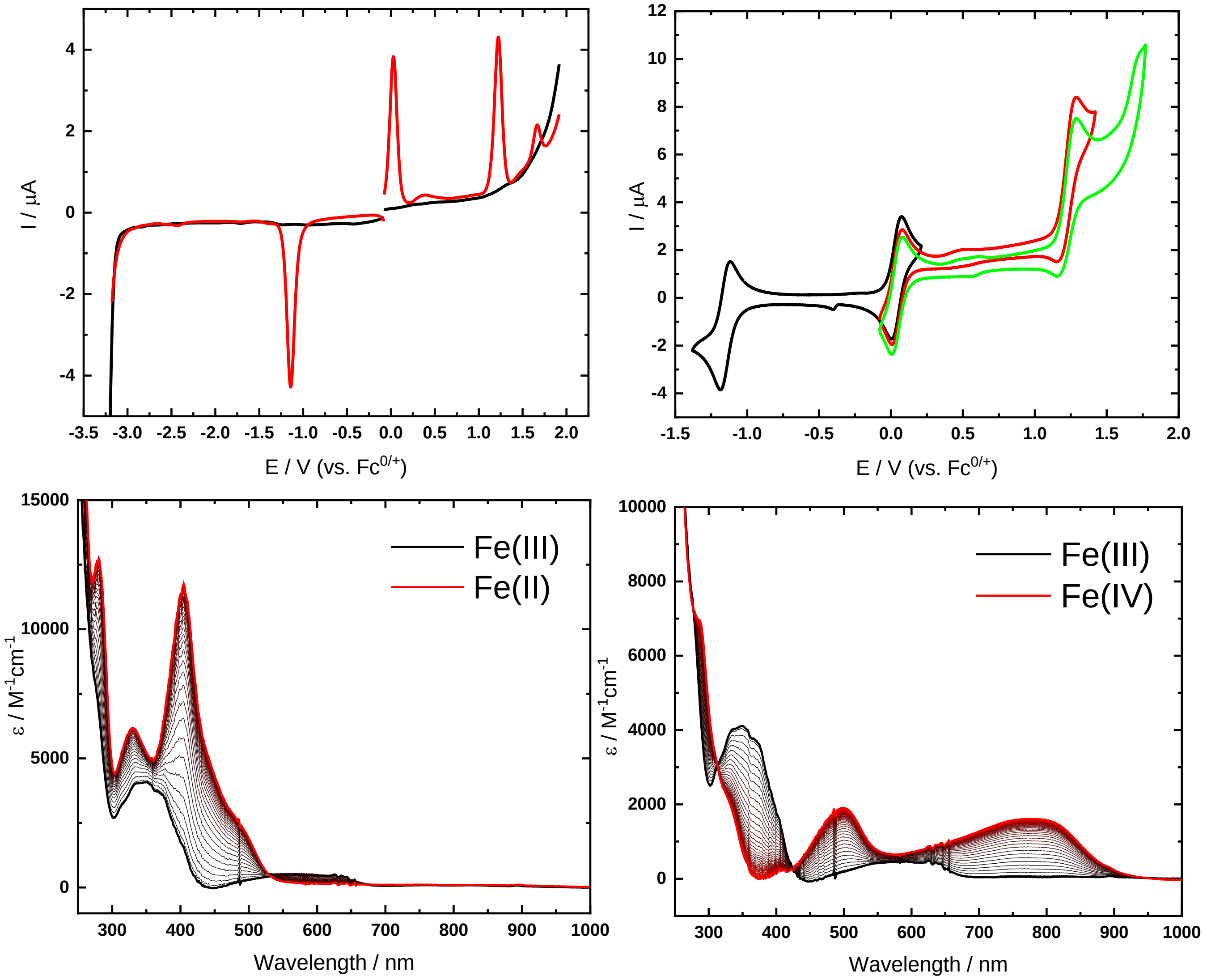

Fig. S8 Electro- and spectroelectrochemistry of 1 mM [Fe^{III}(ImP)$_2$]$^+$ in acetonitrile and 0.1 M TBAPF$_6$ as the electrolyte. **a)** Differential pulse voltammograms (step potential: 5 mV, modulation amplitude: 25 mV, modulation time: 50 ms, interval time: 100 ms). **b)** Cyclic Voltammograms (scan rate: 0.05 Vs^{-1}). **c)** UV-Vis absorption spectra during reduction of Fe(III) to Fe(II) (-1.38 V). **d)** UV-Vis absorption spectra during oxidation of Fe(III) to Fe(IV) (0.21 V).

The differential pulse and cyclic voltammograms are in good agreement with the recently reported electrochemistry measurements.[23] The report also mentions a suspected irreversible reduction of the ligand at around -2.7 V, which is however not observed in our measurements. Exhaustive, controlled potential electrolysis resulted in the clean conversion of [Fe^{III}(ImP)$_2$]$^+$ to its Fe(II) and Fe(IV) oxidation state, respectively. The resulting absorption spectra differ significantly from the reported spectra.[23]

Absorption spectra

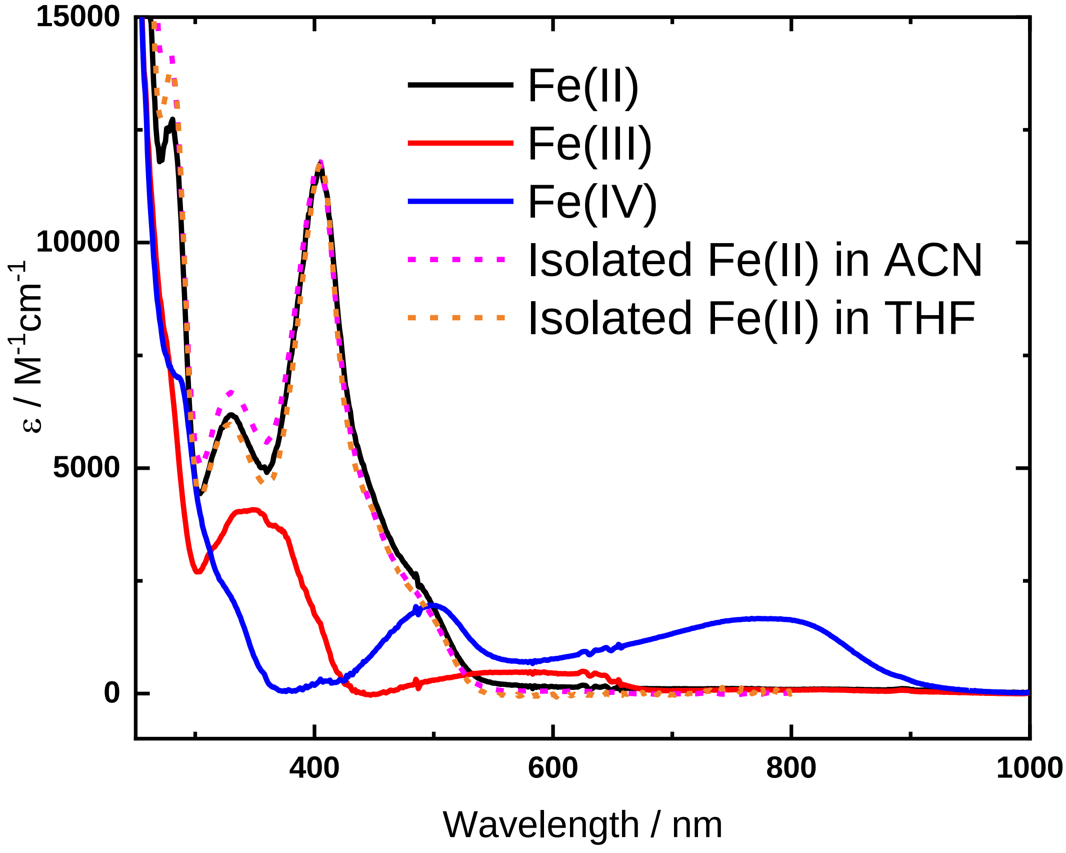

Fig. S9 Absorption spectra of $[Fe^{II}(ImP)_2]$, $[Fe^{III}(ImP)_2]^+$ and $[Fe^{IV}(ImP)_2]^{2+}$ in acetonitrile solution (with 0.1 M $TBAPF_6$) obtained from spectroelectrochemistry and of synthesized isolated $[Fe^{II}(ImP)_2]$ in acetonitrile and tetrahydrofuran solution.

TCSPC measurements of $[Fe^{II}(ImP)_2]$

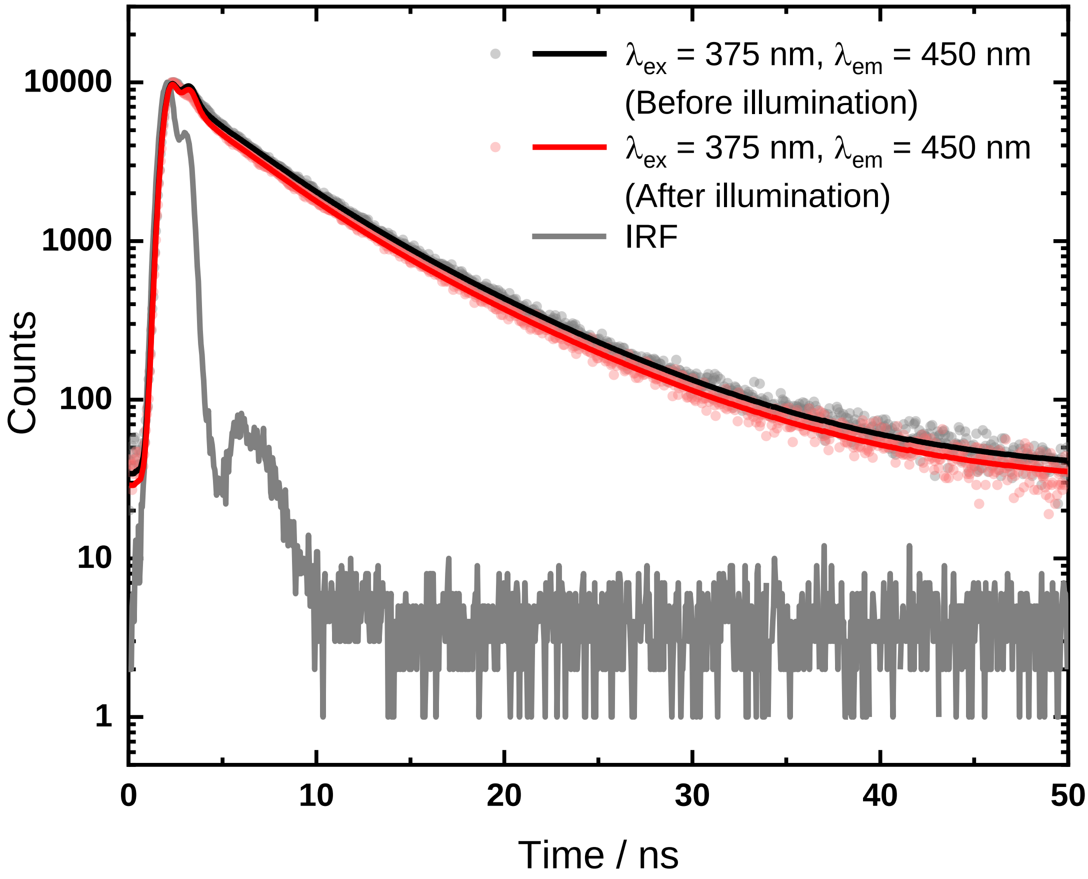

Fig. S10 TCSPC traces of the blue emission band (emission wavelength 450 nm) of $[Fe^{II}(ImP)_2]$ before illumination (3.5 (61%) and 7.6 (39%) ns; weighted average: 5.1 ns) and after illumination (3.5 (64%) and 7.6 (36%) ns; weighted average: 5.0 ns).

The lifetime of the pronounced blue emission observed after illumination of $[Fe^{II}(ImP)_2]$ agrees with the lifetime of the very weak emission detected before illumination of $[Fe^{II}(ImP)_2]$. The agreement corroborates the assignment of the original emission to a very minor amount of the same photoproduct accumulating under illumination.

Ligand precursor [HImP]$(PF_6)_2$ photophysics

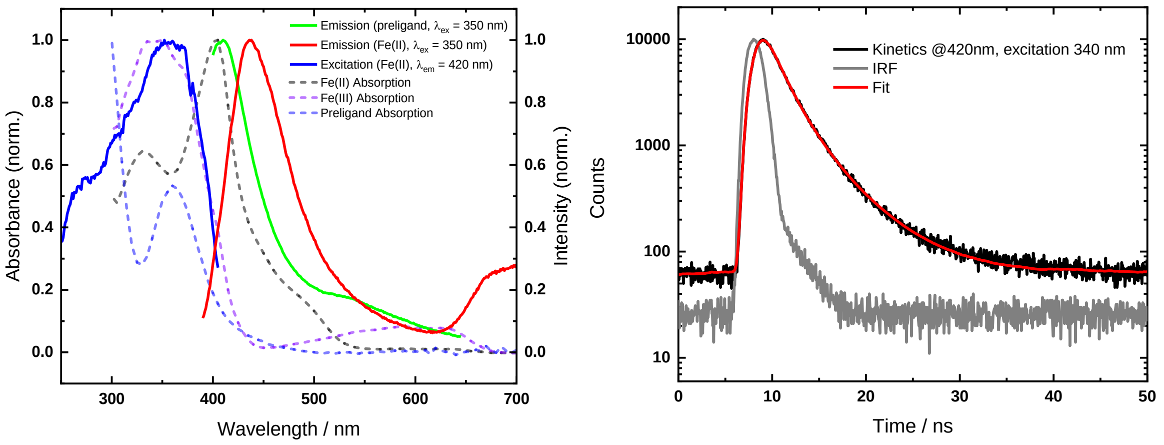

Fig. S11 a) Normalized UV-Vis absorbance, emission and excitation spectra of the ligand precursor $[HImP]^{2+}$ and $[Fe^{II}(ImP)_2]$ with its emissive photoproduct, all dissolved in acetonitrile; **b)** TCSPC trace at 420 nm of $[HImP]^{2+}$ excited at 340 nm.

The absorption spectrum of the ligand precursor $[HImP]^{2+}$ resembles very much the absorption and excitation spectra of the emissive photoproduct forming from $[Fe^{II}(ImP)_2]$. The emission peak of $[HImP]^{2+}$ is only slightly blue-shifted and the emission lifetime (~1.7 (83.7%) and 4.6 (16.3%) ns, weighted average 2.2 ns) is remarkably similar compared to the blue emission from the photoproduct. These similarities suggest that the photoproduct is structurally related to the ligand precursor, possibly forming via ligand detachment.

Fs-TAS of $[Fe^{III}(ImP)_2]^+$

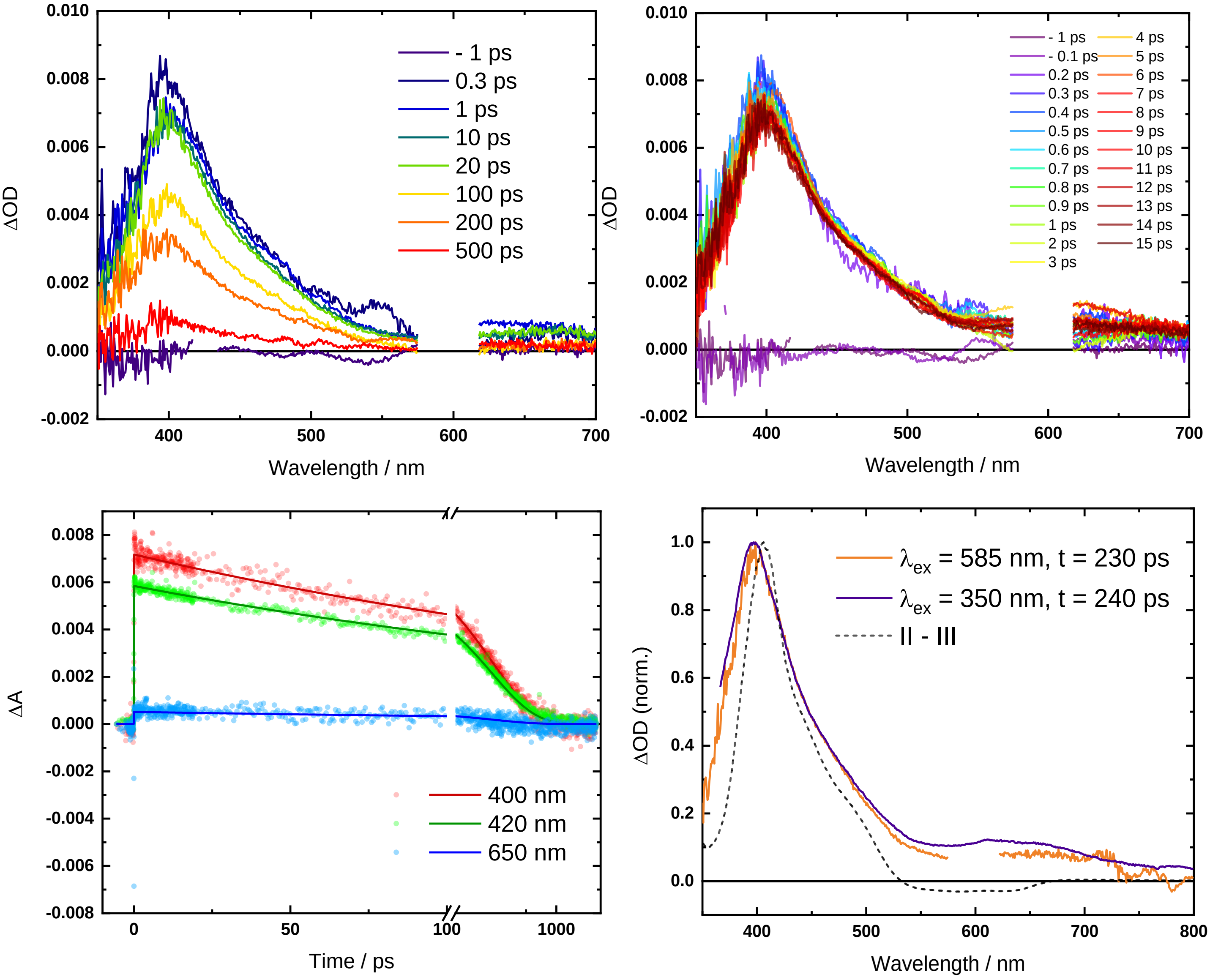

Fig. S12 Transient absorption measurements of $[Fe^{III}(ImP)_2]^+$ in deaerated acetonitrile solution. **a)** and **b)** Transient Absorption spectra at indicated time delays after photoexcitation (λ_{ex} = 585 nm, 140 fs, E = 0.44 ± 0.01 mW). **c)** Transient absorption kinetics at indicated wavelengths, fitted by Global Analysis. **d)** Normalized decay-associated spectra (DAS) from Global Analysis of transient absorption spectra of the ^{2}LMCT state of $[Fe^{III}(ImP)_2]^+$ with excitations at 350 and 585 nm and the differential absorption spectrum for the metal centered reduction of the complex.

Fs-TAS of $[Fe^{II}(ImP)_2]$ in tetrahydrofuran

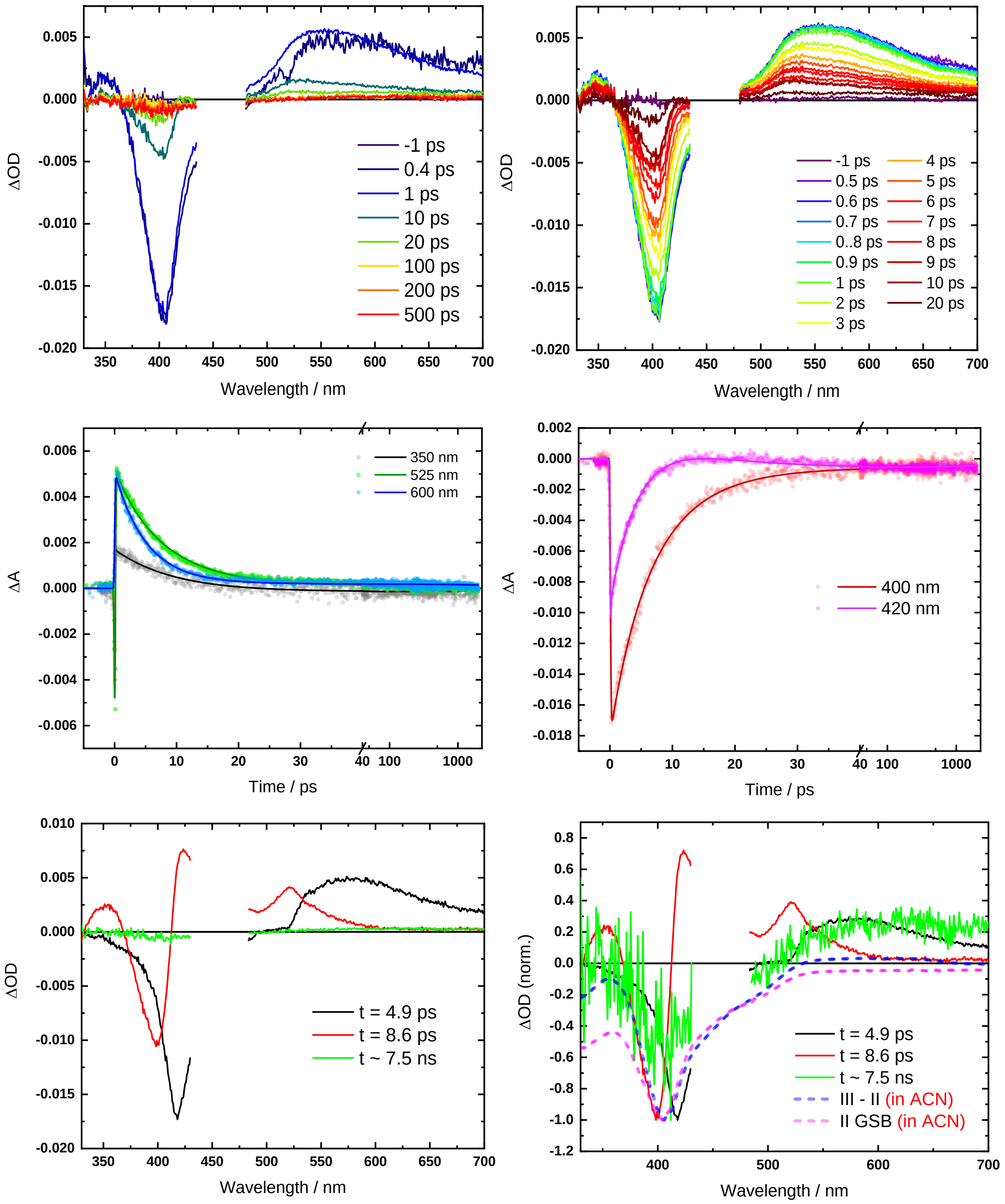

Fig. S13 Transient absorption measurements of $[Fe^{II}(ImP)_2]$ in tetrahydrofuran (THF) solution. **a)** and **b)** Transient Absorption spectra at indicated time delays after photoexcitation (λ_{ex} = 460 nm, 140 fs, E = 0.8 ± 0.03 mW). **c)** and d) Transient absorption kinetics at indicated wavelengths, fitted by Global Analysis. **e)** DAS from Global Analysis of transient absorption spectra. **f)** Normalized DAS along with the differential absorption spectrum for the metal centred oxidation of the complex and the expected ground-state bleach (GSB).

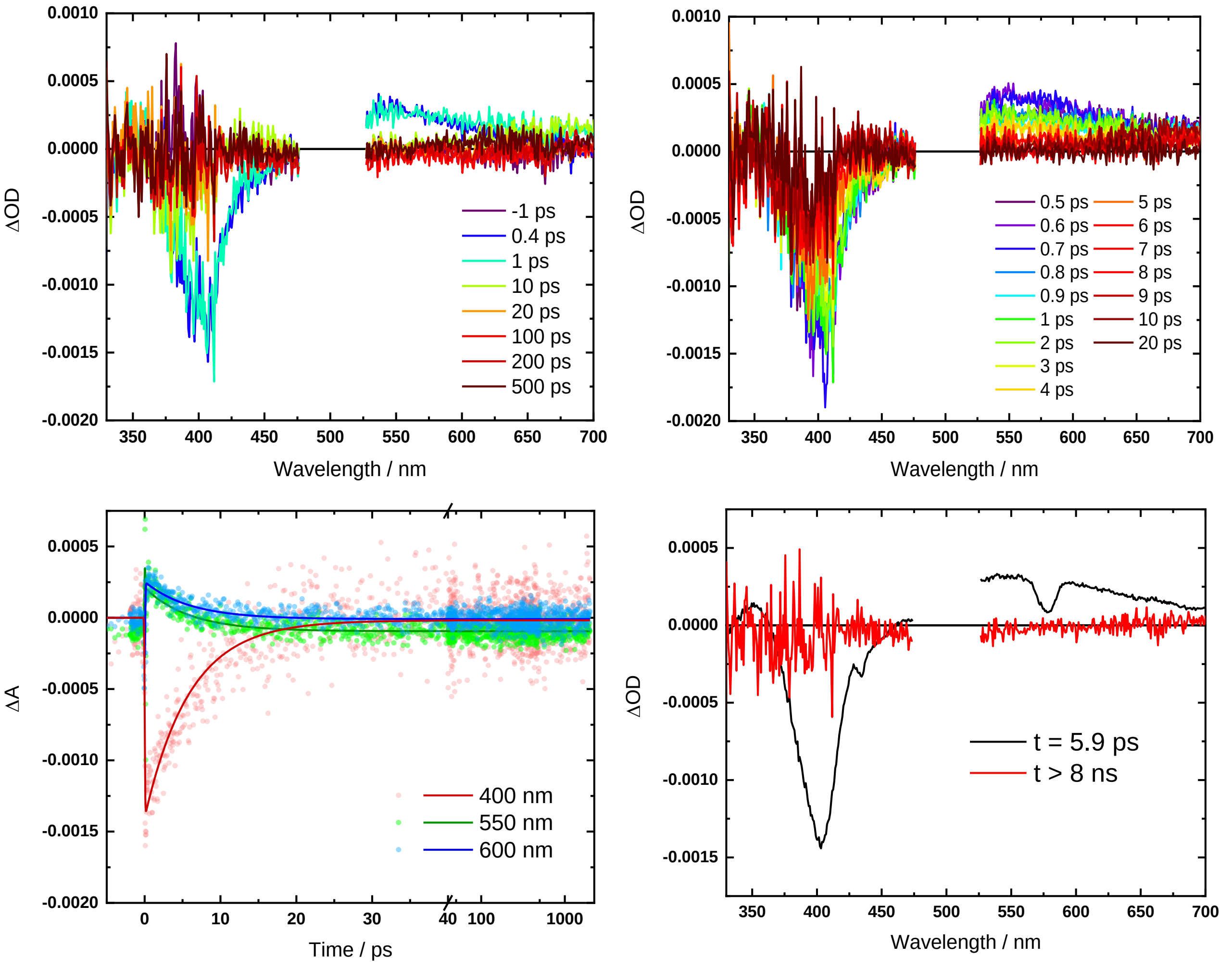

Fig. S14 Transient absorption measurements of [Fe^{II}(ImP)$_2$] in THF solution. **a)** and **b)** Transient Absorption spectra at indicated time delays after photoexcitation (λ_{ex} = 500 nm, 140 fs, E = 1 ± 0.03 mW). **c)** Transient absorption kinetics at indicated wavelengths, fitted by Global Analysis. **d)** DAS from Global Analysis of transient absorption spectra.

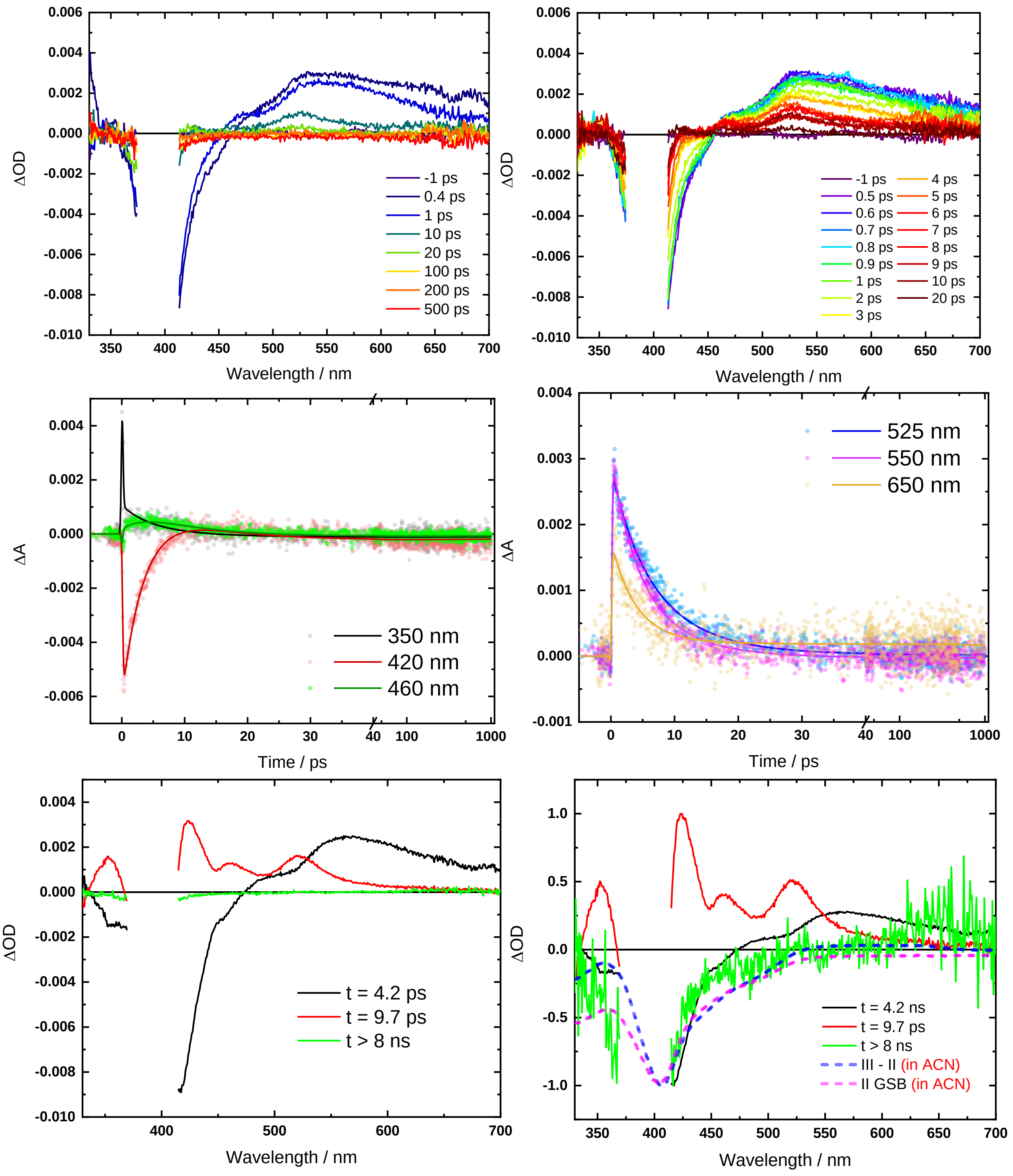

Fig. S15 Transient absorption measurements of [Fe^{II}(ImP)$_2$] in tetrahydrofuran (THF) solution. **a)** and **b)** Transient Absorption spectra at indicated time delays after photoexcitation (λ_{ex} = 400 nm, 140 fs, E = 3 ± 0.1 mW). **c)** and d) Transient absorption kinetics at indicated wavelengths, fitted by Global Analysis. **e)** DAS from Global Analysis of transient absorption spectra. **f)** Normalized DAS along with the differential absorption spectrum for the metal centred oxidation of the complex and the expected ground-state bleach (GSB).

DFT calculations

Optimized structures of Fe(II) and Fe(III)

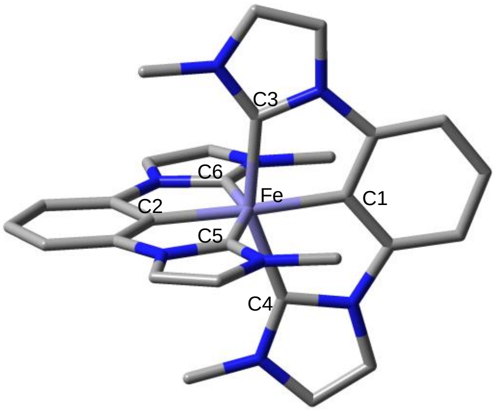

Fig. S16 Structure of $[Fe(ImP)_2]^{n+}$ complex with atom labels for the coordination environment. Hydrogen atoms are omitted for clarity.

Table S6 List of experimental and calculated bond distances (in Å) for $[Fe^{III}(ImP)_2]^+$.

	Experimental [a]	Calculated		
		^{2}GS	^{4}MC	^{6}MC
Fe-C1	1.947	1.960	2.017	2.131
Fe-C2	1.936	1.960	1.972	2.131
Fe-C3	1.991	2.011	2.252	2.256
Fe-C4	1.978	1.997	2.251	2.248
Fe-C5	1.988	2.010	2.108	2.252
Fe-C6	1.983	2.000	2.108	2.250
Average (Fe-C)	1.971	1.990	2.118	2.211

Table S7 List of experimental and calculated bond distances (in Å) for $[Fe^{II}(ImP)_2]$.

	Experimental [a]	Calculated		
		^{1}GS	^{3}MC	^{5}MC
Fe-C1	1.939	1.940	2.048	2.133
Fe-C2	1.939	1.940	1.976	2.133
Fe-C3	1.933	1.972	2.971	2.433

Fe-C4	1.933	1.977	2.122	2.326
Fe-C5	1.933	1.971	2.011	2.454
Fe-C6	1.933	1.977	2.041	2.320
Average (Fe-C)	1.935	1.963	2.195	2.300

Ground state electronic structure of $[Fe^{III}(ImP)_2]^+$

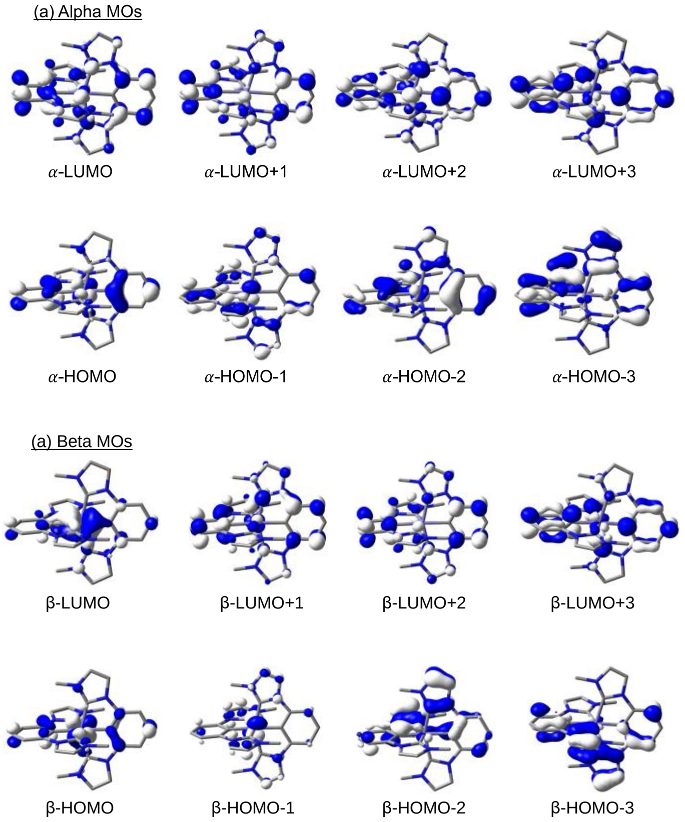

Fig. S17 Calculated molecular orbitals of alpha (a) and beta (b) frontier MOs (HOMO-2 to LUMO+2) for doublet $[Fe^{III}(ImP)_2]^+$ complex using B3LYP+D2/6-311G*, SDD(Fe) in CH_3CN. Contour isovalue of 0.04 e/Å^3.

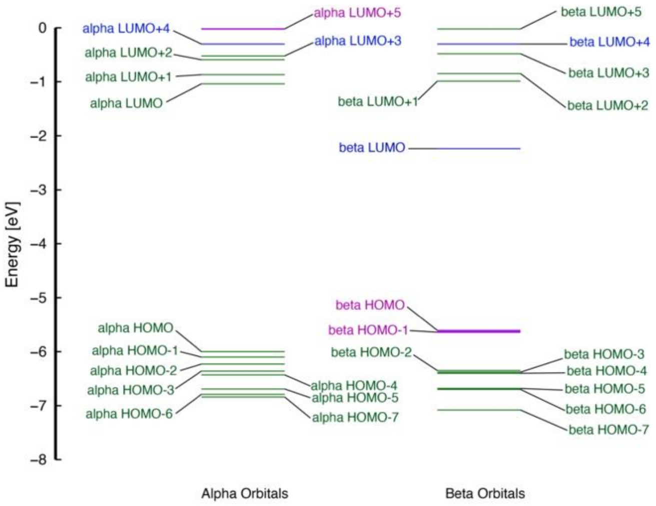

Fig. S18 Calculated molecular orbital energy level diagram of the doublet ground state of $^2[Fe(ImP)_2]^+$ using B3LYP+D2/6-311G*, SDD(Fe) in CH_3CN. Lines in blue are metal based orbitals, green are ligand-based orbitals, and purple are mixed orbitals (metal-ligand).

Electronic absorption spectrum of $[Fe^{III}(ImP)_2]^+$

The UV-Vis absorption spectra of the Fe(III) complex is shown in Fig. S19. The complex displays three main absorption bands. The band at higher energy (< 270 nm) is assigned to ligand-centered (LC) π-π* transition, while the lowest energy band (370-425 nm) corresponds to a ligand-to-metal charge transfer (LMCT). The final band with a shoulder between 350 and 280 nm comprises of transitions with mixed states: metal-to-ligand CT (MLCT), metal-centered (MC), LC, LMCT. The intense peak around 284 nm (f=0.049) shows a transition of ~ 50% MLCT character with LC and LMCT contributions (see Fig S19).

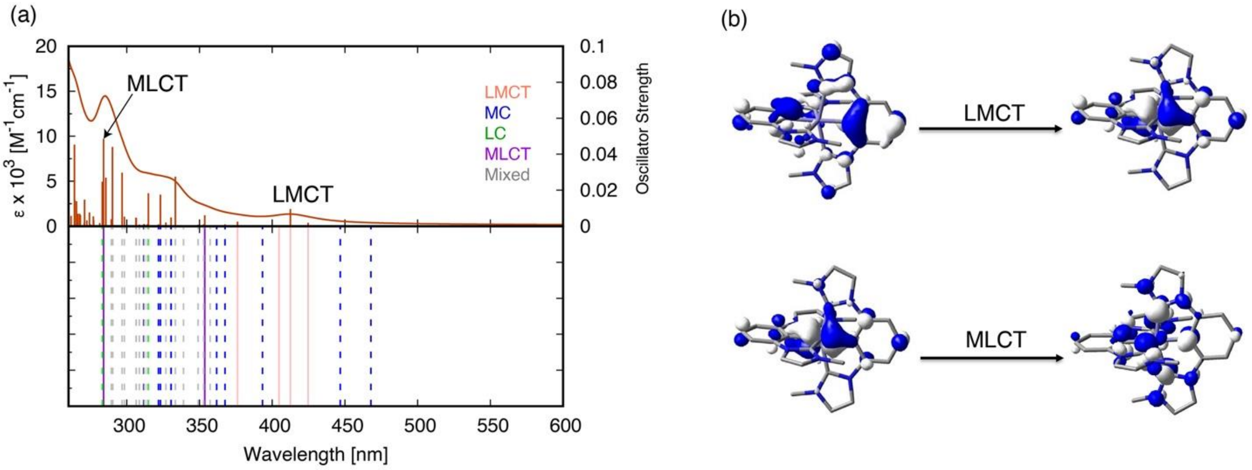

Fig. S19 (a) Calculated electronic absorption spectra of $[Fe^{III}(ImP)_2]^+$ using TD-B3LYP+D2/6-311G*, SDD(Fe) in CH_3CN. (b) Natural transition orbitals (NTOs) hole-particle pairs for the LMCT and MLCT states. LMCT denotes ligand-to-metal charge transfer, MLCT metal-to-ligand charge transfer, MC metal-centered, LC ligand-centered.

Potential energy curves of $[Fe^{III}(ImP)_2]^+$

Fig. S20 shows the vertical excitation potential energy curves (PECs) for the relevant electronic states of the $[Fe^{III}(ImP)_2]^+$ complex. From the plot (and Table S8), the doublet ground state (^{2}GS) is the most stable electronic state at all conformations investigated. The metal-centered states, ^{4,6}MC, are calculated to be high in energy at the optimized doublet geometry, with the relaxed minima states displaced to much longer Fe-C bond lengths. This is indicative of the strong σ-donating capability of the ligand, hence the destabilization of the metal-centered states. Excitation of the ^{2}GS leads to excited states with transitions characterized as ^{2}LMCT and ^{2}MLCT' (^{2}MLCT) states. Deactivation from the ^{2}LMCT state leads back to the ^{2}GS leads to a radiative decay since an ISC into ^{4}MC is spin-forbidden. However, upon excitation into the higher ^{2}MLCT states various decay pathways are likely to occur: (1) ^{2}MLCT → ^{2}LMCT → ^{2}GS or (2) ^{2}MLCT → ^{2}MLCT' → ^{2}GS which were observed in the experiment.

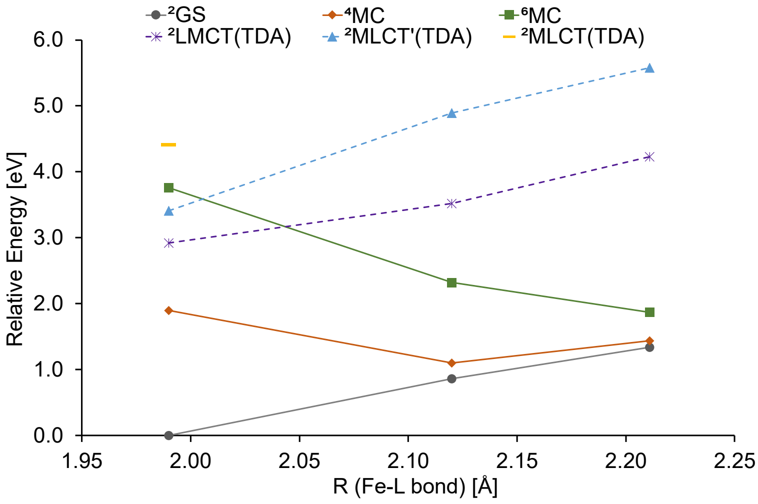

Fig. S20 Potential energy curves for the relevant electronic states of $[Fe^{III}(ImP)_2]^+$ from energies obtained at the ^{2}GS (R = 1.99 Å), ^{4}MC (R = 2.12 Å), and ^{6}MC (R = 2.21 Å) optimized structures from single point energy calculations at the DFT (^{2}GS, ^{4}MC, ^{6}MC) and TD-DFT levels of theory (^{2}LMCT) utilizing ^{2}GS as the reference state. The reaction coordinate is given as the average of Fe-L bond lengths at each optimized structure.

Table S8 Quantum chemically calculated relaxed states for $[Fe^{III}(ImP)_2]^+$ complex at B3LYP+D2/6-311G*, SDD(Fe) in acetonitrile.

	Geometry	E(eV)	Fe spin density	R (Å)
	^{2}GS	0.00	0.92	1.99
$[Fe(ImP)_2]$	^{4}MC	1.10	2.64	2.12
	^{6}MC	1.87	4.15	2.21

Ground-state electronic structure of [Fe^{II}(ImP)$_2$]

The calculated geometry of the various electronic states of [Fe^{II}(ImP)$_2$] are reported in Table S16. The fully optimized ^{3}MC and ^{5}MC states were identified based on natural orbital (NO) analysis (see Fig. S21).

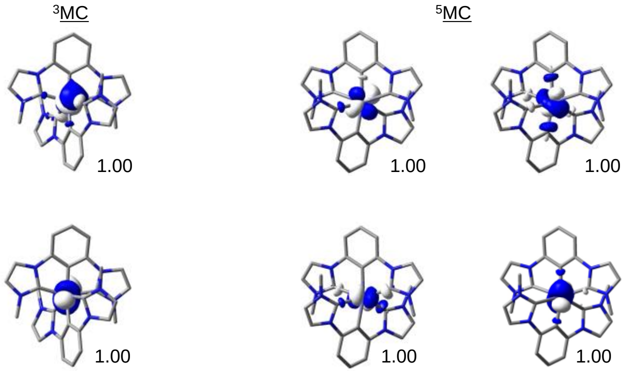

Fig. S21 Plots of singly occupied natural orbitals (SONO) with their occupation numbers for ^{3}MC and ^{5}MC (isovalue: 0.04 e/Å^3).

The computed average Fe-C bond distances display an increase from 1.96 Å in the ^{1}GS to 2.20 Å in the ^{3}MC state and 2.30 Å in the ^{5}MC state. The ^{3}MC state shows a deviation of 0.23 Å from the ^{1}GS with a greater deviation observed from three Fe-C bonds, in which one of the Fe-C (carbene) bonds pops out (see Fig. S22 and Table S6). The ^{5}MC also shows a very substantial deviation from the ^{1}GS with an average of 0.34 Å extension of Fe-C bond: all the six Fe-C bonds are elongated. This suggest that the ^{5}MC geometry is significantly different from the ^{1}GS, thus, the ^{5}MC state is further away and inaccessible for deactivation.

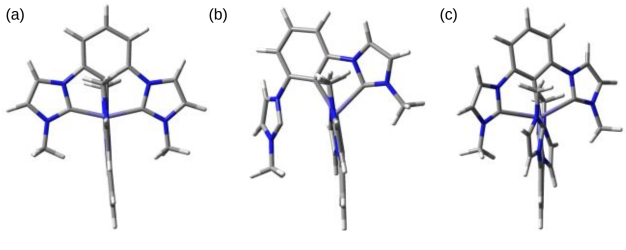

Fig. S22 Geometry optimized structures of [Fe^{II}(ImP)$_2$] at the ^{1}GS (a), ^{3}MC (b) and ^{5}MC (c) spin states using B3LYP+D2/6-311G*, SDD(Fe) in CH_3CN.

Molecular orbitals (MOs) and energy level diagram for the ground state (^{1}GS) of [Fe^{II}(ImP)$_2$] are shown in Fig. S23 & S24. The highest occupied molecular orbitals (HOMO through HOMO-2) are predominantly iron t_{2g} based (shown in blue). Occupied MOs at lower energies (HOMO-3 through HOMO-7) are ligand-based orbitals (shown in green). The lowest unoccupied molecular orbitals (LUMO through LUMO+7) are all ligand-based orbitals, except for LUMO+2 which is a Rydberg state (shown in

grey). Knowledge about the ground state frontier orbitals may provide insights into the type of transitions for the excited states. In this case, the first three HOMOs being metal t_{2g} based and the LUMOs (LUMO to LUMO+4) being ligand π* orbitals suggest that the lowest energy transitions would be metal-to-ligand charge transfer (MLCT), which are of interest for photophysical applications.

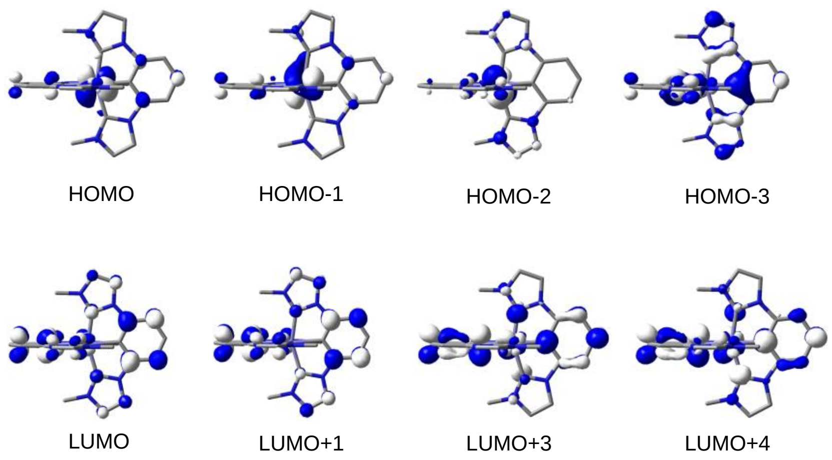

Fig. S23 Calculated molecular orbitals (HOMO-4 to LUMO+3) of the singlet ground state of $[Fe^{II}(ImP)_2]$ using B3LYP+D2/6-311G*, SDD(Fe) in CH_3CN. Contour isovalue of 0.04 e/Å^3.

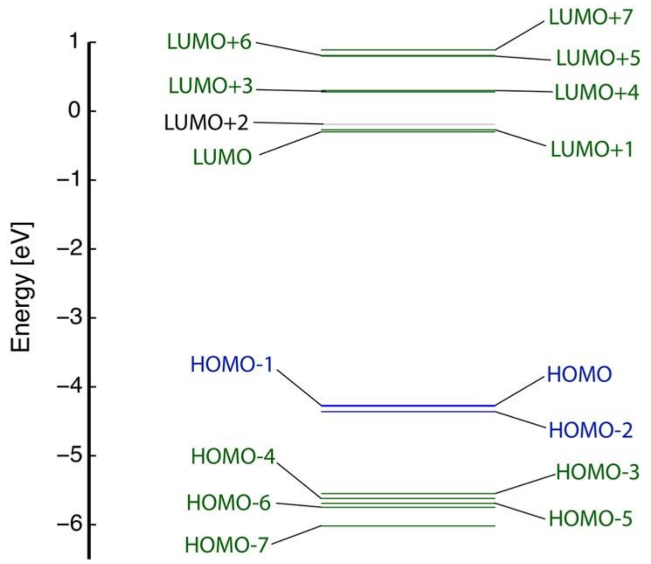

Fig. S24 Calculated molecular orbital energy level diagram of the singlet ground state of $[Fe^{II}(ImP)_2]$ using B3LYP+D2/6-311G*, SDD(Fe) in CH_3CN. Lines in blue are metal based (t_{2g}) in character, green are ligand-based orbitals, and grey is a Rydberg-type d orbital.

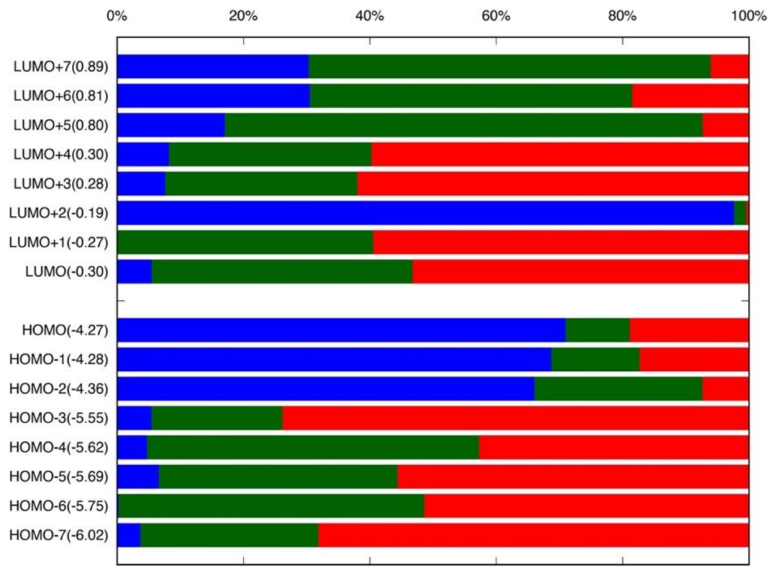

Fig. S25 Fragment molecular orbital analysis for [$Fe^{II}(ImP)_2$].

Electronic absorption spectrum of [$Fe^{II}(ImP)_2$]

The simulated absorption spectrum is similar to the experimentally observed spectrum of the Fe(II) complex. For the Fe(II) complex, the calculated absorption shows the lowest absorption band consists of almost degenerate energy levels at 428 nm (f=0.0197) and 424 nm (f=0.0182) whose transitions originates from HOMO to LUMO+1 (83%) and HOMO-1 to LUMO+1 (75%) respectively. The assignment of these transitions, HOMO (and HOMO-1) to LUMO+1, is metal-to-ligand charge transfer (MLCT). The strongest absorption peak which occurs at 345 nm (f=0.211) is also assigned to a MLCT transition. This transition comprises of contributions from HOMO-1 to LUMO+4 (41%), HOMO to LUMO+3 (27%), and HOMO-2 to LUMO+1/ LUMO+4 (14%). Several MLCT transitions with significant absorptivity are also observed. The presence of several low-lying MLCT states is consistent with the MO energy level diagram, which in general can be used as an approximation to describe the type of transitions in the excited states.

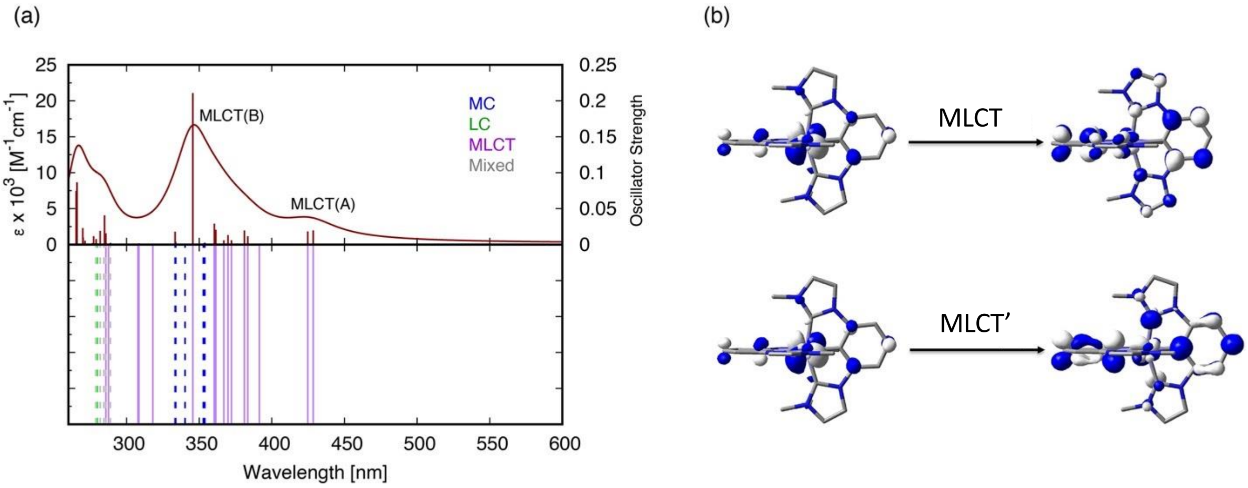

Fig. S26 (a) Calculated electronic absorption spectra of $[Fe^{II}(ImP)_2]$ using TD-B3LYP+D2/6-311G*, SDD(Fe) in CH_3CN. (b) Natural transition orbitals (NTOs) hole-particle pairs for the MLCT' and MLCT states. MLCT denotes metal-to-ligand charge transfer, MC metal-centered, LC ligand-centered.

Potential energy curves of $[Fe^{II}(ImP)_2]$

Fig. S28 shows the vertical excitation potential energy curves (PECs) for the relevant electronic states of the $[Fe^{II}(ImP)_2]$ complex. From the PECs (and Table S11), we observe that the ^{3}MC and ^{5}MC states of $[Fe^{II}(ImP)_2]$ are significantly destabilized as compared to typical Fe(II) polypyridines, such as $[Fe^{II}(bpy)_3]^{2+}$ or $[Fe^{II}(tpy)_2]^{2+}$ and the strong-field $[Fe^{II}(CNC)_2]^{2+}$ complex. The calculated vertical excitations reveal a ^{3}MLCT as the lowest excited state, with ^{1}MLCT and ^{3}MC states 0.38 and 0.33 eV higher in energy, respectively. Also, the energy minimum of the lowest ^{5}MC state is strongly distorted and lies ca. 0.35 eV above the lowest ^{3}MC, suggesting that ^{5}MC is not involved in the deactivation process.

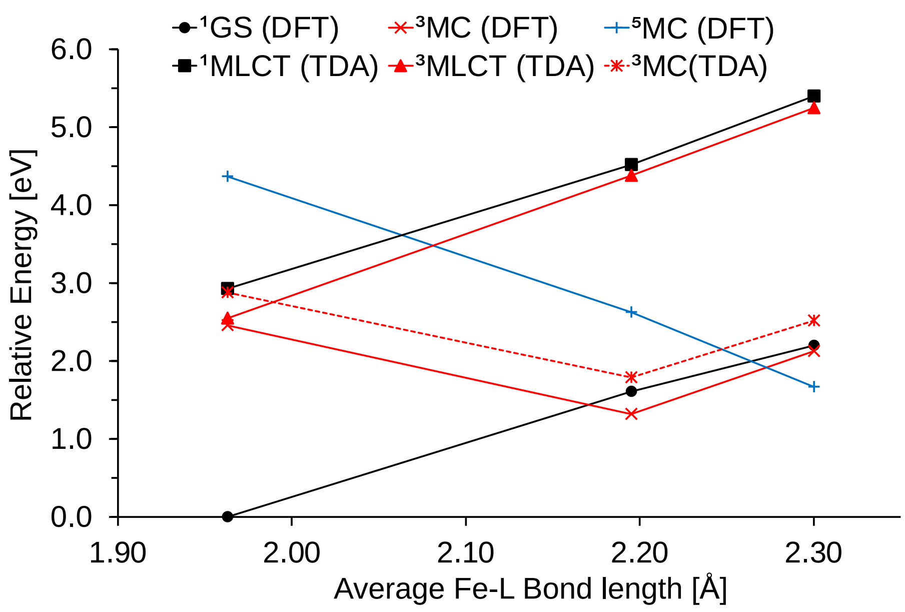

Fig. S27 Potential energy curves for the relevant electronic states of $[Fe^{II}(ImP)_2]$ from energies obtained at the ^{1}GS (R = 1.96 Å), ^{3}MC (R = 2.20 Å), and ^{5}MC (R = 2.30 Å) optimized structures from single point energy calculations at the DFT (^{1}GS, ^{3}MC, ^{5}MC) and TDA levels of theory (^{1,3}MLCT, ^{3}MC) utilizing ^{1}GS as the reference state. The reaction coordinate is given as the average of Fe-L bond lengths at each optimized structure.

Table S9 Quantum chemically calculated relaxed states for $[Fe^{II}(ImP)_2]$ complex at B3LYP+D2/6-311G*, SDD(Fe) in acetonitrile.

	Geometry	E(eV)	Fe spin density	R (Å)
	^{1}GS	0.00	-	1.963
$[Fe^{II}(ImP)_2]$	^{3}MC	1.32	2.024	2.195
	^{5}MC	1.67	3.683	2.300

Stability studies of $[Fe^{II}(ImP)_2]$

From the electronic structure calculations, we found that the ligand detaches in the ^{3}MC of the Fe(II) complex as shown in Fig. S22, which led us to explore whether the solvent (CH_3CN) can bind to the complex at the various spin states (^{1}GS, ^{3}MC, ^{5}MC). Two coordination modes were obtained from the calculations: a) head-on and b) van der Waals (see Fig. S28). Our calculations show that the binding energies of the head-on coordination of CH_3CN to the complex are thermodynamically unfavorable at the various spin states (^{1}GS, ^{3}MC, ^{5}MC). However, for the van der Waals coordination, the ^{3,5}MC states show favorable binding energies, suggesting the possibility of solvent coordination in the excited states. The results show, in general, a favorable coordination of the solvents to the complex in the excited states (^{3,5}MC) compared to the ground state, ^{1}GS.

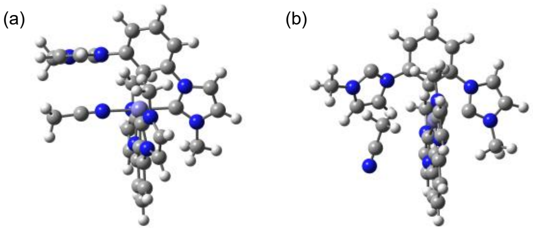

Fig. S28 Geometry optimized structures of the head-on (a) and van der Waals (b) coordination of CH_3CN to the ^{3}MC state of $[Fe^{II}(ImP)_2]$ using B3LYP+D2/6-311G*, SDD(Fe) in CH_3CN.

Table S10 Quantum chemically calculated binding energies ΔE (kcal/mol) of solvent coordination to $[Fe^{II}(ImP)_2]$ complex at B3LYP+D2/6-311G*, SDD(Fe) in acetonitrile at the various spin states.

	Head-on Coordination			Van der Waals Coordination		
	^{1}GS	^{3}MC	^{5}MC	^{1}GS	^{3}MC	^{5}MC
CH_3CN	12.68	8.52	4.14	17.88	-11.50	-8.00

A relaxed potential energy curve (PEC) scan along the Fe-C bond (the carbon bond of the partially detached ligand) was carried out to describe the reaction pathway of the ligand detachment for the ^{1}GS and ^{3}MC states of the van der Waals solvent coordinated complex, [$Fe^{II}(ImP)_2(CH_3CN)$]. For each spin state, a scan was performed along the Fe-C bond length from 4.6 to 2.0 Å in 0.10 Å decrements (see Fig. S13-15). From the PEC, we observe that in the ground state, the thermodynamically favorable structure occurs at Fe-C bond length of 1.9 Å which is ~ 23.0 kcal/mol favorable compared to the detached ligand. In contrast, for the ^{3}MC state, we observe a very small change in energy along the reaction coordinate, in which the thermodynamically favorable structure occurs when the ligand is detached: Fe-C bond length of 4.6 Å. These data confirm that for the Fe(II) complex, ligand detachment is likely to occur in the metal-centered excited states.

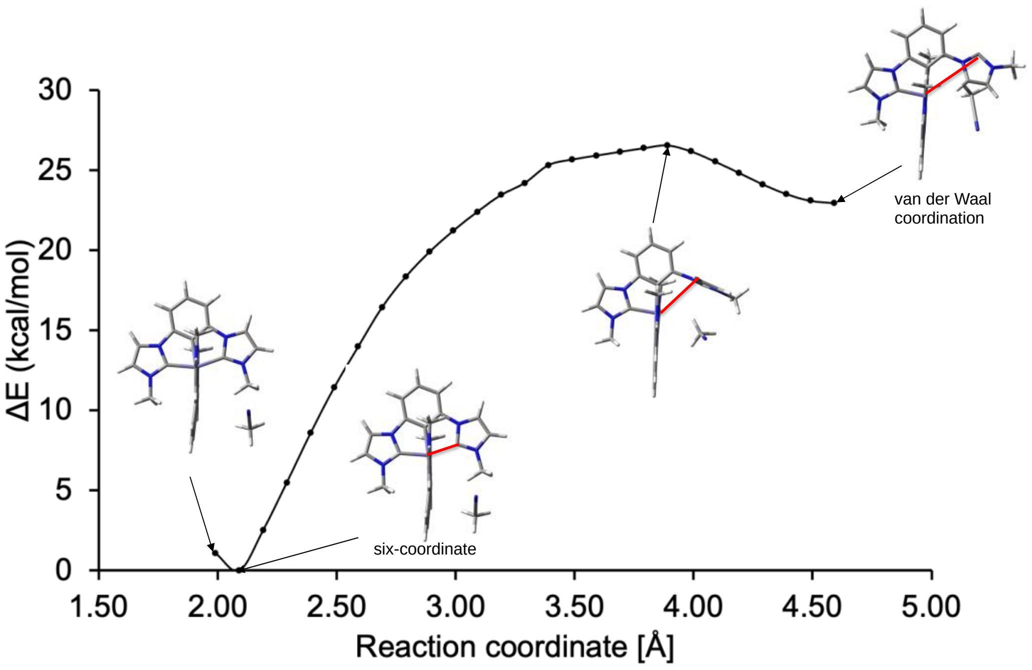

Fig. S29 Potential energy surface scan of the singlet [$Fe^{II}(ImP)_2(CH_3CN)$] (van der Waals coordination of CH_3CN) along one of the Fe-C (carbene) bond (in red) using B3LYP+D2/6-311G*, SDD(Fe) in CH_3CN.

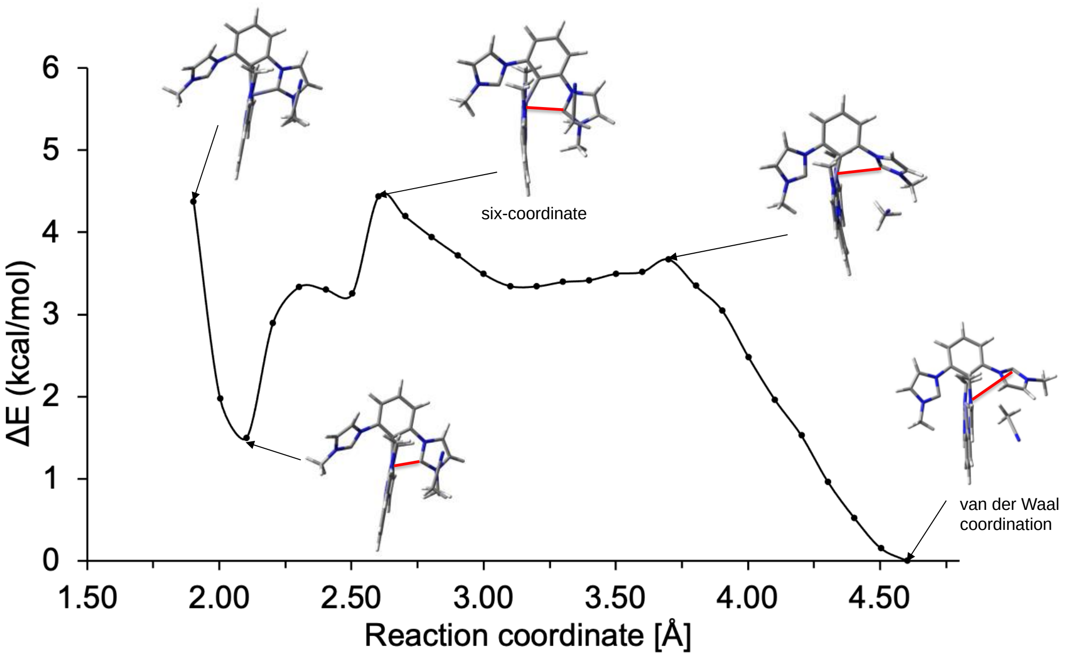

Fig. S30 Potential energy surface scan of the triplet (^{3}MC) [Fe^{II}(ImP)$_2$(CH$_3$CN)] (van der Waals coordination of CH$_3$CN) along one of the Fe-C (carbene) bond (in red) using B3LYP+D2/6-311G*, SDD(Fe) in CH$_3$CN.

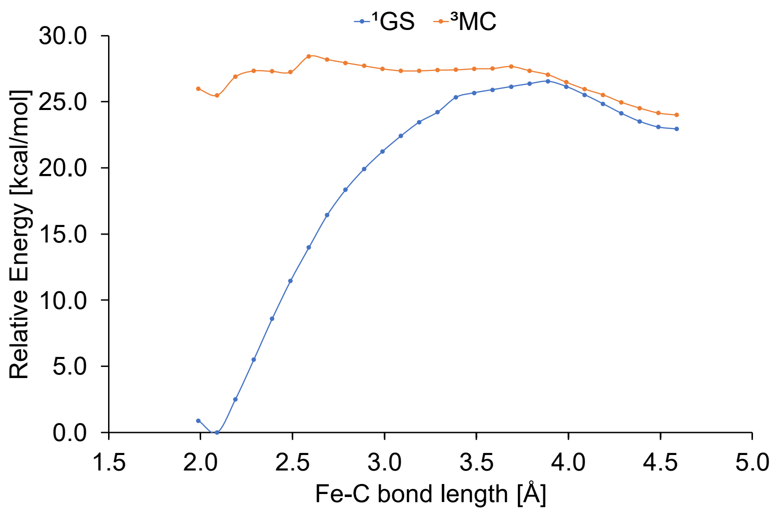

Fig. S31 Potential energy surface scan of the triplet and singlet [Fe^{II}(ImP)$_2$(CH$_3$CN)] (van der Waals coordination of CH$_3$CN) along one of the Fe-C (carbene) bond using B3LYP+D2/6-311G*, SDD(Fe) in CH$_3$CN. All energies are plotted relative to the lowest energy of the ^{1}GS at 2.10 Å.

Potential energy curves of [Fe^{II}(ImP)$_2$(CH_3CN)]

A similar potential energy curve as described in Fig. S27 was carried out for the van der Waals coordinated complex [Fe^{II}(ImP)$_2$(CH_3CN)]. Overall, the ordering of the electronic states is very similar to those of the non-coordinated complex, [Fe^{II}(ImP)$_2$] (see Fig. S27).

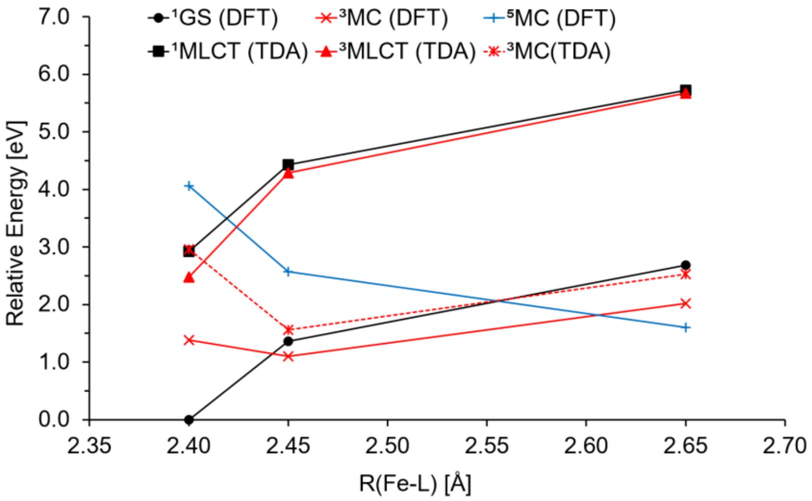

Fig. S32 Potential energy curves for the relevant electronic states of [Fe^{II}(ImP)$_2$(CH_3CN)] from energies obtained at the ^{1}GS (R = 1.96 Å), ^{3}MC (R = 2.20 Å), and ^{5}MC (R = 2.30 Å) optimized structures from single point energy calculations at the DFT (^{1}GS, ^{3}MC, ^{5}MC) and TDA levels of theory (^{1,3}MLCT, ^{3}MC) utilizing ^{1}GS as the reference state. The reaction coordinate is given as the average of Fe-L bond lengths at each optimized structure.

Table S11 Quantum chemically calculated relaxed states for [Fe^{II}(ImP)$_2$(CH_3CN)] complex at B3LYP+D2/6-311G*, SDD(Fe) in acetonitrile.

	Geometry	E(eV)	Fe spin density	R (Å)
	^{1}GS	0.00	-	2.405
[Fe^{II}(ImP)$_2$(CH_3CN)]	^{3}MC	1.36	2.139	2.450
	^{5}MC	2.68	3.786	2.651

Table S12 Vertical excitation energies and character based on natural transition orbitals (NTOs) analysis for $[Fe^{II}(ImP)_2]$. (Excited states with f >= 0.01 and sum of NTO coefficients greater than 0.75 are analyzed)

State	Energy (nm)	Osc. Str.	Coefficient	Hole	Particle	Character
1	428.52	0.020	0.97	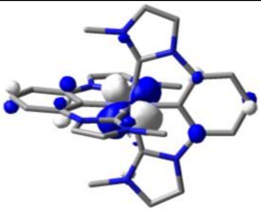	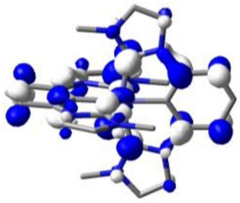	MLCT
2	424.70	0.018	0.96	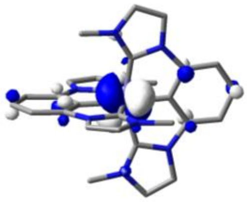	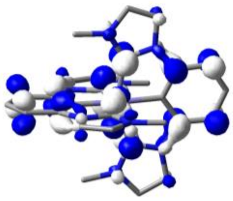	MLCT
4	383.50	0.011	0.95	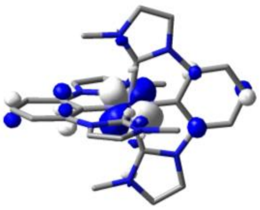	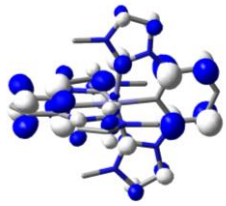	MLCT
5	381.11	0.020	0.98	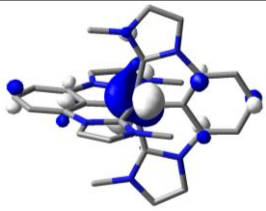	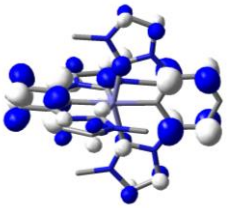	MLCT
7	369.83	0.013	0.42	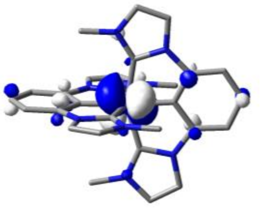	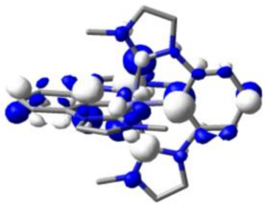	MLCT
			0.35	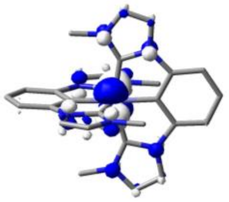	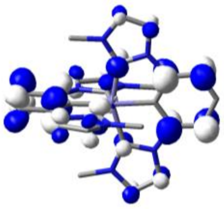	MLCT

9	361.49	0.021	0.68	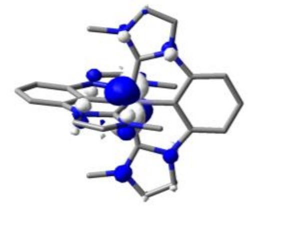	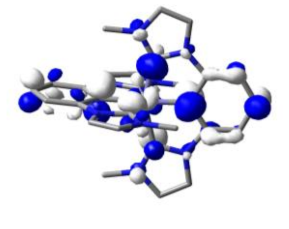	MLCT
			0.30	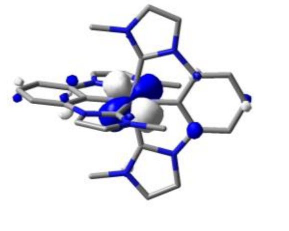	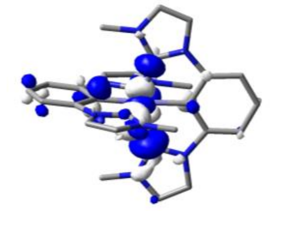	MC
10	360.40	0.029	0.64	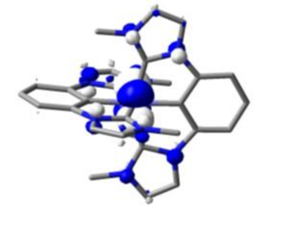	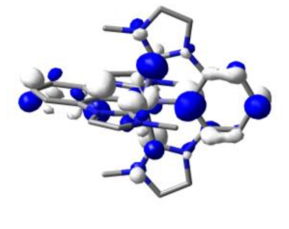	MLCT
			0.24	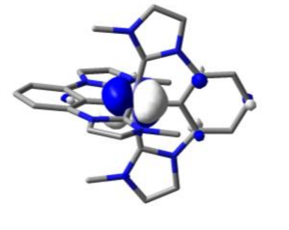	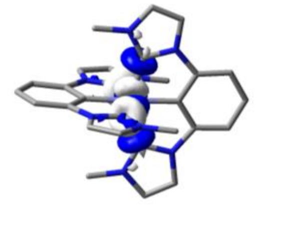	MC
14	345.61	0.211	0.56	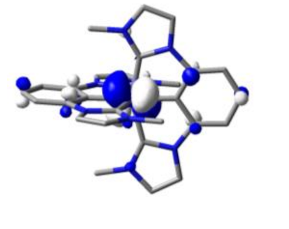	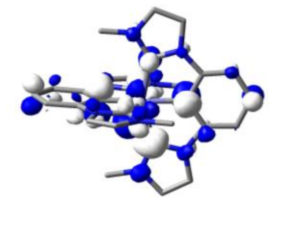	MLCT
			0.33	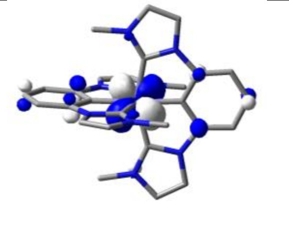	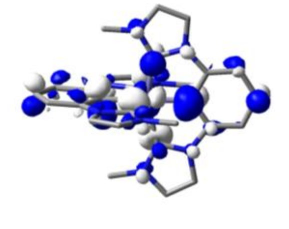	MLCT
17	333.41	0.018	0.55	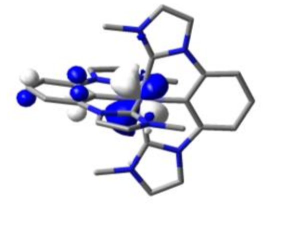	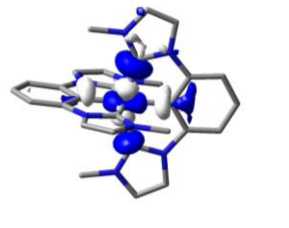	MC

			0.30	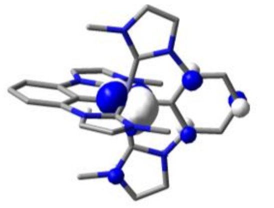	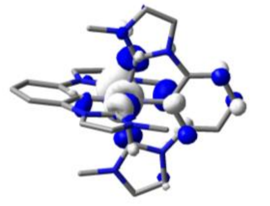	MC

Table S13 Vertical excitation energies and character based on natural transition orbitals (NTOs) analysis for $[Fe^{III}(ImP)_2]^+$. (Excited states with f >= 0.01 and sum of NTO coefficients greater than 0.75 are analyzed)

State	Energy	Osc. Str.	Coefficient	Hole	Particle	Character
6	412.51	0.010	0.81	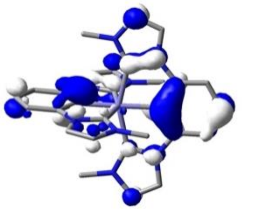	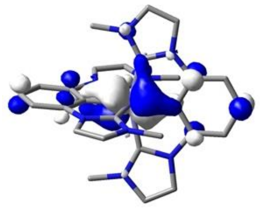	LMCT
17	333.41	0.028	0.49b	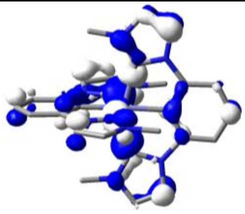	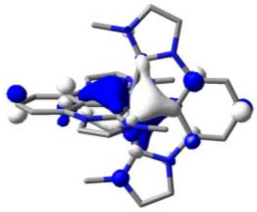	LMCT
			0.16b	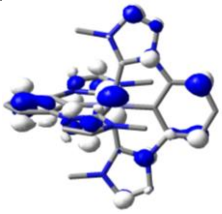	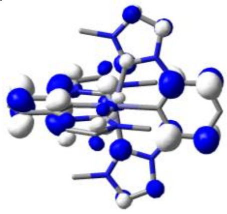	LC
			0.09b	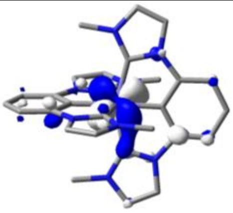	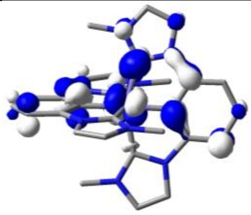	LMCT
			0.08a	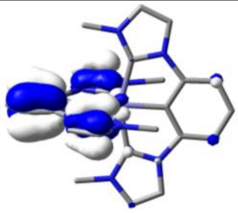	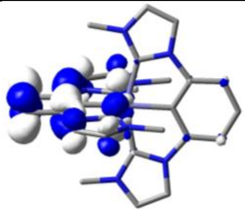	LC

20	323.18	0.018	0.32b	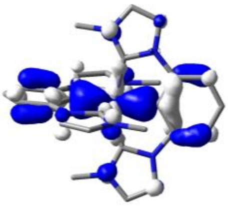	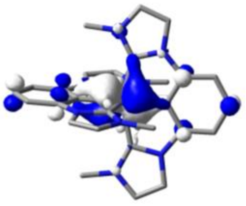	MC<br>LMCT
			0.23b	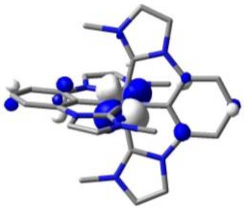	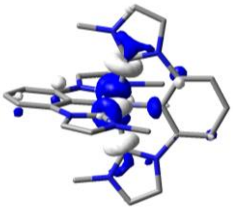	MC
			0.24a	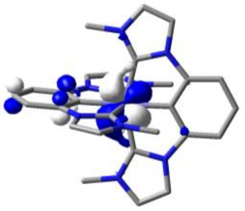	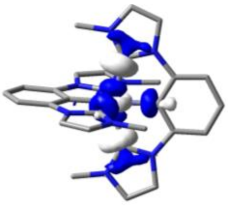	MC
22	314.93	0.018	0.27a	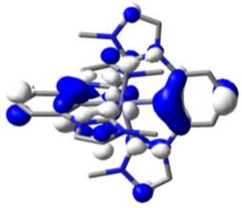	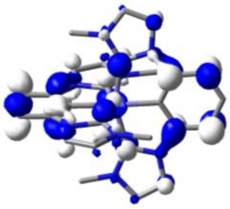	LC
			0.20b	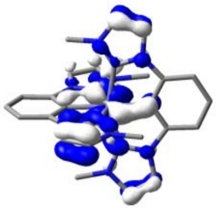	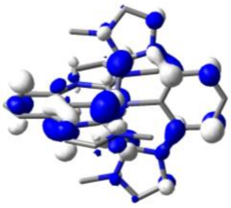	LC
			0.16b	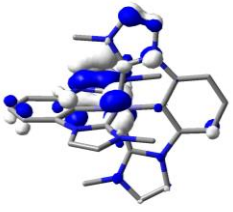	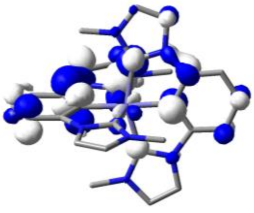	LC
			0.10a	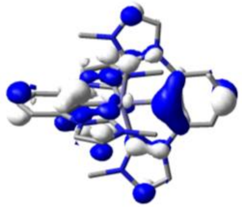	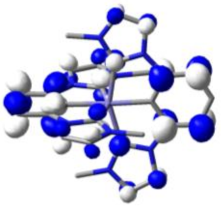	LC

			0.09b	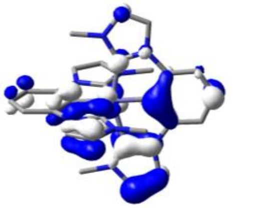	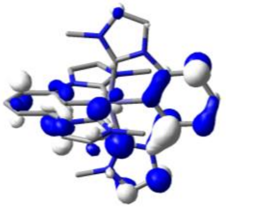	LC
30	296.82	0.030	0.36a	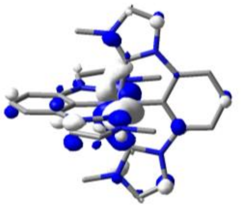	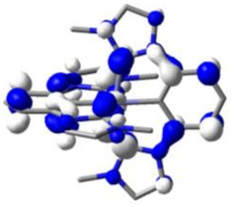	MLCT
			0.16b	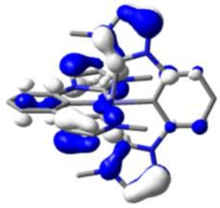	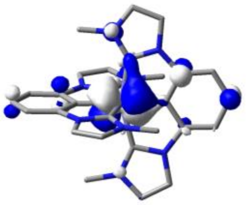	LMCT
			0.13b	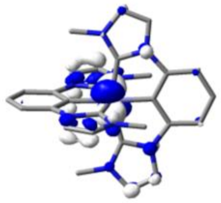	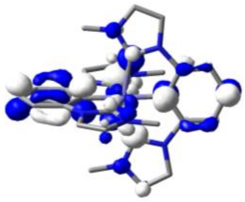	MLCT
			0.13a	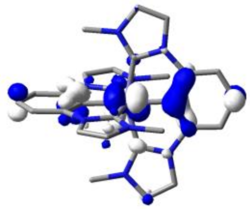	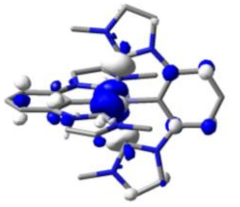	MC
32	290.22	0.044	0.20a	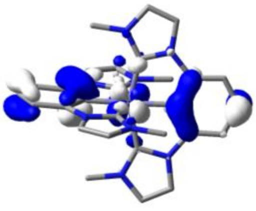	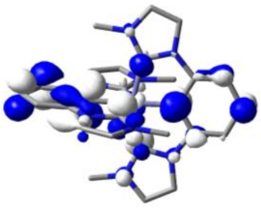	LC
			0.18b	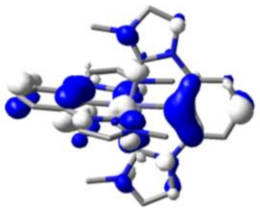	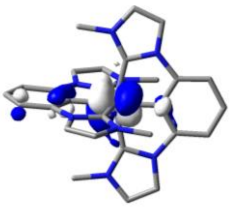	LMCT

			0.16b	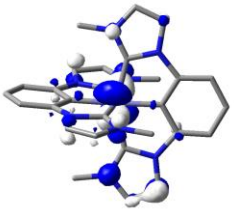	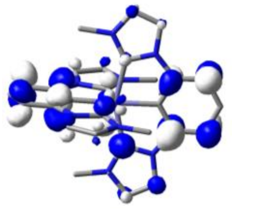	MLCT
			0.13b	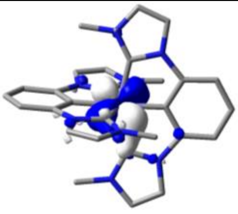	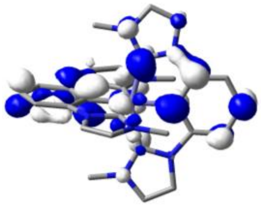	LMCT
			0.09	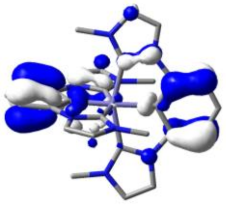	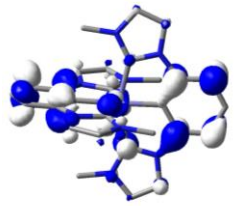	LC
34	285.60	0.027	0.25a	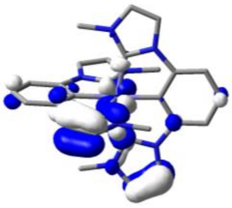	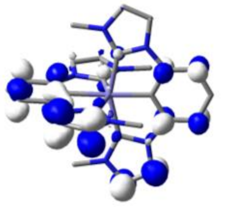	LC
			0.18b	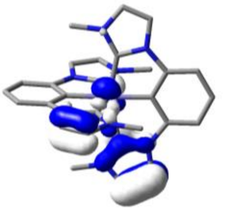	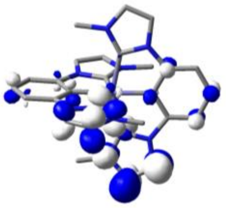	LC
			0.16b	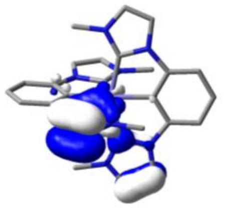	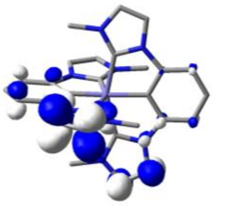	LC
			0.14a	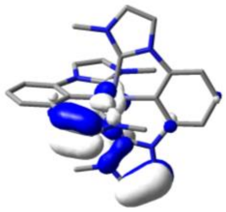	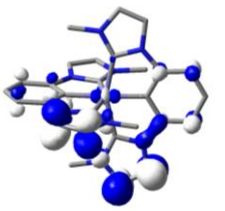	LC

35	284.08	0.049	0.50	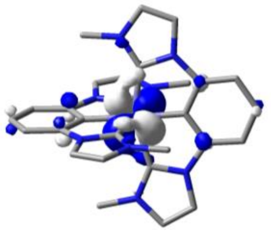	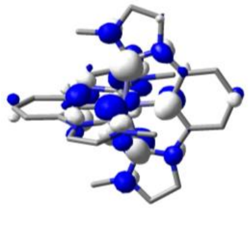	MLCT
			0.11	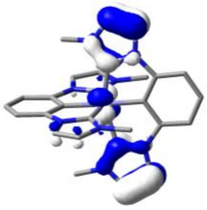	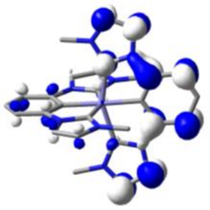	LC
			0.08	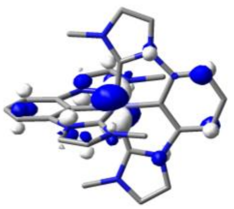	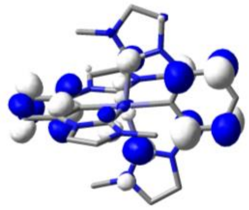	MLCT
			0.07	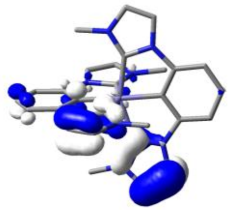	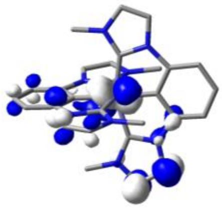	LC<br>LMCT
36	283.19	0.025	0.24a	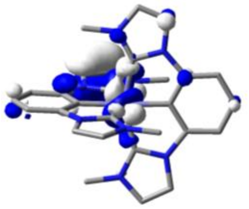	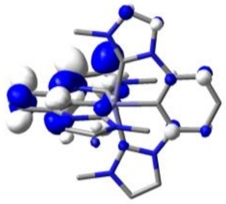	LC
			0.16a	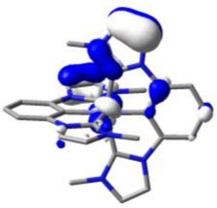	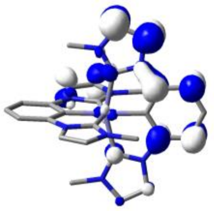	LC
			0.15b	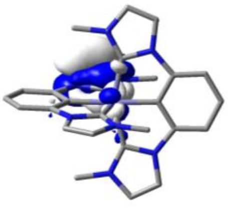	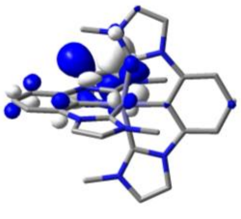	LC

			0.14b	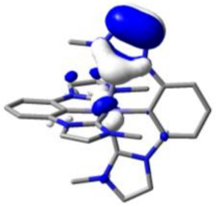	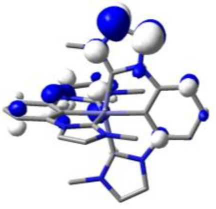	LC
			0.08b	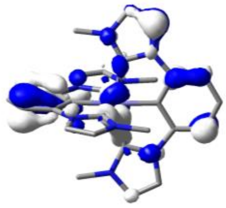	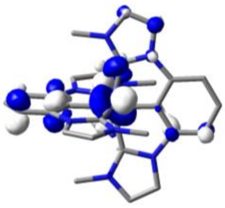	LMCT

References

1. A. T. C. PRO., 2011.
2. G. M. Sheldrick, Crystal structure refinement with SHELXL, *Acta Crystallogr C Struct Chem*, 2015, **71**, 3-8.
3. G. M. Sheldrick, A short history of SHELX, *Acta Crystallogr A*, 2008, **64**, 112-122.
4. O. V. Dolomanov, L. J. Bourhis, R. J. Gildea, J. A. K. Howard and H. Puschmann, OLEX2: a complete structure solution, refinement and analysis program, *Journal of Applied Crystallography*, 2009, **42**, 339-341.
5. D. F. Evans, 400. The determination of the paramagnetic susceptibility of substances in solution by nuclear magnetic resonance, *Journal of the Chemical Society (Resumed)*, 1959, DOI: 10.1039/jr9590002003.
6. E. M. Schubert, Utilizing the Evans method with a superconducting NMR spectrometer in the undergraduate laboratory, *Journal of Chemical Education*, 1992, **69**.
7. J. J. Snellenburg, S. P. Laptenok, R. Seger, K. M. Mullen and I. H. M. v. Stokkum, Glotaran: AJava-Based Graphical User Interface for theRPackageTIMP, *Journal of Statistical Software*, 2012, **49**.
8. A. D. Becke, Density-functional exchange-energy approximation with correct asymptotic behavior, *Phys Rev A Gen Phys*, 1988, **38**, 3098-3100.
9. S. Grimme, Semiempirical GGA-type density functional constructed with a long-range dispersion correction, *J Comput Chem*, 2006, **27**, 1787-1799.
10. R. Krishnan, J. S. Binkley, R. Seeger and J. A. Pople, Self‐consistent molecular orbital methods. XX. A basis set for correlated wave functions, *The Journal of Chemical Physics*, 1980, **72**, 650-654.
11. A. D. McLean and G. S. Chandler, Contracted Gaussian basis sets for molecular calculations. I. Second row atoms, Z=11–18, *The Journal of Chemical Physics*, 1980, **72**, 5639-5648.
12. M. Dolg, U. Wedig, H. Stoll and H. Preuss, Energy‐adjustedabinitiopseudopotentials for the first row transition elements, *The Journal of Chemical Physics*, 1987, **86**, 866-872.
13. G. Scalmani and M. J. Frisch, Continuous surface charge polarizable continuum models of solvation. I. General formalism, *J Chem Phys*, 2010, **132**, 114110.
14. S. I. Gorelsky, AOMix: Program for Molecular Orbital Analysis; version 6.94, http://www.sg-chem.net/).
15. S. I. Gorelsky and A. B. P. Lever, Electronic structure and spectra of ruthenium diimine complexes by density functional theory and INDO/S. Comparison of the two methods, *Journal of Organometallic Chemistry*, 2001, **635**, 187-196.
16. M. J. e. a. Frisch, Gaussian 16, Revision A.03.*Journal*, 2016.
17. M. E. Casida, C. Jamorski, K. C. Casida and D. R. Salahub, Molecular excitation energies to high-lying bound states from time-dependent density-functional response theory: Characterization and correction of the time-dependent local density approximation ionization threshold, *The Journal of Chemical Physics*, 1998, **108**, 4439-4449.
18. R. L. Martin, Natural transition orbitals, *The Journal of Chemical Physics*, 2003, **118**, 4775-4777.
19. S. Hirata and M. Head-Gordon, Time-dependent density functional theory within the Tamm–Dancoff approximation, *Chemical Physics Letters*, 1999, **314**, 291-299.
20. M. J. Peach, M. J. Williamson and D. J. Tozer, Influence of Triplet Instabilities in TDDFT, *J Chem Theory Comput*, 2011, **7**, 3578-3585.
21. S. M. Walter, F. Kniep, L. Rout, F. P. Schmidtchen, E. Herdtweck and S. M. Huber, Isothermal calorimetric titrations on charge-assisted halogen bonds: role of entropy, counterions, solvent, and temperature, *J Am Chem Soc*, 2012, **134**, 8507-8512.
22. G. R. Fulmer, A. J. M. Miller, N. H. Sherden, H. E. Gottlieb, A. Nudelman, B. M. Stoltz, J. E. Bercaw and K. I. Goldberg, NMR Chemical Shifts of Trace Impurities: Common Laboratory

Solvents, Organics, and Gases in Deuterated Solvents Relevant to the Organometallic Chemist, *Organometallics*, 2010, **29**, 2176-2179.
23. J. Steube, A. Kruse, O. S. Bokareva, T. Reuter, S. Demeshko, R. Schoch, M. A. Argüello Cordero, A. Krishna, S. Hohloch, F. Meyer, K. Heinze, O. Kühn, S. Lochbrunner and M. Bauer, Janus-type emission from a cyclometalated iron(iii) complex, *Nature Chemistry*, 2023, **15**, 468-474.
